# Chronological Profiling of Plasma Native Peptides after Hepatectomy in Pigs: Toward the Discovery of Human Biomarkers for Liver Regeneration

**DOI:** 10.1371/journal.pone.0167647

**Published:** 2017-01-06

**Authors:** Kohta Iguchi, Etsuro Hatano, Takashi Nirasawa, Noriyuki Iwasaki, Motohiko Sato, Gen Yamamoto, Kenya Yamanaka, Tatsuya Okamoto, Yosuke Kasai, Naohiko Nakamura, Hiroaki Fuji, Tomohito Sakai, Nobuto Kakuda, Satoru Seo, Kojiro Taura, Kei Tashiro, Shinji Uemoto, Masaya Ikegawa

**Affiliations:** 1 Department of Surgery, Graduate School of Medicine, Kyoto University, Kyoto, Japan; 2 Bruker Daltonics K. K., Yokohama, Japan; 3 Department of Genomic Medical Sciences, Kyoto Prefectural University of Medicine, Graduate School of Medical Science, Kyoto, Japan; 4 Department of Life and Medical Systems, Faculty of Life and Medical Sciences, Doshisha University, Kyoto, Japan; National Center for Toxicological Research, UNITED STATES

## Abstract

Liver regeneration after partial hepatectomy (PHx) is a time-dependent process, which is tightly regulated by multiple signaling cascades. Failure of this complex process leads to posthepatectomy liver failure (PHLF), which is associated with a high rate of mortality. Thus, it is extremely important to establish a useful biomarker of liver regeneration to help prevent PHLF. Here, we hypothesized that alterations in the plasma peptide profile may predict liver regeneration following PHx and hence we set up a diagnostic platform for monitoring posthepatectomy outcome. We chronologically analyzed plasma peptidomic profiles of 5 partially hepatectomized microminipigs using the ClinProt^TM^ system, which consists of magnetic beads and MALDI-TOF/TOF MS. We identified endogenous circulating peptides specific to each phase of the postoperative course after PHx in pigs. Notably, peptide fragments of histones were detected immediately after PHx; the presence of these fragments may trigger liver regeneration in the very acute phase after PHx. An N-terminal fragment of hemoglobin subunit α (3627 *m/z*) was detected as an acute-phase-specific peptide. In the recovery phase, the short N-terminal fragments of albumin (3028, 3042 *m/z*) were decreased, whereas the long N-terminal fragment of the protein (8926 *m/z*) was increased. To further validate and extract phase-specific biomarkers using plasma peptidome after PHx, plasma specimens of 4 patients who underwent PHx were analyzed using the same method as we applied to pigs. It revealed that there was also phase-specificity in peptide profiles, one of which was represented by a fragment of complement C4b (2378 *m/z*). The strategy described herein is highly efficient for the identification and characterization of peptide biomarkers of liver regeneration in a swine PHx model. This strategy is feasible for application to human biomarker studies and will yield clues for understanding liver regeneration in human clinical trials.

## Introduction

Liver regeneration is a complex event and is tightly regulated by multiple signaling mechanisms that become activated in a time-dependent fashion [1]. To study the mechanisms underlying liver regeneration, partial hepatectomy (PHx), resulting in the removal of approximately 70% of the liver, has been widely utilized in experimental animals such as mice, rats, and pigs. In rats, the first peak of DNA synthesis in hepatocytes occurs at about 24 hours (h), with a smaller peak between 36–48 h. After that, the new vascular branches are formed, the extracellular matrix is synthesized, and finally, normal liver histology is reestablished 8–10 days after PHx [2]. During this time-dependently regulated process, posthepatectomy liver failure (PHLF), which results from a failure of liver regeneration, develops with high incidence in patients after PHx [3]. PHLF occurs when patients with poor liver function are exposed to excessive surgical stress including extensive blood loss or massive hepatectomy. The symptoms of PHLF include prolonged fluid retention and coagulation disorders within a week after PHx, and it is closely associated with postoperative mortality [4]. Thus, it is crucial to establish biomarkers of liver regeneration to improve the surgical outcomes of patients with liver diseases.

In recent years, mass-spectrometry (MS)-based protein identification has enabled us to map the liver-tissue specific proteome in combination with tissue-specific gene expression and antibody-based immunohistochemistry [5]. Because no less than 33% of the proteins expressed in the liver are classified as secreted proteins [5], serum or plasma is expected to become a promising source of biomarkers of liver diseases. Prior efforts in the search for serum and plasma protein biomarkers utilized gel-based separation technologies, which cannot readily separate and distinguish molecules of less than 10 kDa in size [6]. The application of MS to analyze such a low molecular weight range of molecules in blood has succeeded in providing disease-specific peptide signatures in patients with various cancers originating from ovary, prostate, breast, pancreas, and liver [7–11]. Precisely defining the chronological changes in the peptide profile of plasma following PHx using MS approach might also provide a sensitive biomarker of liver regeneration that cannot be detected using transcriptome-based or antibody-based approaches.

In the present study, we performed plasma peptidomic analyses after 70% PHx in pigs, which closely resemble man in anatomy, physiology, and genetics [12]. For detecting plasma peptides, we applied a magnetic bead separation and matrix-assisted laser desorption/ionization time-of-flight (MALDI-TOF) MS approach, which is a robust, precise, and rapid technique for the investigation of complex blood samples with a detection range of 12 orders of magnitude in protein abundance [13, 14]. The patterns of endogenous peptides circulating in plasma were chronologically analyzed and representative markers of each phase after PHx were identified. The postoperative course after PHx could be clearly delineated by the plasma peptidomic pattern. Finally, this strategy was successfully applied to clinical samples, and some native peptides that could be useful in monitoring the postoperative course were identified.

## Materials and Methods

### Animals

Five female specific pathogen-free microminipigs aged 1.5–2.0 years and weighing 20–22 kg were obtained from Fuji Micra (Shizuoka, Japan) [15]. Three pigs were used as a first cohort and the remaining 2 as a second cohort. After an overnight fast, pigs were sedated with xylazine HCl (0.3 mg/kg), midazolam (0.2 mg/kg), and atropine sulfate (0.025 mg/kg) intramuscularly, and intratracheal intubation was performed. Under general anesthesia with a mixture of oxygen/air (FiO2 0.4) and sevoflurane (1–1.5%), a 70% PHx procedure (removal of the left lateral lobe and medial lobe of the liver) was successfully performed. The pigs were housed in cages under a 12:12-h light/dark cycle at 22°C. The physical conditions of the animals were checked every day until postoperative day 7 (168 h), when they underwent laparotomy under general anesthesia. After sampling of blood and liver, the animals were euthanized by cutting the inferior vena cava under deep anesthesia using sevoflurane. If abnormal symptoms such as general fatigue, decreased activity, frequent vomiting, or respiratory distress were observed, the pigs were euthanized before the predetermined day. As a result, all animals showed uneventful postoperative course until postoperative day 7. The remnant liver exhibited good regeneration with ~3-fold increase in its volume [16]. Blood samples were collected before (pre), at 0 minutes (min) and at 1, 3, 6, 24, 48, 72, 96, 120, 144, and 168 h after PHx through a central venous (CV) catheter cannulated in the right external jugular vein. Blood samples from portal vein (PV) were also collected before, at 0 min, and at 1, 3, and 168 h after PHx through a PV catheter cannulated from the jejunal branch of the superior mesenteric vein. The blood samples for plasma were collected into blood collection tubes containing 3.2% (w/v) sodium citrate. Serum and plasma were collected after centrifugation of blood samples at 2190 and 1720 × *g* for 10 min, respectively, and were stored at −80°C for later measurements. All animal experiments were conducted under a protocol approved by the animal research committee of Kyoto University (reference number: Med Kyo 14243), and all animals were cared for in accordance with Guide for the Care and Use of Laboratory Animals from the National Institutes of Health (USA).

### Human Samples

Plasma samples of 10 patients who underwent PHx at Kyoto University Hospital in 2014 were prospectively collected. To search for biomarkers of normal liver regeneration after major hepatectomy, 4 patients were analyzed; these patients underwent major hepatectomy without pathological liver fibrosis (F0 or F1) [17], and had an uneventful postoperative course with normal liver regeneration (S1 Table). Major hepatectomy refers to resection of more than 3 segments defined according to Couinaud’s classification [18]. Blood samples were collected from peripheral veins at 0 min, 24, 48, and 168 h after PHx. Sample preparation and data measurement were performed using the same method as we applied to pigs, as described below. This study was approved by the Ethics Committee of Graduate School and Faculty of Medicine Kyoto University (C-512). Written informed consent was acquired from all patients.

### Peptide Purification with Magnetic Beads

A 3-μl aliquot of the serum or plasma was purified using magnetic beads based on hydrophobic interaction chromatography (C8-coated magnetic beads, MB-HIC, Bruker Daltonics GmbH, Bremen, Germany). The C8 magnetic beads are superparamagnetic, silica-based particles, and are surface-derivatized with common reversed-phase ligands of 8 carbon alkyl chains. In brief, 3 μl of the serum or plasma was diluted with 10 μl of binding buffer (MB-HIC kit, Bruker), followed by the addition of 5 μl of magnetic beads. The solution was then carefully mixed by pipetting in and out five times. After 1 min, the supernatant was separated from the magnetic beads in a magnetic separator. This was followed by 3 washing steps with 100 μl of wash buffer (MB-HIC kit, Bruker), and the supernatant was removed each time. After 1 min in 10 μl of elution buffer (stabilization buffer (MB-HIC kit, Bruker) in 50% acetonitrile), the magnetic beads were separated in the magnetic separator from the elution buffer. For MS analysis, 1 μl of the bead eluate was mixed with 10 μl of matrix solution (0.3 g/l α-cyano-4-hydroxycinnamic acid (Bruker) in 2:1 ethanol/acetone), and 1 μl of the mixture was then spotted in quadruplicate on an MTP AnchorChip 600/384 target (Bruker). All these processes were performed with the aid of ClinProt Robot (Bruker). Tryptic digestion was not performed.

### Mass Spectrometry

Samples applied to the AnchorChip were analyzed using an AutoFlex^TM^ II MALDI-TOF MS operating in positive-ion linear mode (Bruker). To generate a mass spectrum, 1500 laser shots were acquired from random positions for each matrix spot. Four independent spectra were acquired for each spot. Acquisition was controlled by flexControl 3.0 software (Bruker) using the AutoXecute function and fuzzy control of laser intensity. The analysis was performed at mass to charge ratio (*m/z*) of 2000−20000 and a signal-to-noise threshold of 5. Spectra were externally calibrated using a mixture of standardized protein/peptide calibrants (ClinProt Standard, Bruker).

### Statistical Analysis

The resulting spectra were analyzed using ClinProTools 2.2 bioinformatic software (Bruker) [19]. ClinProTools was used to carry out comparative analysis of peak intensities between postoperative days and to calculate corresponding statistics. When comparing 2 and more than 2 groups, we used the Wilcoxon−Mann−Whitney test or the Kruskal−Wallis test, respectively. In ClinProTools, a receiver operating characteristic (ROC) curve was generated for each peak within peak calculation for separating 2 groups. The area and the intensity of the peak represented the threshold that was used to reach the separation into 2 groups. Multivariate analysis techniques, including principal component analysis (PCA) and the support vector machine (SVM) algorithm were employed to extract, display, and rank the variance within each data set. PCA selects the principal components (PCs) that can separate the samples into homogeneous clusters and can be visualized in 3-dimensional plots in which calculated values for the top PCs serve as *x*, *y*, and *z* axes [20]. The SVM algorithm, another machine-learning approach, was applied to the mass data to select clusters of signals able to discriminate between groups [19]. ClinProTools identified several features as useful for classifying 2 time points automatically by determining the best number of peaks (up to 25 peaks) to be integrated in the model by an internal iteration. Cross-validation accuracy is the percentage of data correctly classified. In pig experiments, peptidomic pattern analyses including PCA and SVM were performed and representative peaks were detected in the first cohort. In the second cohort, those peaks were validated. Multiple experiments were performed and representative data were shown in the figures. The mass numbers of proteins were described as average values with possible distribution of ±3 Da.

### Reversed Phase-High Performance Liquid Chromatography (RP-HPLC)

Prior to RP-HPLC fractionation, the C8-purified eluate samples were mixed with 20 μl of 0.1% (v/v) trifluoroacetic acid and centrifuged in a vacuum to remove organic solvent. A 10-μl sample was injected in every run. RP-HPLC separations were performed using an easy-nLC II nanoHPLC system (Bruker) equipped with a PepSwift^TM^ Monolithic PS-DVB column (0.2 × 50 mm, Dionex, Osaka, Japan). Solvent A was 0.1% (v/v) trifluoroacetic acid in Milli-Q^®^ purified water, and Solvent B was 100% acetonitrile. The flow rate was 900 nl/min. The following gradient was used for 0–60 min; 5–50% B, 60–61 min; 50–95% B, 61–64 min; 95% B constant. The HPLC eluent was mixed with LC-MALDI matrix (α-cyano-4-hydroxycinnamic acid) at the flow rate of 1.67 μl/min via MicroTee fittings (Upchurch Scientific, Oak Harbor, WA, USA), then deposited on an MTP AnchorChip 384 target with a spot pitch of 800 μm (Chip 0) by a PROTEINEER fc II^TM^ fraction collector (Bruker). Sample spots were deposited every 15 s, and a total of 248 spots were collected for each HPLC run. Calibration samples were manually spotted on calib. Anchor position (Chip 1) to use automatic calibration processing.

### MS/MS-based Sequencing and Identification

Automatic MS/MS experiments were performed using an ultrafleXtreme^TM^ MALDI-TOF/TOF MS (Bruker). Spot selection, automatic MS/MS mass filtering, and Mascot database searching (ver. 2.4.1, Matrix Science, Boston, MA, USA) were commanded by WARP-LC 1.3 (Bruker). Data processing and assessment of MS/MS results were performed using ProteinScape^TM^ 3.1 (Bruker). For measurement, MS spectra were automatically acquired on the ultrafleXtreme^TM^ instrument in the positive reflector mode under the control of Compass 1.3 and WARP-LC 1.3 software (Bruker), and automatic MS calibration processing was controlled by the AutoXecute function. The detection range was 1000–4000 *m/z*. Detected peptide compounds with a signal-to-noise ratio higher than 10 were automatically subjected to LIFT^TM^-TOF/TOF experiments triggered with WARP-LC [21]. After each measurement, queries were automatically transferred to the ProteinScape 3.1 server. Database searches were also automatically achieved using an in-house Mascot server. Mascot search conditions were as follows; Database: SwissProt, Taxonomy: other Mammalia, Enzyme: none, Modifications: Acetyl (K), Delta: H (2) C (2), Methyl (K), Oxidation (M), Phospho (ST), tolerance: 50 ppm in MS, 0.8 Da in MS/MS.

## Results

### Discrimination of Plasma Peptide Profiles among Postoperative Time Points

**Fig 1:**
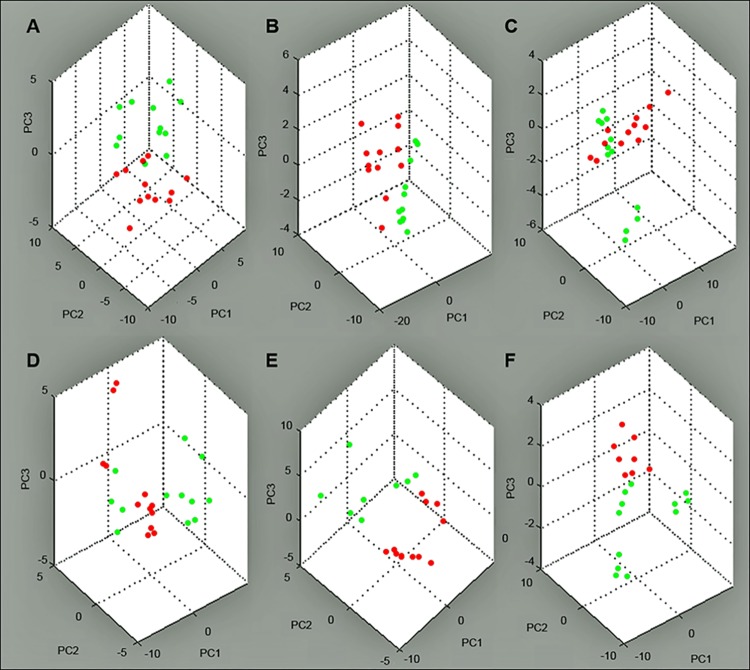
Representation of the principal components generated from the data set of a first cohort. (A) 0 min (red) *vs*. 24 h (green), (B) 24 h (red) *vs*. 48 h (green), (C) 48 h (red) *vs*. 72 h (green), (D) 72 h (red) *vs*. 96 h (green), (E) 96 h (red) *vs*. 120 h (green)*, (F) 120 h (red)* *vs*. 168 h (green). For each plasma sample, 4 measurements were automatically performed. The detailed data used for the analysis were provided as pseudogel and stack view of the spectra in S1 Fig. *Three pigs were analyzed, except at 120 h, at which time point data were missing for 1 pig. PC, principal component.

PCA showed that each successive timing was clearly separated by 3 PCs (0 min *vs*. 24 h, 24 h *vs*. 48 h, 96 h *vs*. 120 h, 120 h *vs*. 168 h) except 48 h *vs*. 72 h, and 72 h *vs*. 96 h. (Fig 1) This suggests that the peptide profile in plasma dramatically changed during each perioperative period as defined above, reflecting the physiological process of liver regeneration after PHx.

### Classification of Postoperative Time Course by SVM

**Fig 2:**
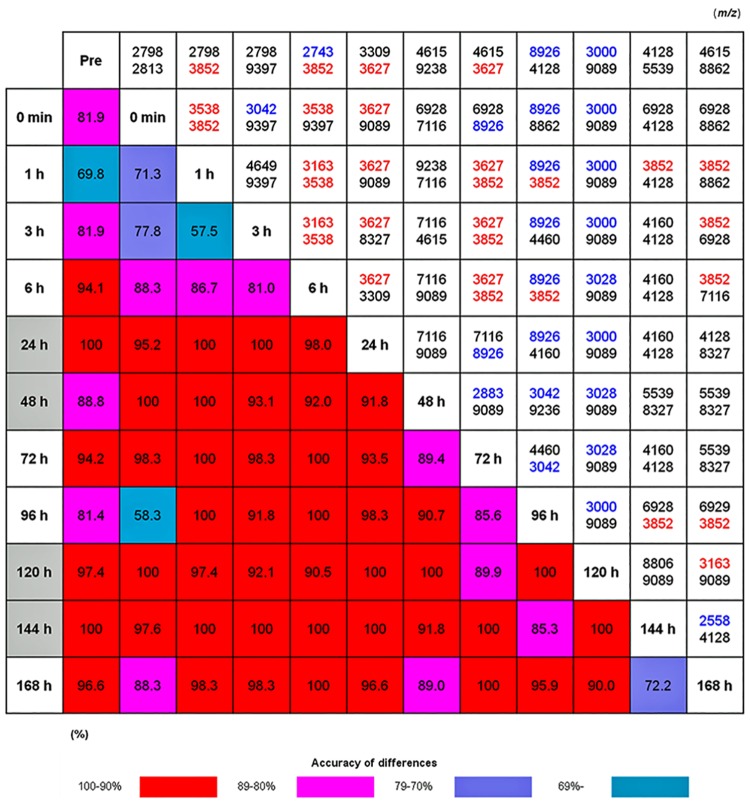
Heat map of the SVM analysis to differentiate each time point after PHx. The lower left half of the figure provides the results of cross-validation analyses to estimate the success rate of the SVM model in separating user-defined groups of spectra. The accuracy is denoted by color: 100–90% (red); 89–80% (pink); 79–70% (purple), and < 69% (blue). The upper right half of the figure describes the *m/z* of the top 2 peaks that most efficiently discriminated each time point with statistical significance. The detailed data used for the analysis were provided as pseudogel and stack view of the spectra in S1 Fig, and the statistical data were as S2 Table. The identified albumin peaks are shown in blue (2558: 25–45^*1^; 2743: 26–48; 2883: 25–48^*2^; 3000: 25–49^*3^; 3028: 25–49^*4^, 3042: 25–50, 8926: 25–103), and the hemoglobin subunit α is shown in red (3163: 1–32; 3538: 110–141; 3627: 1–36; 3852: 107–141). *1: Ala->Pro: 4, *2: Ala->Pro: 20, *3: Gly -> Ser: 22, *4: Q -> deamidated: 18, SVM, support vector machine; *m/z*, mass to charge ratio.

The lower left half of Fig 2 showed the results of cross-validation analyses in which generated SVM models were used to compare every 2 groups. For example, the spectra patterns at 1 and 3 h were not clearly separated, having a cross-validation accuracy of 57.5%. However, those at 6 and 24 h were quite different, with a cross-validation accuracy of 98.0%. Of note, studying the heat map diagonally from upper left to lower right revealed that the plasma peptidome changed in a biphasic manner after PHx. High cross-validation accuracy was detected at 2 phases, including around 24 h and 120 h. Based on these results, we defined the time points of around 24 h and 120 h as the acute and recovery phases, respectively.

Comparative analysis of spectra between groups revealed a number of peaks with significant differences in intensity. The right upper half of Fig 2 detailed the top 2 peaks enabling discrimination between every 2 groups. For example, when we compared the samples taken at 1 and 96 h after PHx, the peaks whose abundance was most different between these time points were at 8926 and 3852 *m/z*. Liquid chromatography-matrix-assisted laser desorption/ionization time-of-flight tandem mass spectrometry (LC-MALDI-TOF/TOF MS) analysis identified several peptides, including albumin (ALB; blue letters in Fig 2) and hemoglobin subunit α (HBA; red letters in Fig 2). This result suggests that peptide fragments of HBA and ALB were the representative peaks in the acute and recovery phases, respectively.

### Marker Peak Extraction by ROC Curve Analysis

To clarify single peaks as representative markers of the acute and recovery phases, ROC curve analysis was performed to compare 0 min *vs*. 24 h, and 24 h *vs*. 120 h. (Table 1) This revealed that the peaks at 3627, 4160, 8327, and 9089 *m/z* had the highest area under the curve (AUC) values during the acute phase, while the peaks at 3028, 3042, 3627, 6929, 8327, and 8926 *m/z* had the highest AUC values during the recovery phase. This result was validated by the second cohort analysis. Among these discriminatory peaks, those at 3627 and 8926 *m/z* showed the highest AUC values during the acute and recovery phases, respectively. LC-MALDI-TOF/TOF MS analysis identified the peaks at 3627 and 8926 *m/z* as the N-terminal fragment of HBA (position: 1–36) and ALB (position: 25–103), respectively.

**Table 1:** AUC determined by ROC curve analysis for each peak used to compare 0 min *vs*. 24 h (acute phase), and 24 h *vs*.120 h (recovery phase).

*m/z*	0 min *vs.* 24 h	24 h *vs.* 120 h
**3028**		1/0.80
**3042**		0.86/0.98
**3627**	1/1	0.96/0.98
**4160**	0.89/0.92	
**6929**		0.90/1
**8327**	0.96/1	0.99/1
**8926**		1/1
**9089**	0.90/0.94	

Peaks with AUC ≧0.8 were listed in the table. Values are given as first cohort/second cohort. The identified peaks were as follows; 3028 (ALB: 25–49, Q -> deamidated: 18), 3042 (ALB: 25–50), 3627 (HBA: 1–36), 8926 (ALB: 25–103). AUC, area under the curve; ROC, receiver operating characteristics; *m/z*, mass to charge ratio; ALB, albumin; HBA, hemoglobin subunit α.

### Other Marker Peak Candidates with Characteristic Alterations

**Fig 3:**
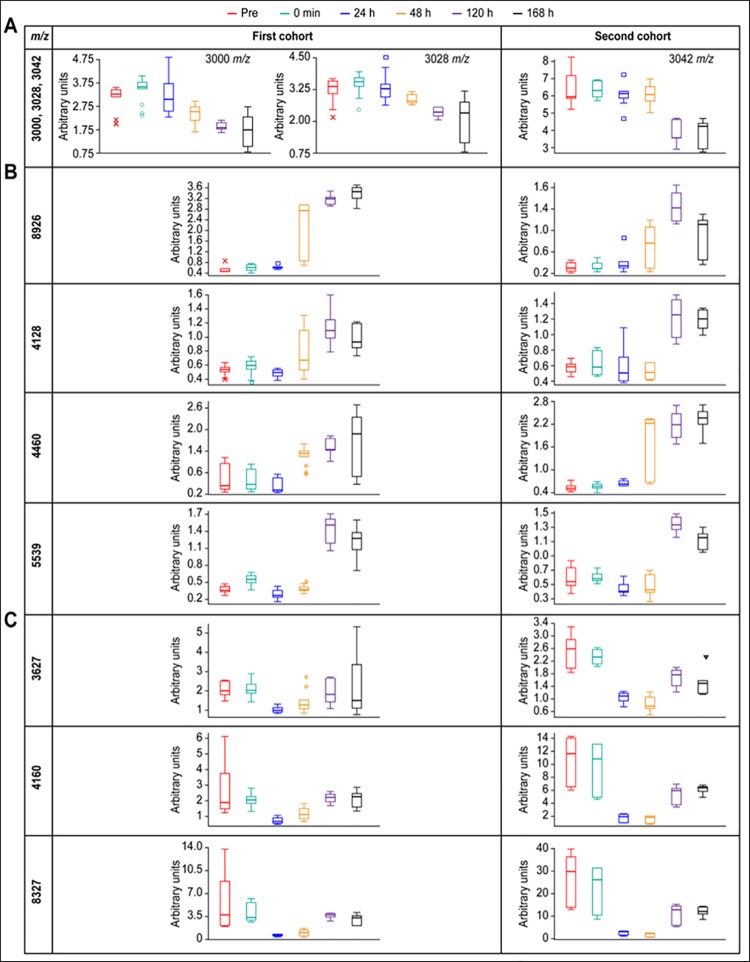
Box-and-whisker plots of the representative markers for discriminating the phases after PHx. The discriminatory peaks were classified into 3 groups according to the patterns of change; peptides (A) down-regulated at recovery phase; (B) up-regulated at recovery phase; (C) down-regulated at acute phase. Pre (red), 0 min (light green), 24 h (blue), 48 h (yellow), 120 h (purple), 168 h (black). The top and bottom end marks of the plot indicated the maximum and minimum peak area/intensity within a given class. The box indicated the 25%-quartile (bottom) and the 75%-quartile (top) and the horizontal intersection denotes the median. The statistical data were detailed in S3 Table. The plot was drawn on a unique scale independent of the peak intensity scale. The identified peaks were as follows; 3000: ALB, 25–49^*1^; 3028: ALB, 25–49^*2^, 3042: ALB, 25–50, 8926: ALB, 25–103, 3627: HBA, 1–36. *1: Gly -> Ser: 22, *2: Q -> deamidated: 18. *m/z*, mass to charge ratio; ALB, albumin; HBA, hemoglobin subunit α.

When 6 time points (pre, 0 min, and 24, 48, 120, and 168 h) were compared, several peaks were determined to become useful classifiers of the perioperative time points (Fig 3). In the first cohort analysis, the peaks at 3000 and 3028 *m/z* were down-regulated in the recovery phase; the peaks at 8926, 4128, 4460, and 5539 *m/z* were up-regulated in the recovery phase; and the peaks at 3627, 4160, and 8327 *m/z* were down-regulated in the acute phase. These observations were fully replicated in the second cohort. Among the detected peaks, the peaks at 3000 and 3028 m/z were identified as short N-terminal fragments of ALB (position: 25–49 amino acids), and the peak at 8926 *m/z* was a long N-terminal fragment of ALB (position: 25–103 amino acids). This result suggests that different peptide fragments, even those derived from the same protein, show different patterns of change after PHx.

### Comprehensive Identification of Swine Plasma Peptides after PHx by LC-MALDI-TOF/TOF MS

To further identify the circulating peptides at various time points following 70% PHx, we performed a comprehensive identification analysis using the C8-purified eluate of the plasma samples at 0 min, and 24, 48, and 120 h. The results revealed that 75, 66, 24, and 20 peptides were identified with mascot scores of more than 50 at these time points, respectively (S4 Table), and these peptides were found to be fragments of 18, 7, 4, and 4 kinds of proteins, respectively (Table 2). Notably, more fragments were phase-specifically identified at 0 min (immediately after PHx, very acute phase) than other timings. Their parent proteins included histone family proteins (H1, H2A, H2B, H3, and H4). Representative MS/MS spectra were provided as S2 Fig.

**Table 2:** Swine Plasma peptides after PHx detected by magnetic bead-based LC-MALDI-TOF/TOF MS analyses.

**Time**	**Protein**
**0 min**	Serum albumin
	Basic proline-rich protein
	Histone H1.2^b^
	Histone H1.3^b^
	Histone H1.3^o^
	Histone H1.4^o^
	Histone H2A type 1^b^
	Histone H2B type 1^b^
	Histone H3.1^b^
	Histone H4
	Non-histone chromosomal protein HMG-17
	Hemoglobin subunit α
	Vasodilator-stimulated phosphoprotein^c^
	Serotransferrin
	Apolipoprotein C-III
	Ryanodine receptor 1
	60S ribosomal protein L4
	Coatomer subunit beta
**24 h**	Basic proline-rich protein
	Serum albumin
	Hemoglobin subunit α
	Vasodilator-stimulated phosphoprotein^c^
	Serotransferrin
	Radixin^b^
	Ribosome-binding protein 1^c^
**48 h**	Basic proline-rich protein
	Serum albumin
	Ryanodine receptor 1
	26S proteasome non-ATPase regulatory subunit 8^b^
**120 h**	Basic proline-rich protein
	Serum albumin
	Hemoglobin subunit α
	Vasodilator-stimulated phosphoprotein^b^

Peptides with mass range of 1000–4000 *m/z* were analyzed. Among them, those with mascot score of more than 50 were described in the table. Peptides were annotated using the protein sequence database of pigs. Those annotated using other species were indicated with superscripts b (*Bos Taurus*), c (*Canis familiaris*), or o (*Oryctolagus cuniculus*). PHx, partial hepatectomy; LC-MALDI-TOF/TOF MS, liquid chromatography-matrix-assisted laser desorption/ionization time-of-flight tandem mass spectrometry.

### Profiling Human Plasma Peptides after PHx

**Fig 4:**
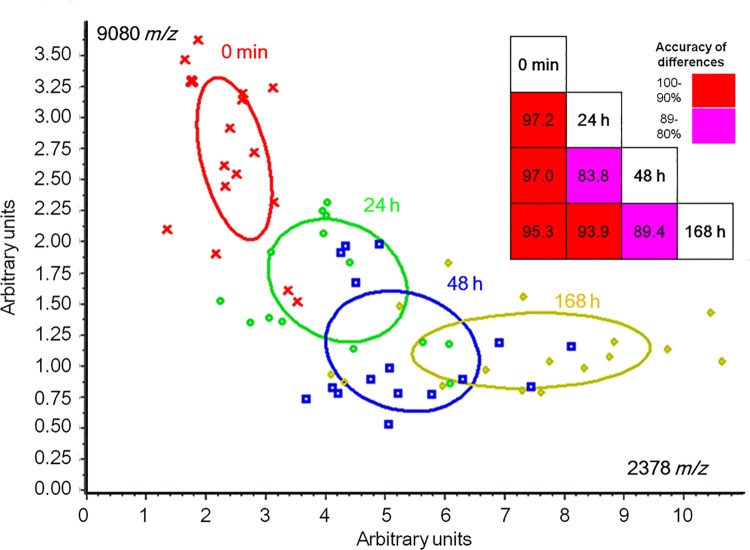
Profiling of human plasma native peptides after PHx (n = 4). The upper right part of the figure described the results of cross-validation analyses to estimate the success rate of the SVM model in separating user-defined groups of spectra. The accuracy is denoted by color: 100–90% (red); 89–80% (pink). The 2-dimensional distribution plot analysis revealed the top 2 representative peaks, which discriminated the peptidomic patterns at 0 min (red), 24 (light green), 48 (blue), and 168 h (yellow) (*x* axis: 2378 *m/z*, P-value: <0.000001, *y* axis: 9080 *m/z*, P-value: <0.00001). LC-MALDI-TOF/TOF MS analyses identified the peak at 2378 *m/z* as a fragment derived from complement C4b (position:1429–1449). PHx, partial hepatectomy; SVM, support vector machine; *m/z*, mass to charge ratio, LC-MALDI-TOF/TOF MS, liquid chromatography-matrix-assisted laser desorption/ionization time-of-flight tandem mass spectrometry.

The SVM analyses revealed that the peptidomic patterns at 0 min, 24, 48, and 168 h were clearly separated, having a cross-validation accuracy of no less than 83.8%. The top 2 peaks that most efficiently discriminated among the time points at 0 min, 24, 48, and 168 h were those at 2378 and 9080 *m/z* (P-values <0.00001 and <0.00001, respectively, (Fig 4)). The peak at 2378 *m/z* was up-regulated until 168 h, whereas the peak at 9080 *m/z* was down-regulated. These observations were fully reproduced in the same experiments performed on consecutive 3 days (S5 Table). LC-MALDI-TOF/TOF MS analysis identified the peak at 2378 *m/z* as a fragment derived from complement C4b (position:1429–1449).

## Discussion

In this study, we provided a biomarker strategy based on peptide extraction using C8-coated magnetic beads and MALDI-TOF/TOF MS analysis for defining the peptide profiles in plasma during the postoperative course of PHx in a swine model. In order to construct stable peptide profile platform in this model, it is important to set a sampling point and method. After comparing the peptide profiles in PV and CV, or serum and plasma, it was confirmed that circulating peptide profiles could be influenced by the anatomical site from which samples were obtained as well as the clotting process (S6 Table). This provides a proof that the native plasma peptidome reflects proteolytic processes *in vivo*. Using plasma in CV, we successfully generated a plasma peptide profile that could be used for identification of the very acute (0 min), acute (24 h), and recovery phases (120 h) after surgery. To our knowledge, this is the first report indicating that endogenous plasma peptides after PHx can have a diagnostic value with phase-specific relevance. Several biomarker peaks were extracted that can potentially be used to reliably differentiate between each phase. Those phase-specific marker peptides were identified as fragments of histones in the very acute phase, fragments of HBA in the acute phase, and fragments of ALB in the recovery phase.

Immediately after PHx, nucleosome components such as linker histone (H1), and core histones (H2A, H2B, H3, and H4) were detected. Through in-depth data analysis, a highly selective epitope was detected from each histone proteins; the C-tail of H2A, the N- and C-tails of H2B, and the N-tail of H4 were detected. The abundance of these particular fragments can probably be explained by the fact that the N-tails of all core histones protrude outside the nucleosome, and the C-tails of H2A and H2B also protrude outside the nucleosomal core; thus, these exposed tail regions are susceptible to proteolytic cleavage [22]. Acetylated lysine residues were found in the N-tail fragments of H4 which, as noted above, are more readily accessible than the nucleosomal core regions and therefore are also susceptible to post-translational modifications. Emerging studies indicate that besides having nuclear functions, histones can also be released into the extracellular space by both damaged and activated cells, exhibiting significant toxic or pro-inflammatory activity both *in vivo* and *in vitro* [23]. In a mice model of the hepatic ischemia/reperfusion injury, the extracellular histones released from liver parenchymal cells have been shown to exacerbate liver injuries through Toll-like receptor-9 activation [24]. The extracellular release of histone fragments might be an immediate/early signal for triggering liver regeneration after PHx.

The abundance of HBA fragments also changed in the acute phase. The abundance of the N-terminal part fragment (3627 *m/z*) decreased until 24 and 48 h after PHx. However, that of the C-terminal part fragments (3538 and 3852 *m/z*) increased until 6 h (S2 Table). HB is important not only as a component of red blood cells, but also as an extracellular substrate in plasma of various diseases [25]. In obese patients, serum HBA and HB subunit β were reported to be up-regulated according to the severity of liver disease from non-alcoholic fatty liver diseases to steatohepatitis [26]. At the transcriptomic level in liver, HBA has been reported to be repressed at 48–72 h after 70% PHx in rats [27]. In non-alcoholic steatohepatitis, high expression levels of HBA and HB subunit β have been observed in liver biopsy specimens due to oxidative stress [28]. We consider that HB may be a prominent source of bioactive peptides playing an important role in maintaining homeostasis after PHx and in liver diseases.

During the recovery phase, ALB fragments were identified as the phase-determinant peptides and were mostly derived from the N-terminal part of ALB. (position: 25-X amino acids) Applied to the structure of human ALB, these fragments are considered to belong to the subdomain IA [29, 30]. This subdomain is structurally and functionally characterized as having a region of extended polypeptide chain and binding sites for metal ions such as copper and zinc. Among them, the short N-terminal fragments (3000, 3028 and 3042 *m/z*) were down-regulated, and the long N-terminal fragment (8926 *m/z*) was up-regulated. The short N-terminal fragments are estimated to reflect the turnover of ALB by the ubiquitin proteasome system and were continuously down-regulated until 168 h. The abundance of the long N-terminal fragment was increased, although its origin was unclear. At the transcriptomic level, ALB expression in liver was decreased until 36–48 h and gradually increased thereafter [31]. In clinical practice, the serum ALB level is a component of the Child–Pugh score, which is often used as a diagnostic tool of liver function [32]. Though the overall ALB concentration is important in this scoring system, it is reported to be easily influenced by the measurement method [33]. Many biomarker discovery efforts have searched for variations in the total abundance of particular proteins. In our data, different epitopes in the protein were specifically and significantly associated with phases of the postoperative course after PHx. Especially, the long N-terminal fragment (8926 *m/z*) was also seen in the recovery phase after carbon tetrachloride-induced liver injury in pigs (data not shown). Instead of the whole protein, the N-terminal fragments of ALB might have the potential to be used as biomarkers of liver recovery in various liver diseases.

However, there might be some arguments against sample preparation using only magnetic beads in light of the sensitivity of this method for detecting plasma native peptides. Because human plasma proteins range in abundance by 10–12 orders of magnitude, it is quite difficult to detect all of the proteins at the same time [13]. The detection of low-abundance proteins needs extensive sample preparation such as the immunodepletion of high-abundance proteins including albumin and immunoglobulin, and sample fractionation using 2-dimensional electrophoresis or chromatography [34]. In this study, we focused on moderate- to high-abundance plasma proteins and their fragments, resulting in novel findings regarding their phase-specific relevance and capacity to be used as biomarkers of liver regeneration after PHx. A growing body of evidence suggests that altered protease activities are involved in many diseases and could potentially generate a large number of peptides and small proteins [35]. They are thought to be released from their parent proteins by endoproteolytic cleavage, followed by the variable trimming of released peptides by aminopeptidases and carboxypeptidases [36]. While some researchers have dismissed the peptidome as biological trash, we can add an important diagnostic paradigm even by considering the fragments of major proteins, including histones, ALB, and HBA.

In this study, using a swine PHx model, we elucidated the phase-specificity of plasma peptide profiles and extracted peptide biomarkers of each phase after PHx. Because it is simple and needs no more than a few microliters of plasma specimens to analyze, the magnetic bead-based biomarker strategy is considered to be suitable for application in clinical trials. Our goal is to establish diagnostic markers of liver regeneration following human liver surgery using this approach. Clinical applications using a small number of subjects revealed that a fragment of complement C4b in plasma was increased by about 3–5 fold at 168 h of liver regeneration after PHx. Complement activation is a well-studied phenomenon in terms of ischemia/reperfusion liver injuries. This pathological condition is often a result of the Pringle maneuver during liver resection, which is routinely performed for reduction of blood loss. In all the 4 cases in our study, major hepatectomies were performed using this maneuver. A previous study showed that the C4b fragment was deposited in the hepatocytes of ischemia/reperfusion injured liver after hepatectomy in humans [37]. Thus, our findings regarding C4b could be associated with ischemia/reperfusion injuries during liver surgery.

We have previously detected C4 fragments in a murine carbon tetrachloride-induced liver injury model and other groups have also reported the potential contribution of complement to regenerative process after PHx in mice [38, 39]. C4b has not been identified as a marker peptide in the pig experiments, and it could be in part because amino acid sequence of the swine C4b protein is not listed in the database. Approximately 80% of the pig proteome is still poorly annotated, and the existence of protein sequences is routinely inferred by sequence alignment with preexisting sequences [40].

It must be noted here that the study does not include data from sham-operated pigs. Considering the fact that sham operation is one way of extracting specific biomarkers in PHx model, certain limitations arising from the unavailability of such data in this study must be acknowledged. Instead, we carefully extracted phase-specific peptidomic profiles from 5 animals in a longitudinal time course. This is one of the advantages of using a pig model over a rodent model. The incorporation of proper sham-operated controls (0% PHx) and 30% or 90% PHx could further help validate the observations presented in this study, as well as enable extraction of more specific and sensitive biomarkers of liver regeneration, and therefore, must be considered in further studies. Furthermore, it would be interesting to compare these observations with those in other models such as carbon tetrachloride-induced liver injury model as we have done using mice [38]. The approach adopted in this study is considered to be applicable to the search for biomarkers of liver regeneration not only after PHx but also after preoperative portal vein embolization [41], and after associating liver partition and portal vein ligation for staged hepatectomy (ALPPS) surgery [42], and therefore, we cautiously plan to conduct clinical trials and elucidate biomarkers for clinical use in our subsequent work.

## Conclusions

We have proved that native plasma peptidomic profiling using MALDI-TOF MS can clearly delineate the postoperative course after PHx in a biphasic manner by distinguishing the acute phase and recovery phase. These phases could be clearly identified by the presence of HBA and ALB fragments, respectively. Further in-depth analysis using LC-MALDI-TOF/TOF MS revealed the presence of phase-specific peptides, such as histone fragments in the very acute phase. Finally, in a study of human plasma samples from hepatectomy cases, adopting the same strategy, we have extracted phase-specific peptidomic patterns by a machine learning method and further detected a fragment of complement C4b as a peptide biomarker of liver regeneration. We anticipate that the data presented in this study may help to understand the biological process of liver regeneration and improve the outcome of patients after PHx.

## Supporting Information

S1 Fig Pseudogel and stack view of the spectra obtained from plasma of 3 pigs. (Page 2) 0 min *vs*. 24 h, 24 h *vs*. 48 h, (Page 3) 48 h *vs*. 72 h, 72 h *vs*. 96 h, (Page 4) 96 h *vs*. 120 h, 120 h *vs*. 168 h, (Page 5) Pre *vs*. 0 min, Pre *vs*. 1 h, (Page 6) Pre *vs*. 3 h, Pre *vs*. 6 h, (Page 7) Pre *vs*. 24 h, Pre *vs*. 48 h, (Page 8) Pre *vs*. 72 h, Pre *vs*. 96 h, (Page 9) Pre *vs*. 120 h, Pre *vs*. 144 h, (Page 10) Pre *vs*. 168 h, 0 min *vs*. 1 h, (Page 11) 0 min *vs*. 3 h, 0 min *vs*. 6 h, (Page 12) 0 min *vs*. 48 h, 0 min *vs*. 72 h, (Page 13) 0 min *vs*. 96 h, 0 min *vs*. 120 h, (Page 14) 0 min *vs*. 144 h, 0 min *vs*. 168 h, (Page 15) 1 h *vs*. 3 h, 1 h *vs*. 6 h, (Page 16) 1 h *vs*. 24 h, 1 h *vs*. 48 h, (Page 17) 1 h *vs*. 72 h, 1 h *vs*. 96 h, (Page 18) 1 h *vs*. 120 h, 1 h *vs*. 144 h, (Page 19) 1 h *vs*. 168 h, 3 h *vs*. 6 h, (Page 20) 3 h *vs*. 24 h, 3 h *vs*. 48 h, (Page 21) 3 h *vs*. 72 h, 3 h *vs*. 96 h, (Page 22) 3 h *vs*. 120 h, 3 h *vs*. 144 h, (Page 23) 3 h *vs*. 168 h, 6 h *vs*. 24 h, (Page 24) 6 h *vs*. 48 h, 6 h *vs*.72 h, (Page 25) 6 h *vs*. 96 h, 6 h *vs*. 120 h, (Page 26) 6 h *vs*. 144 h, 6 h *vs*. 168 h, (Page 27) 24 h *vs*. 72 h, 24 h *vs*. 96 h, (Page 28) 24 h *vs*. 120 h, 24 h *vs*. 144 h, (Page 29) 24 h *vs*. 168 h, 48 h *vs*. 96 h, (Page 30) 48 h *vs*. 120 h, 48 h *vs*. 144 h, (Page 31) 48 h *vs*. 168 h, 72 h *vs*. 120 h, (Page 32) 72 h *vs*. 144 h, 72 h *vs*. 168 h, (Page 33) 96 h *vs*. 144 h, 96 h *vs*. 168 h, (Page 34) 120 h *vs*. 144 h, 144 h *vs*. 168 h, (Page 35) all timings (pseudogel view), and (Page 36) all timings (stack view).
(PDF)

S2 Fig Representative MS/MS spectra of swine plasma peptides. (Page 2~9) serum albumin, (Page 10~14) hemoglobin subunit α, (Page 15~17) basic proline-rich protein, (Page 18~19) histone H1.2, (Page 20) histone H2A1, (Page 21~22) histone H2B1, (Page 23~24) histone H3.1, (Page 25) histone H4, (Page 26~27) non-histone chromosomal protein HMG-17, (Page 28) serotransferrin, (Page 29) apolipoprotein C-III, (Page 30) ryanodine receptor I, and (Page 31) vasodilator-stimulated phosphoprotein.
(PPTX)

S1 Table Characteristics of patients analyzed in this study. Four patients who underwent major hepatectomy without pathological liver fibrosis (F0 or F1) [17], and had an uneventful postoperative course without PHLF, were included. *^1^The primary tumor originated in stomach. ^*2^The definition of PHLF is an increase in the international normalized ratio of prothrombin time and concomitant hyperbilirubinemia (according to the normal limits of the local laboratory) on or after postoperative day 5 [4]. PHLF, posthepatectomy liver failure; nonB, nonC HCC, hepatocellular carcinoma without hepatitis B or C viral infection.
(DOCX)

S2 Table Detailed results of comparative analyses between every 2 timings after PHx in pigs. The top 2 peaks enabling discrimination between every 2 timings were shown in the table. Ave*N* indicated peak area/intensity average of class *N* (1 or 2). StdDev*N* indicated standard deviation of the peak area/intensity average of class *N*. PHx, partial hepatectomy.
(XLSX)

S3 Table Peak area/intensity of marker peptides in swine plasma after PHx. (Page 1) first cohort and (Page 2) second cohort analyses. Ave*N* indicated peak area/intensity average of class *N* (1 to 6). StdDev*N* indicated standard deviation of the peak area/intensity average of class *N*. Class 1, 2, 3, 4, 5, and 6 represented pre, 0 min, 24, 48, 120, and 168 h, respectively. PHx, partial hepatectomy.
(XLSX)

S4 Table The endogenous swine plasma peptides after PHx identified by LC-MALDI-TOF/TOF MS. (Page 1) 0 min, (Page 2) 24 h, (Page 3) 48 h, and (Page 4) 168 h after PHx. LC-MALDI-TOF/TOF MS, liquid chromatography-matrix-assisted laser desorption/ionization time-of-flight tandem mass spectrometry; PHx, partial hepatectomy.
(XLSX)

S5 Table Three independent datasets for human plasma analyses. The peaks at 2378 and 9080 *m/z* clearly discriminated 0 min, 24, 48, and 168 h with P-values of no more than 0.0001 in every 3 experiments. Ave*N* indicated peak area/intensity average of class *N* (1 to 4). StdDev*N* indicated standard deviation of the peak area/intensity average of class *N*. Class 1, 2, 3, and 4 represented 0 min, 24, 48, and 168 h, respectively. The *x* and *y* axes of the 2-dimensional distribution plots represented 2378 and 9080 *m/z*, respectively.
(XLSX)

S6 Table The peak list that had a significant difference* in intensity regarding sampling points and methods. (A) More peptide fragments were detected in PV than in CV. This result might reflect PV containing more humoral factors than CV that are capable of stimulating DNA synthesis after PHx. (B) More peptide fragments were observed in serum than in plasma. The lower stability of peptide fragments in serum is considered to result from ex vivo multiprotease activities due to clotting. The SVM analyses discriminated PV and CV, and serum and plasma with a cross-validation accuracy of more than 88 and 93% at all timings, respectively. In the present study, the plasma samples were analyzed to detect endogenous peptides. Considering the difficulty in repeated sampling from PV, which otherwise might be more informative, the CV specimens were used for further studies. *Differences with AUC calculated by ROC curve analysis > 0.8 were considered to be significant. Peaks were described with *m/z* values. Five and 4 pigs were used for analyses of (A) and (B), respectively. PV, portal vein; CV, central vein; AUC, area under the curve; ROC, receiver operating characteristics; *m/z*, mass to charge ratio.
(DOCX)
