# Supplementary material for: Chronological Profiling of Plasma Native Peptides after Hepatectomy in Pigs: Toward the Discovery of Human Biomarkers for Liver Regeneration
Source: PLoS One. 2017 Jan 6;12(1):e0167647. doi: 10.1371/journal.pone.0167647 (PMC5218562; doi:10.1371/journal.pone.0167647)
Supplement: S1 Fig — (Page 2) 0 min vs. 24 h, 24 h vs. 48 h, (Page 3) 48 h vs. 72 h, 72 h vs. 96 h, (Page 4) 96 h vs. 120 h, 120 h vs. 168 h, (Page 5) Pre vs. 0 min, Pre vs. 1 h, (Page 6) Pre vs. 3 h, Pre vs. 6 h, (Page 7) Pre vs. 24 h, Pre vs. 48 h, (Page 8) Pre vs. 72 h, Pre vs. 96 h, (Page 9) Pre vs. 120 h, Pre vs. 144 h, (Page 10) Pre vs. 168 h, 0 min vs. 1 h, (Page 11) 0 min vs. 3 h, 0 min vs. 6 h, (Page 12) 0 min vs. 48 h, 0 min vs. 72 h, (Page 13) 0 min vs. 96 h, 0 min vs. 120 h, (Page 14) 0 min vs. 144 h, 0 min vs. 168 h, (Page 15) 1 h vs. 3 h, 1 h vs. 6 h, (Page 16) 1 h vs. 24 h, 1 h vs. 48 h, (Page 17) 1 h vs. 72 h, 1 h vs. 96 h, (Page 18) 1 h vs. 120 h, 1 h vs. 144 h, (Page 19) 1 h vs. 168 h, 3 h vs. 6 h, (Page 20) 3 h vs. 24 h, 3 h vs. 48 h, (Page 21) 3 h vs. 72 h, 3 h vs. 96 h, (Page 22) 3 h vs. 120 h, 3 h vs. 144 h, (Page 23) 3 h vs. 168 h, 6 h vs. 24 h, (Page 24) 6 h vs. 48 h, 6 h vs.72 h, (Page 25) 6 h vs. 96 h, 6 h vs. 120 h, (Page 26) 6 h vs. 144 h, 6 h vs. 168 h, (Page 27) 24 h vs. 72 h, 24 h vs. 96 h, (Page 28) 24 h vs. 120 h, 24 h vs. 144 h, (Page 29) 24 h vs. 168 h, 48 h vs. 96 h, (Page 30) 48 h vs. 120 h, 48 h vs. 144 h, (Page 31) 48 h vs. 168 h, 72 h vs. 120 h, (Page 32) 72 h vs. 144 h, 72 h vs. 168 h, (Page 33) 96 h vs. 144 h, 96 h vs. 168 h, (Page 34) 120 h vs. 144 h, 144 h vs. 168 h, (Page 35) all timings (pseudogel view), and (Page 36) all timings (stack view). (PDF) [file pone.0167647.s001.pdf]

S1 Fig.

Pseudogel and stack view of the spectra obtained from plasma of 3 pigs.

(page 2) **0 min *vs.* 24 h, 24 h *vs.* 48 h,** (page 3) **48 h *vs.* 72 h, 72 h *vs.* 96 h,** (page 4) **96 h *vs.* 120 h, 120 h *vs.* 168 h,**

(page 5) **Pre *vs.* 0 min, Pre *vs.* 1 h,** (page 6) **Pre *vs.* 3 h, Pre *vs.* 6 h,** (page 7) **Pre *vs.* 24 h, Pre *vs.* 48 h,**

(page 8) **Pre *vs.* 72 h, Pre *vs.* 96 h,** (page 9) **Pre *vs.* 120 h, Pre *vs.* 144 h,** (page 10) **Pre *vs.* 168 h, 0 min *vs.* 1 h,**

(page 11) **0 min *vs.* 3 h, 0 min *vs.* 6 h,** (page 12) **0 min *vs.* 48 h, 0 min *vs.* 72 h,** (page 13) **0 min *vs.* 96 h, 0 min *vs.* 120 h,**

(page 14) **0 min *vs.* 144 h, 0 min *vs.* 168 h,** (page 15) **1 h *vs.* 3 h, 1 h *vs.* 6 h,** (page 16) **1 h *vs.* 24 h, 1 h *vs.* 48 h,**

(page 17) **1 h *vs.* 72 h, 1 h *vs.* 96 h,** (page 18) **1 h *vs.* 120 h, 1 h *vs.* 144 h,** (page 19) **1 h *vs.* 168 h, 3 h *vs.* 6 h,**

(page 20) **3 h *vs.* 24 h, 3 h *vs.* 48 h,** (page 21) **3 h *vs.* 72 h, 3 h *vs.* 96 h,** (page 22) **3 h *vs.* 120 h, 3 h *vs.* 144 h,**

(page 23) **3 h *vs.* 168 h, 6 h *vs.* 24 h,** (page 24) **6 h *vs.* 48 h, 6 h *vs.* 72 h,** (page 25) **6 h *vs.* 96 h, 6 h *vs.* 120 h,**

(page 26) **6 h *vs.* 144 h, 6 h *vs.* 168 h,** (page 27) **24 h *vs.* 72 h, 24 h *vs.* 96 h,** (page 28) **24 h *vs.* 120 h, 24 h *vs.* 144 h,**

(page 29) **24 h *vs.* 168 h, 48 h *vs.* 96 h,** (page 30) **48 h *vs.* 120 h, 48 h *vs.* 144 h,** (page 31) **48 h *vs.* 168 h, 72 h *vs.* 120 h,**

(page 32) **72 h *vs.* 144 h, 72 h *vs.* 168 h,** (page 33) **96 h *vs.* 144 h, 96 h *vs.* 168 h,** (page 34) **120 h *vs.* 144 h, 144 h *vs.* 168 h,**

(page 35) **all timings (pseudogel view),** and (page 36) **all timings (stack view).**

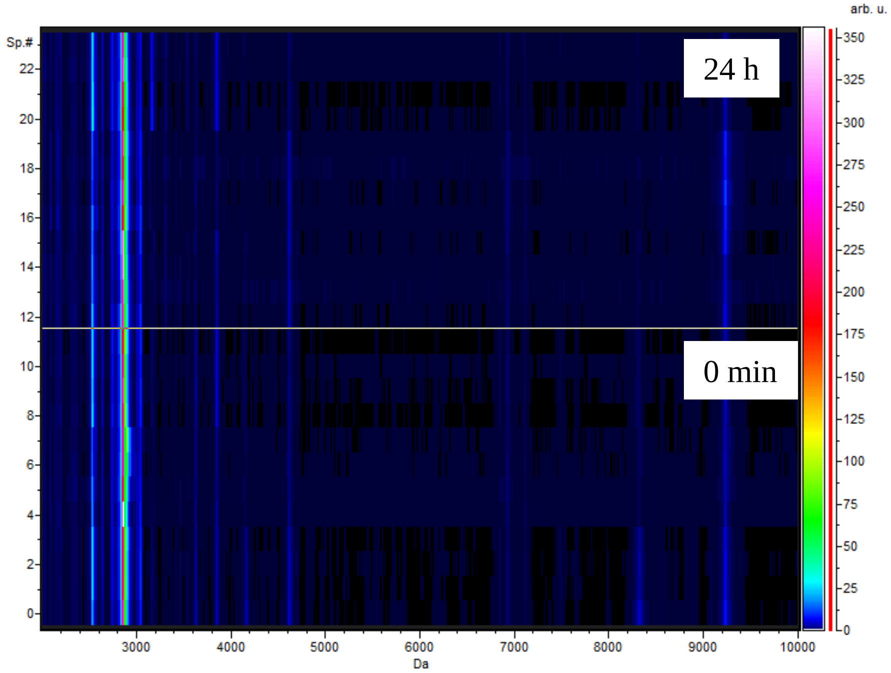
24 h
0 min
Sp.#
arb. u.
Da

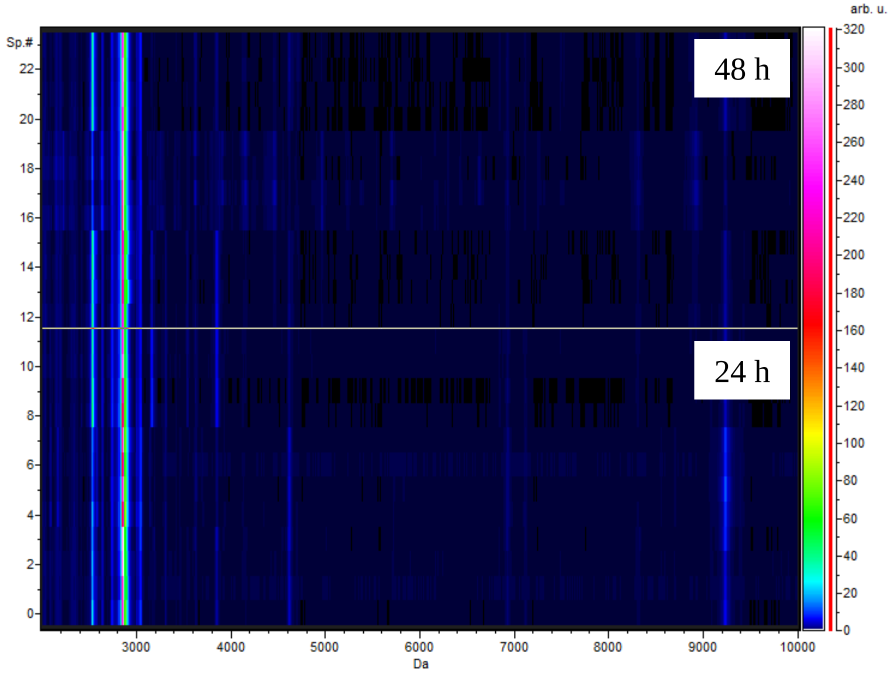
48 h
24 h
Sp.#
arb. u.
Da

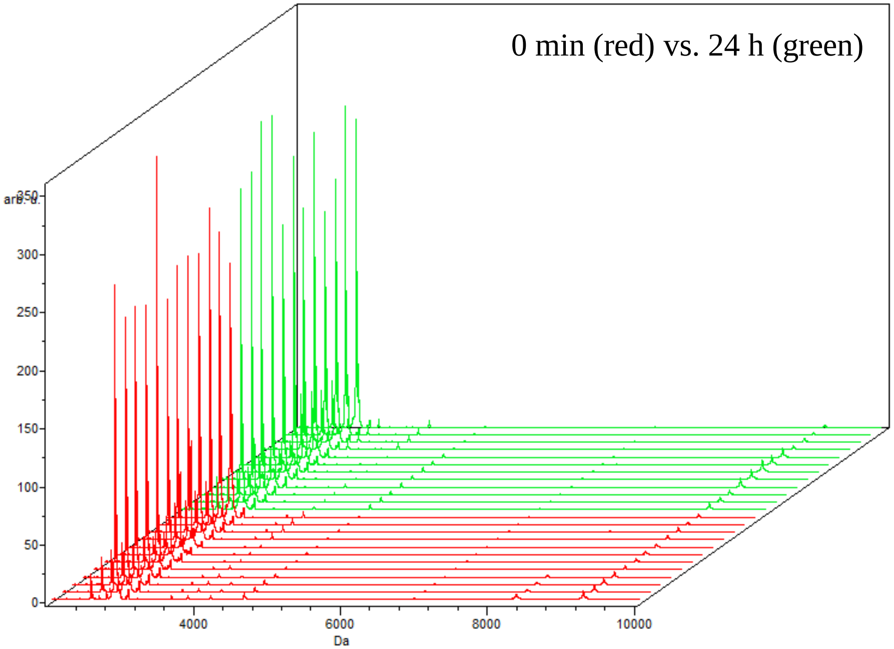
0 min (red) vs. 24 h (green)
arb. u.
Da

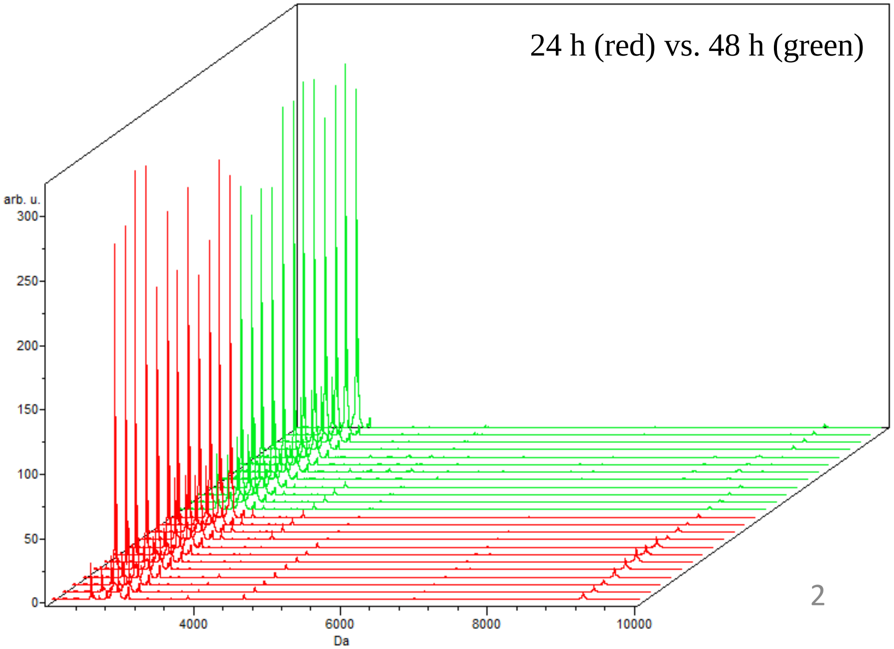
24 h (red) vs. 48 h (green)
arb. u.
Da

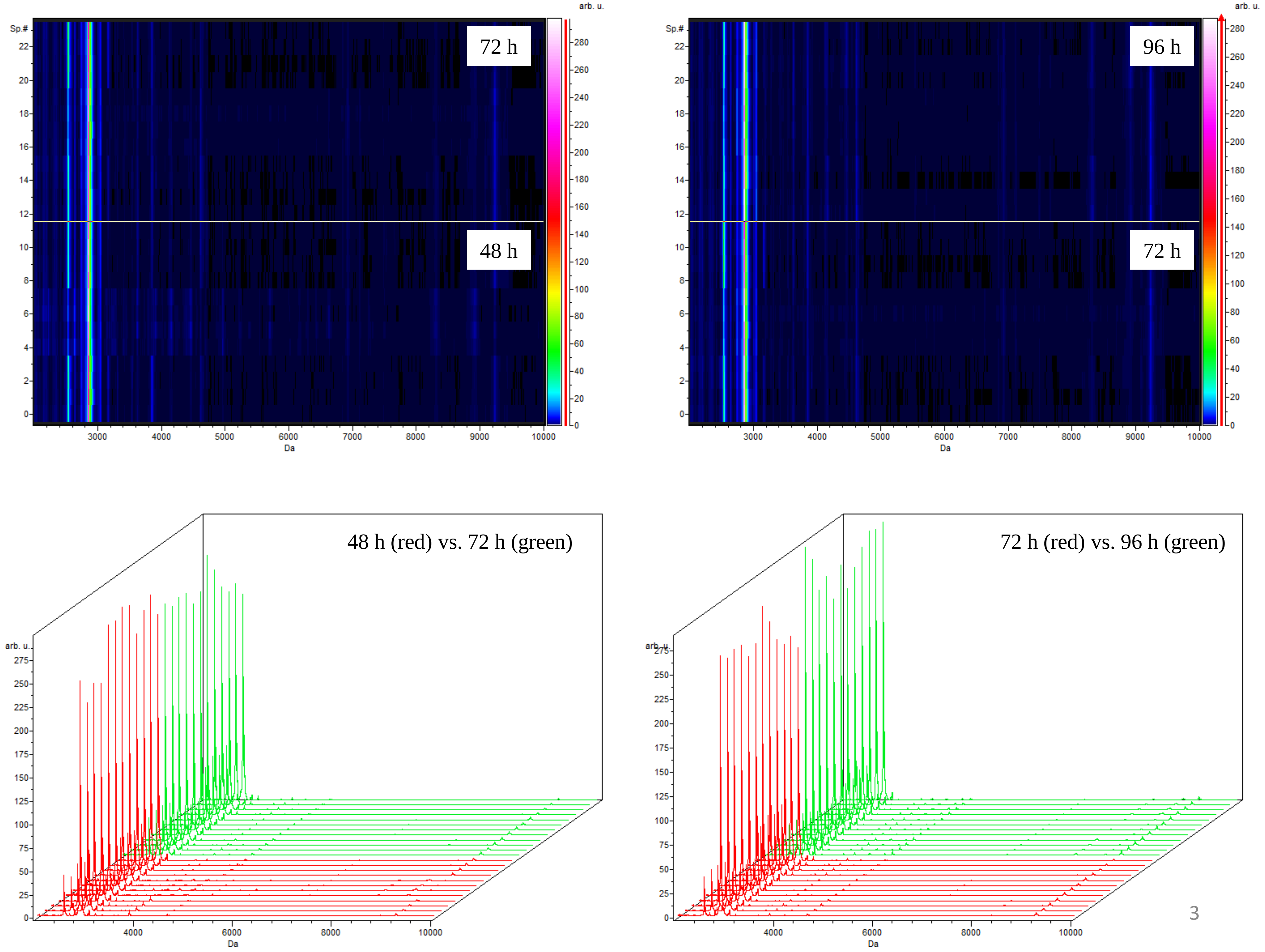
72 h
48 h
96 h
72 h
48 h (red) vs. 72 h (green)
72 h (red) vs. 96 h (green)
Sp.#
Da
arb. u.

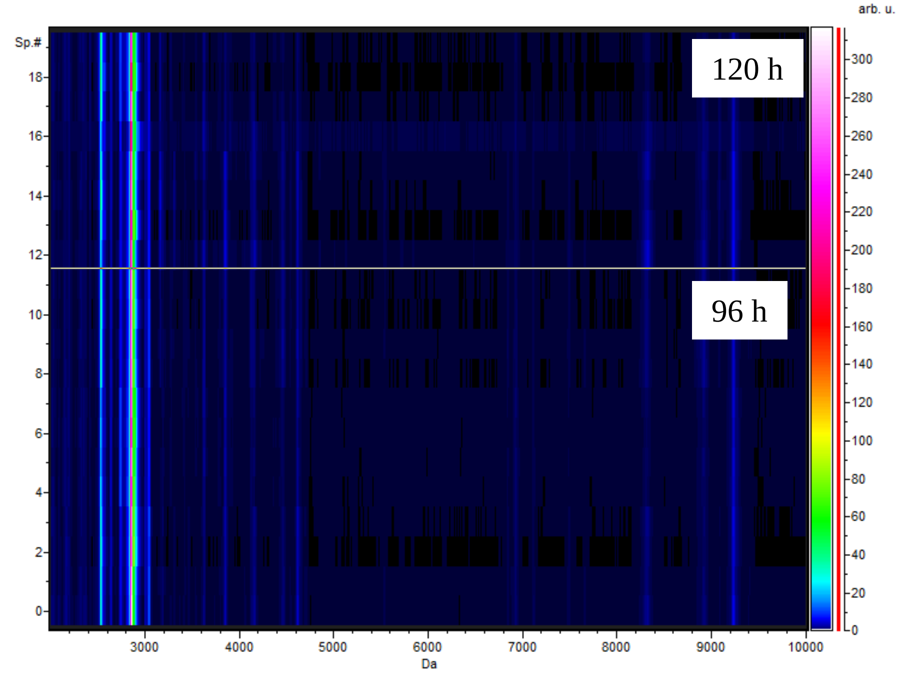
120 h
96 h
Sp.#
Da
arb. u.

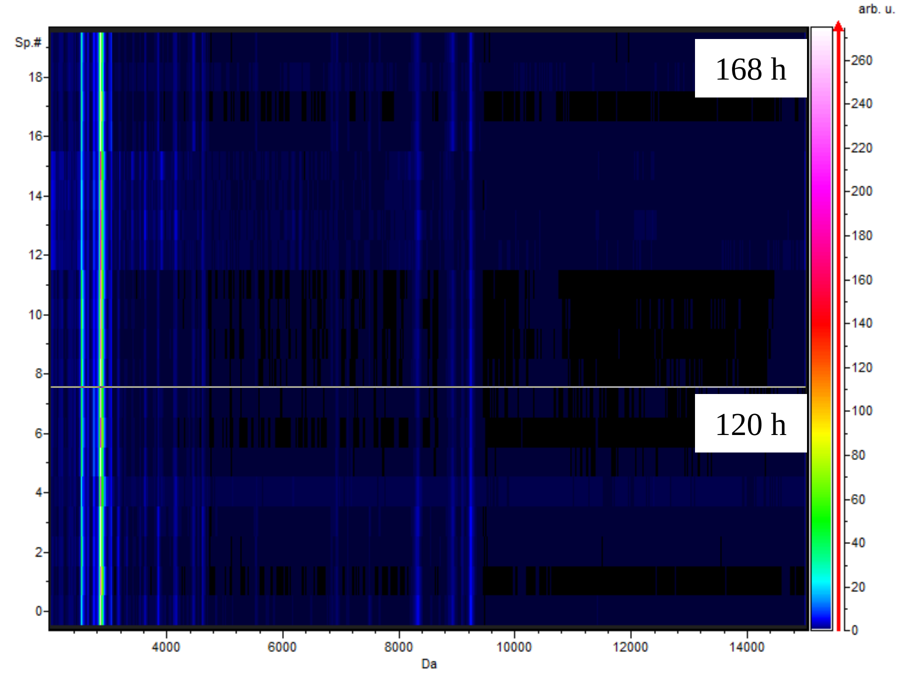
168 h
120 h
Sp.#
Da
arb. u.

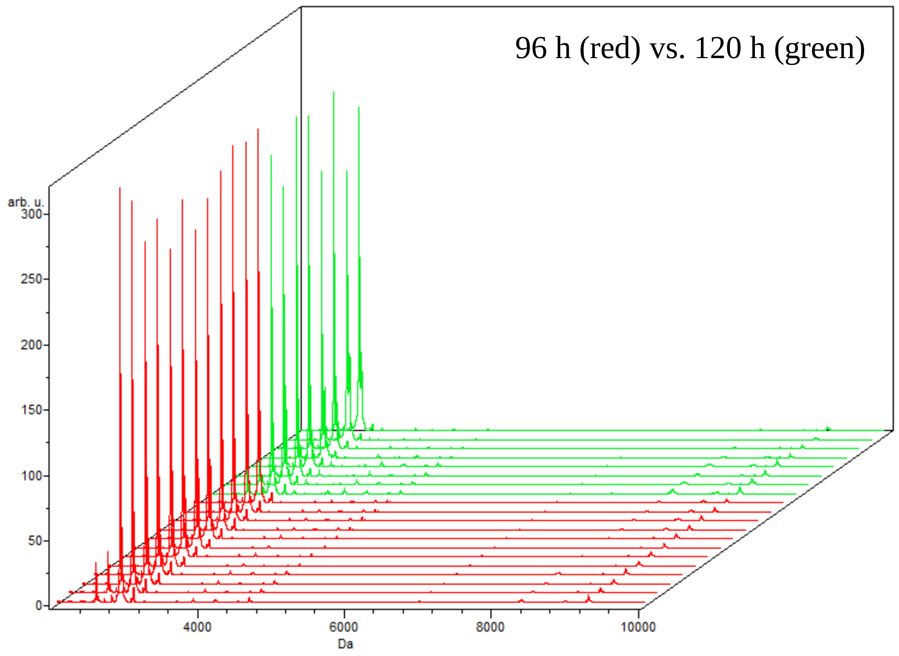
96 h (red) vs. 120 h (green)
arb. u.
Da

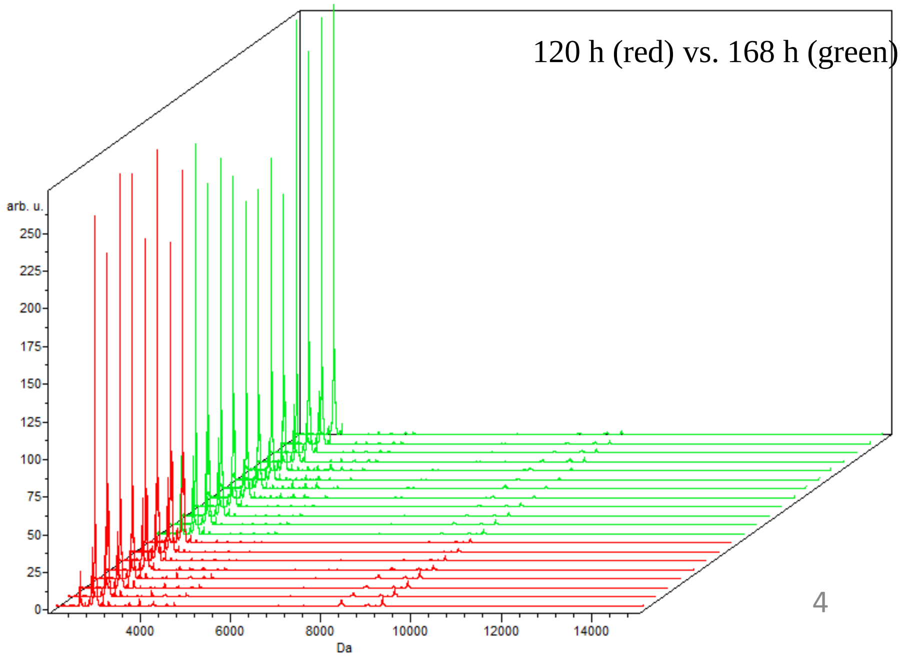
120 h (red) vs. 168 h (green)
arb. u.
Da

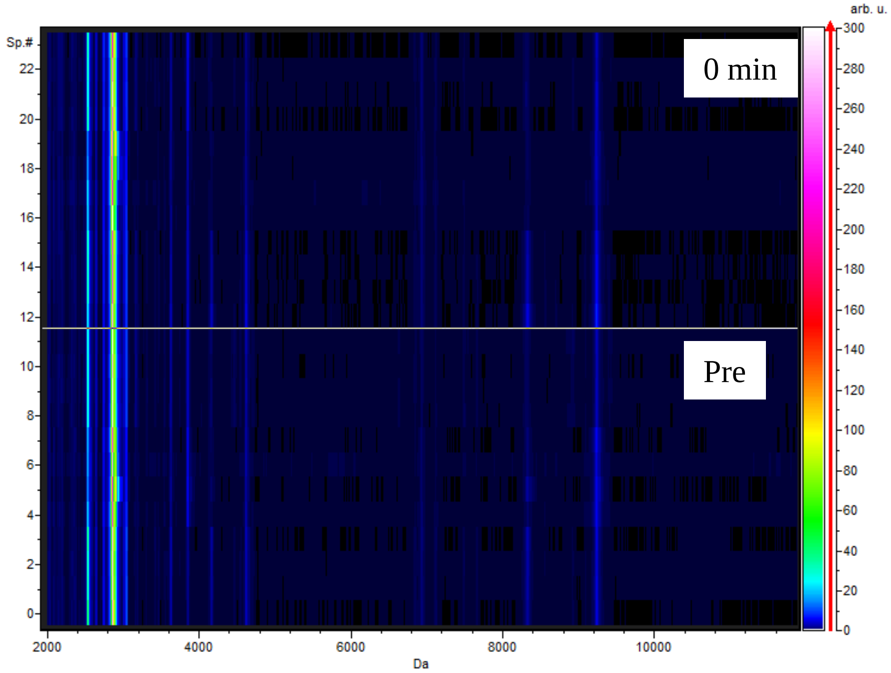
0 min
Pre
Sp.#
Da
arb. u.

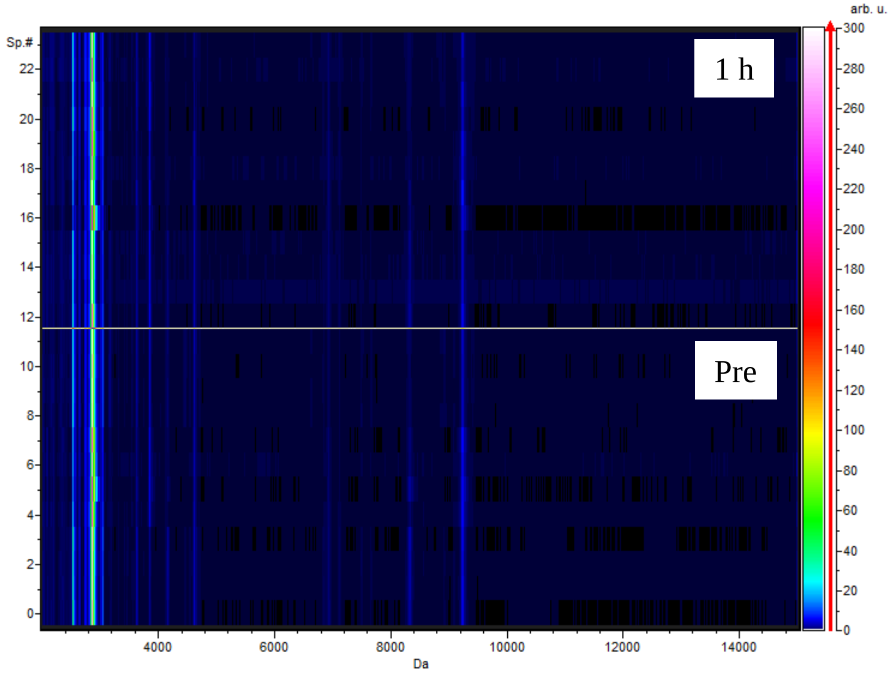
1 h
Pre
Sp.#
Da
arb. u.

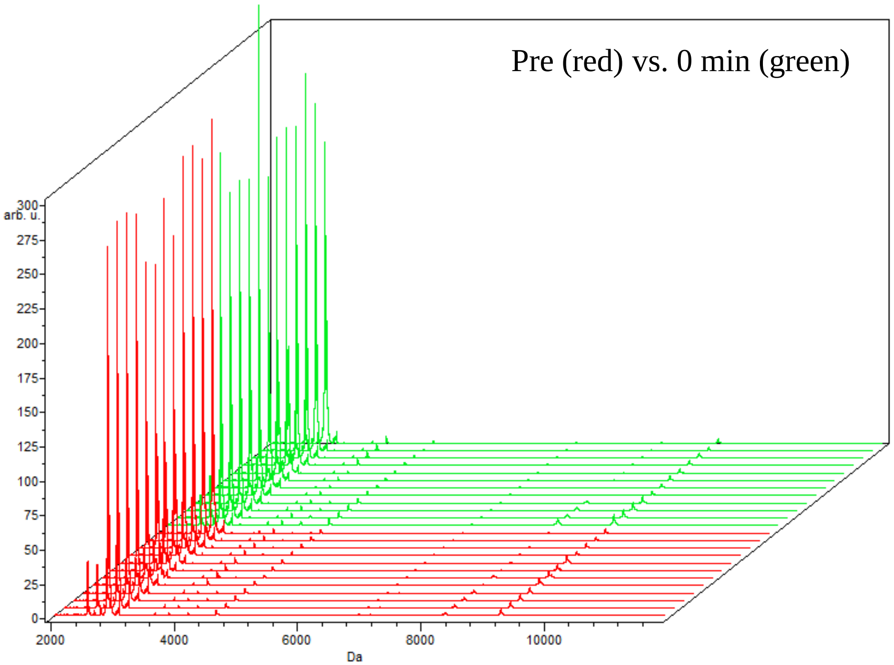
Pre (red) vs. 0 min (green)
arb. u.
Da

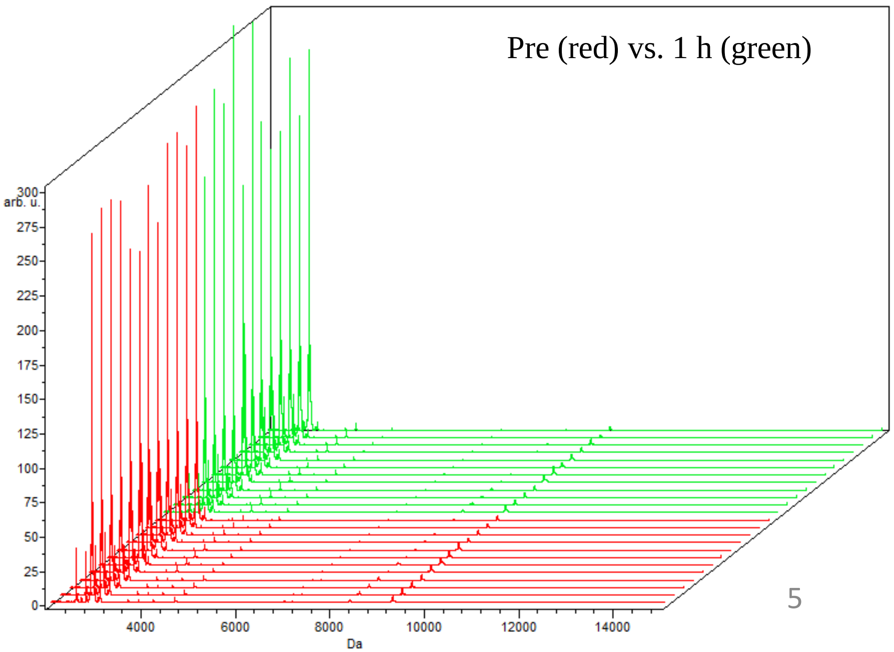
Pre (red) vs. 1 h (green)
arb. u.
Da

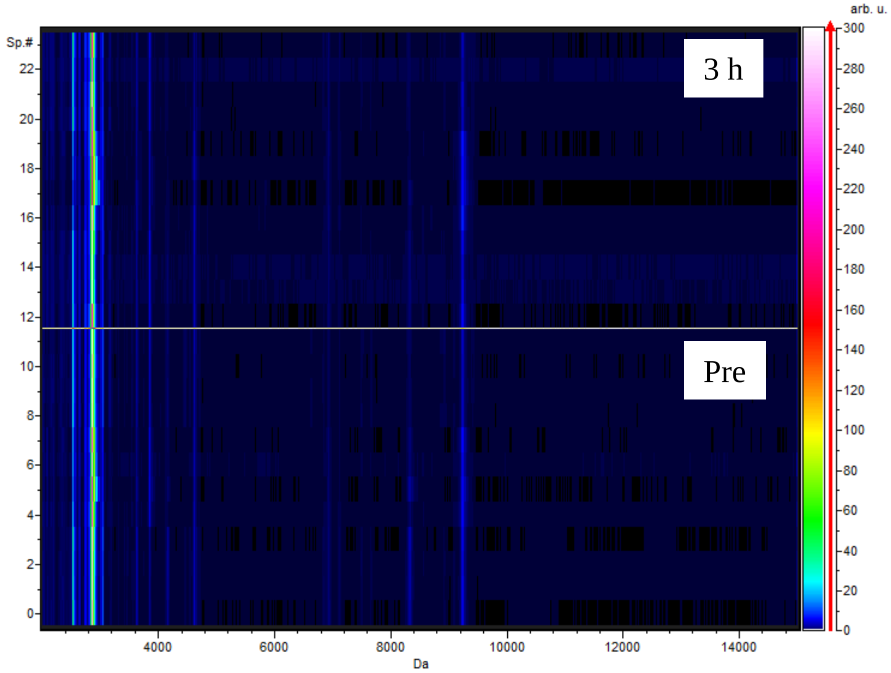
3 h
Pre
Sp.#
Da
arb. u.

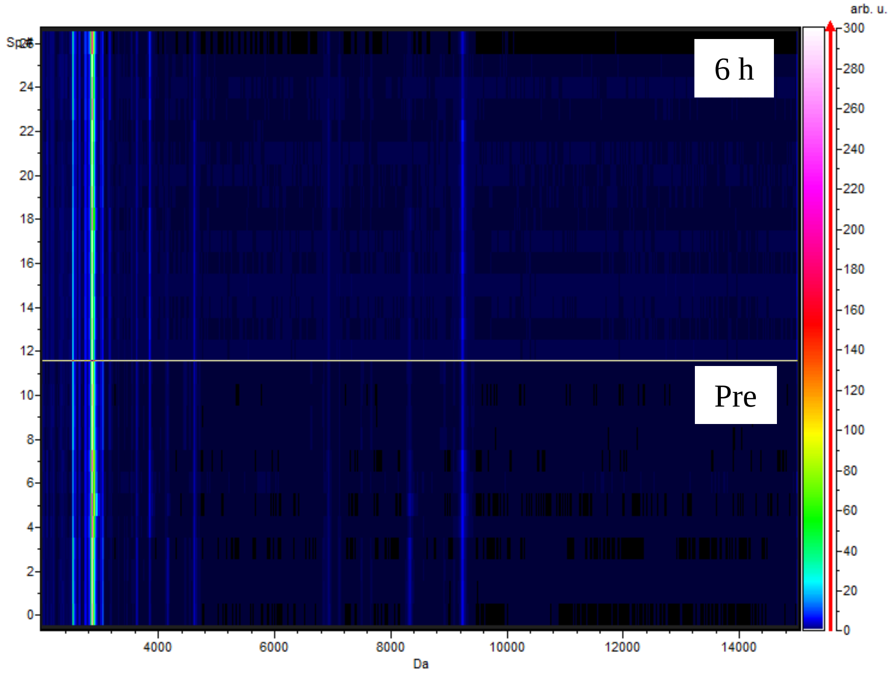
6 h
Pre
Sp.#
Da
arb. u.

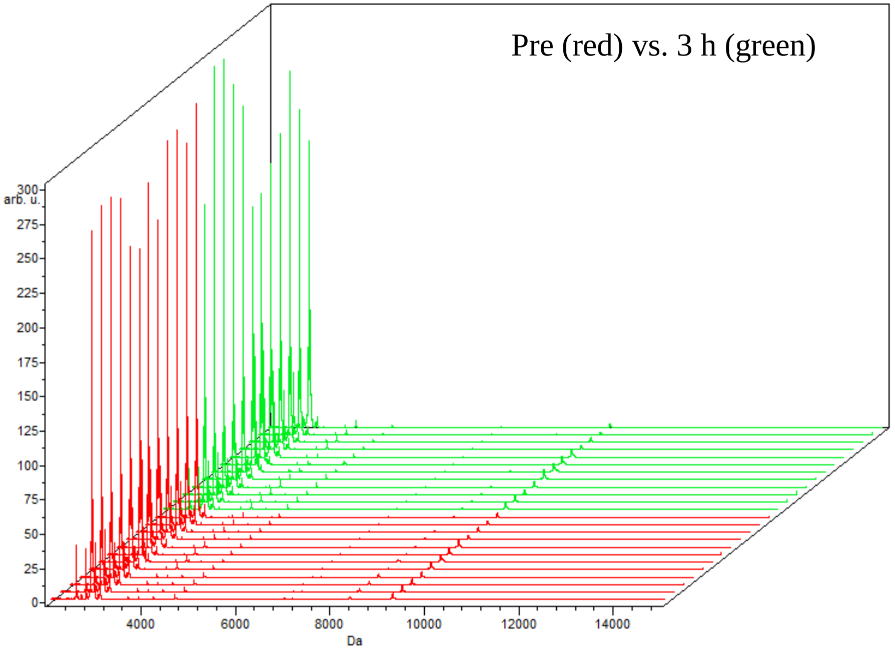
Pre (red) vs. 3 h (green)
arb. u.
Da

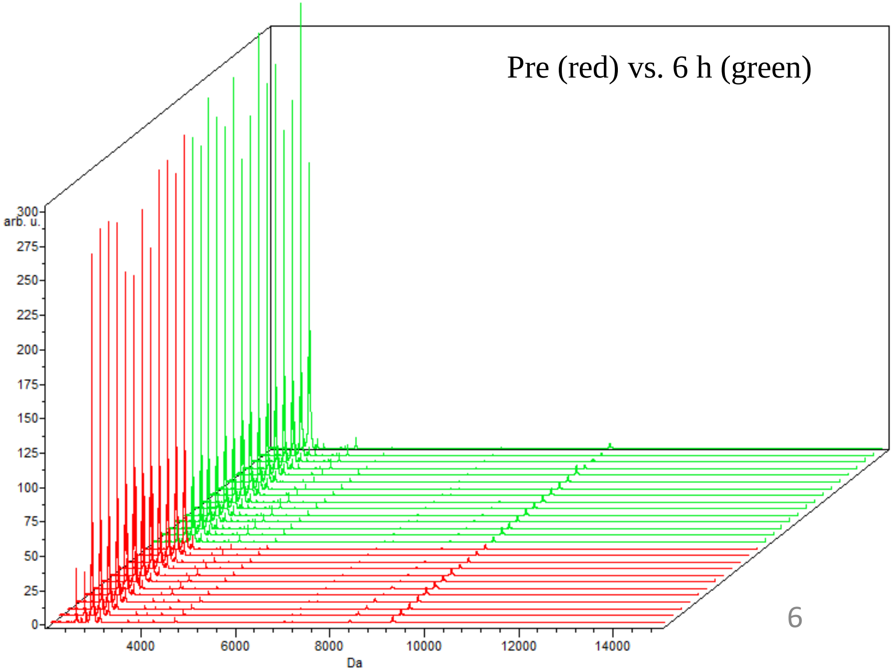
Pre (red) vs. 6 h (green)
arb. u.
Da

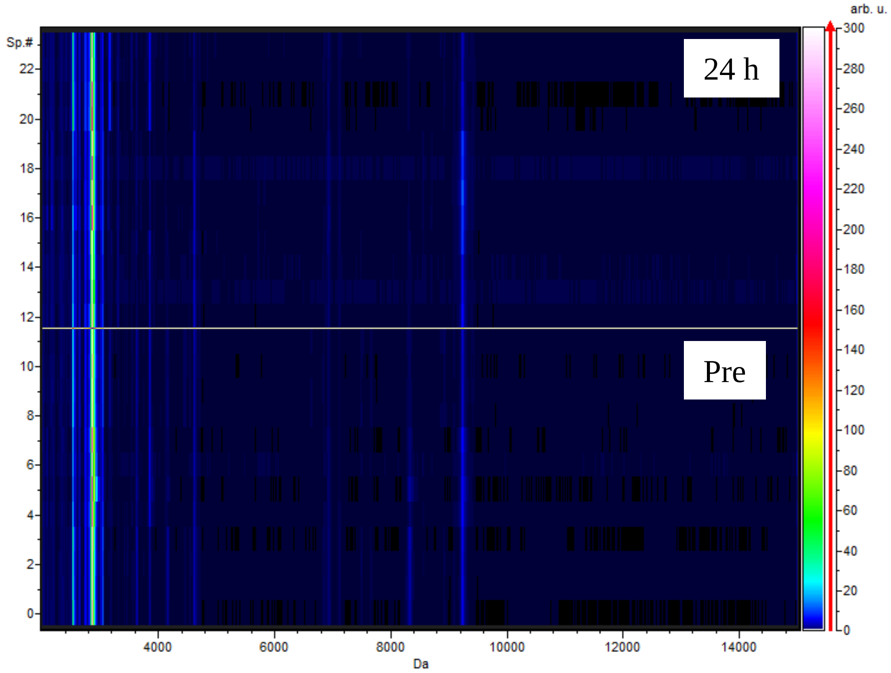
24 h
Pre
Sp.#
Da
arb. u.

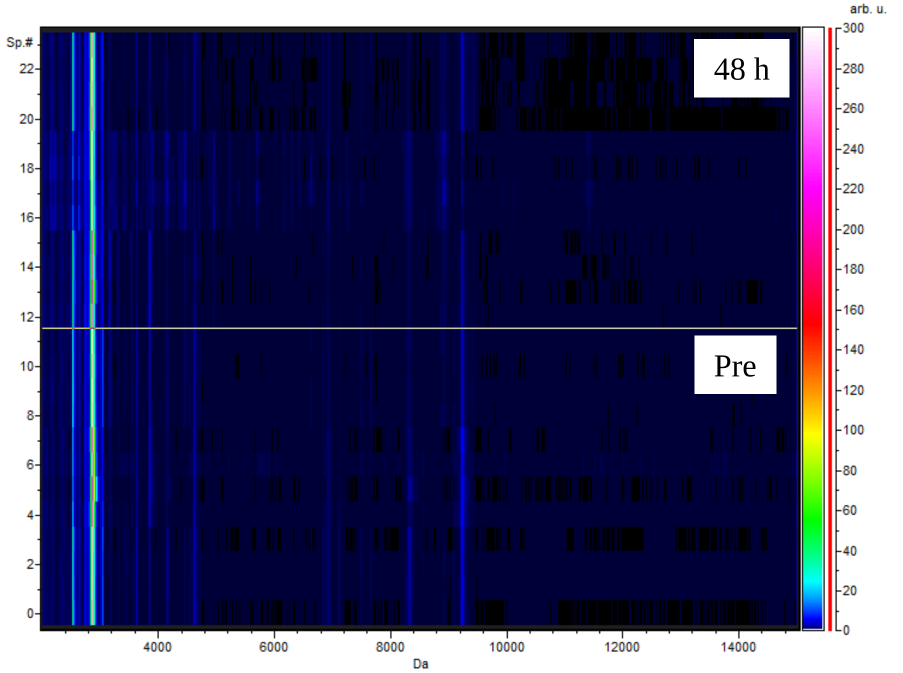
48 h
Pre
Sp.#
Da
arb. u.

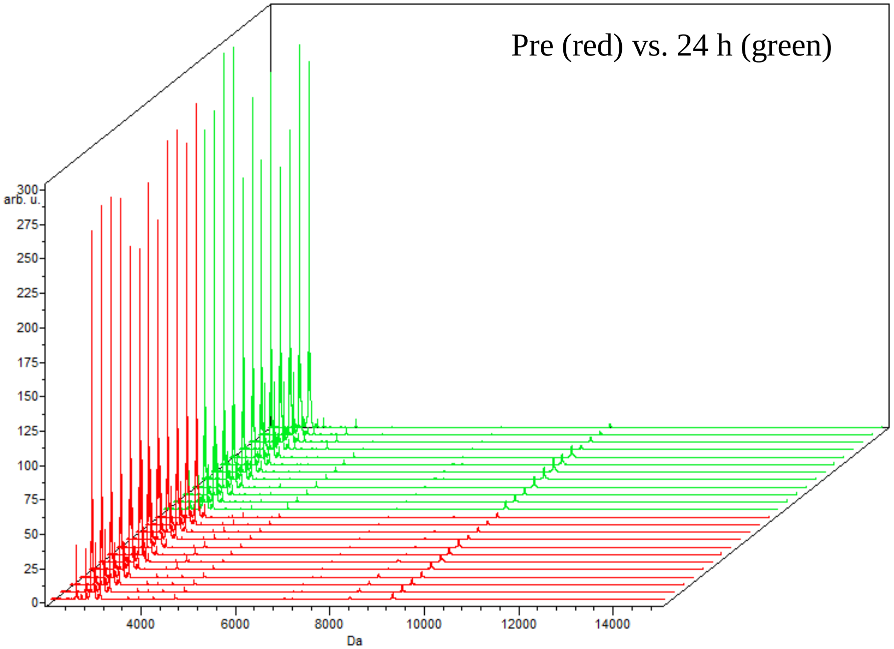
Pre (red) vs. 24 h (green)
arb. u.
Da

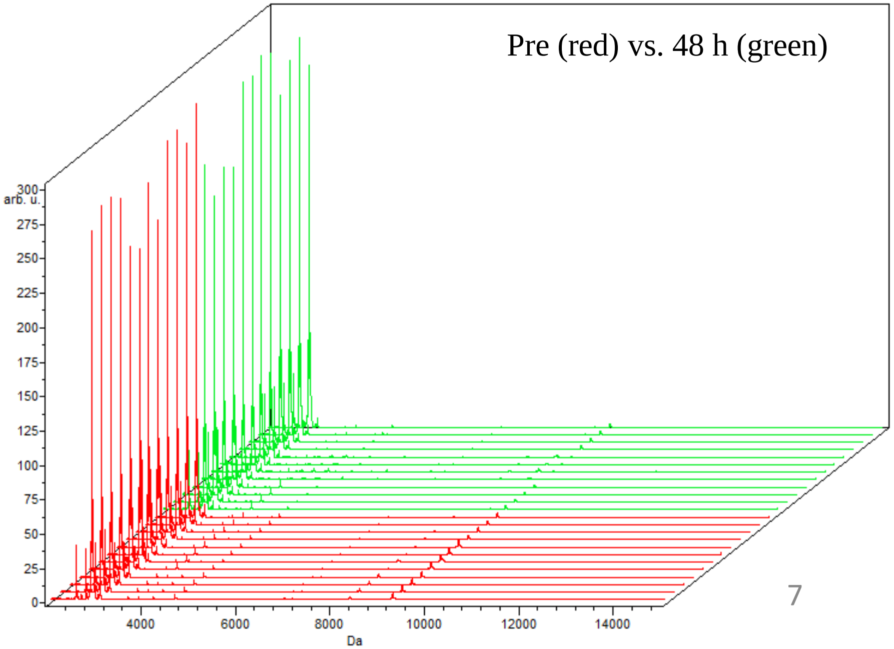
Pre (red) vs. 48 h (green)
arb. u.
Da

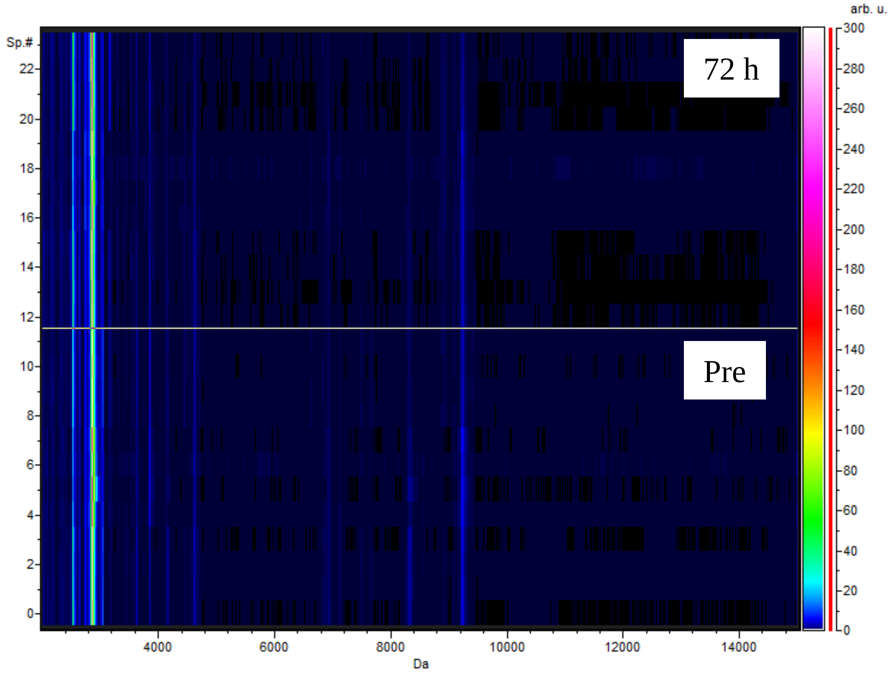
72 h
Pre
Sp.#
Da
arb. u.

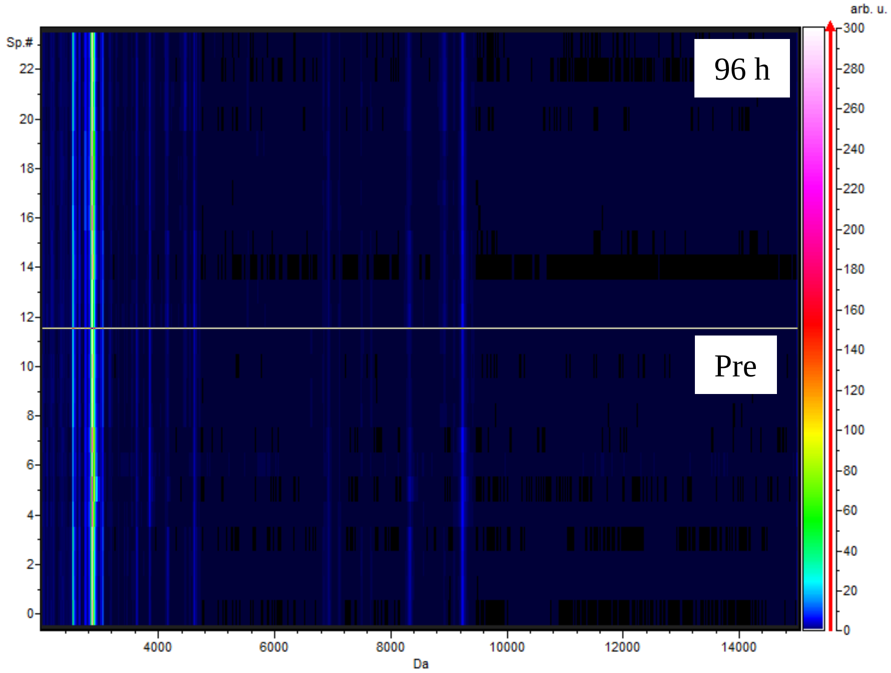
96 h
Pre
Sp.#
Da
arb. u.

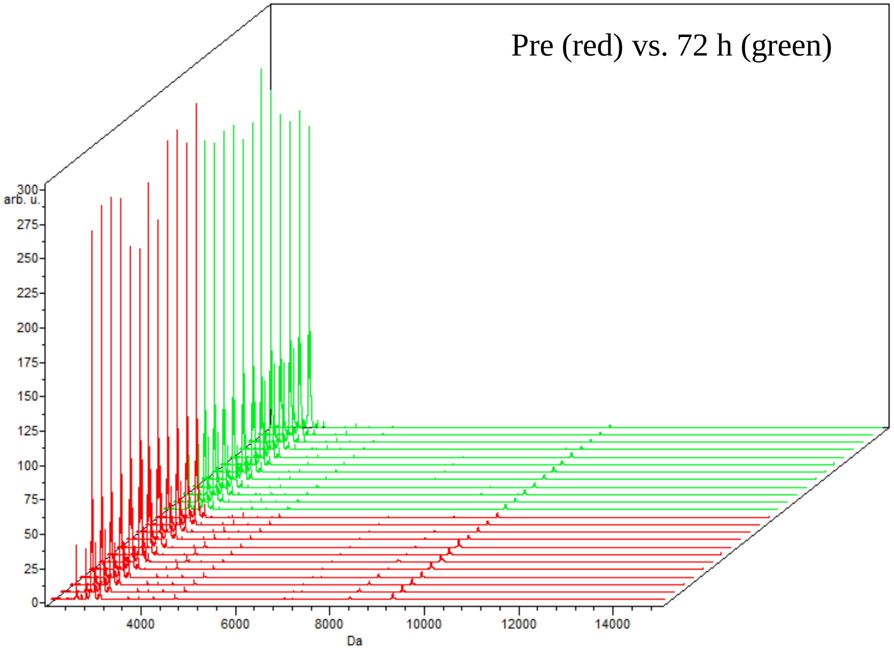
Pre (red) vs. 72 h (green)
arb. u.
Da

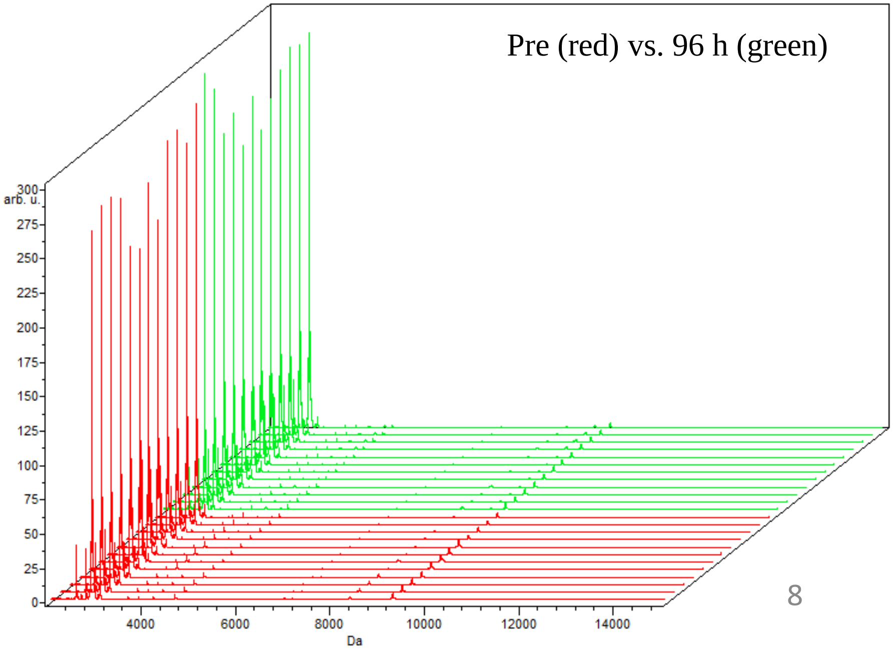
Pre (red) vs. 96 h (green)
arb. u.
Da

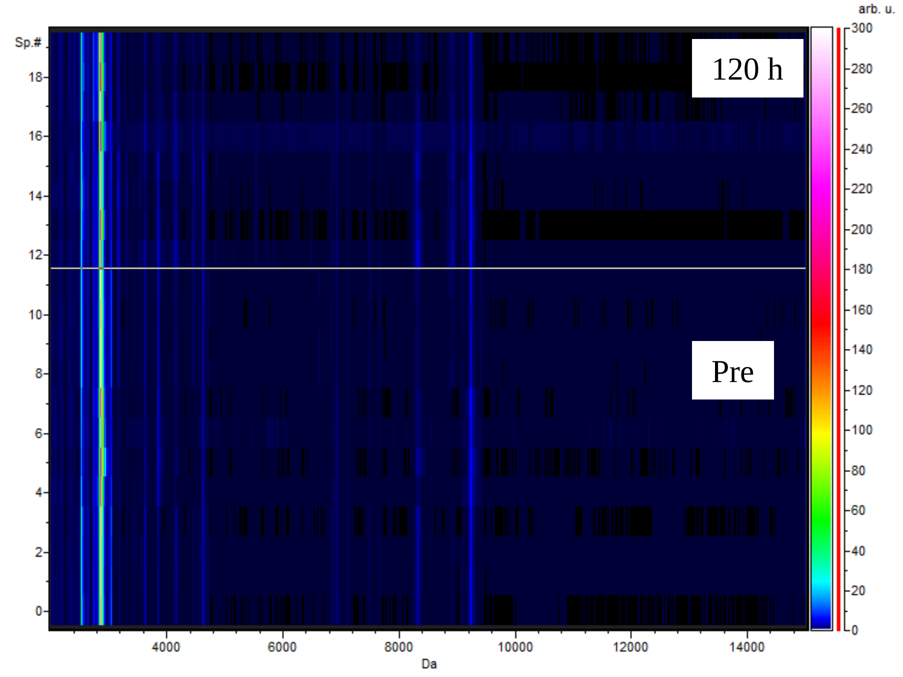
120 h
Pre
Sp.#
arb. u.
Da

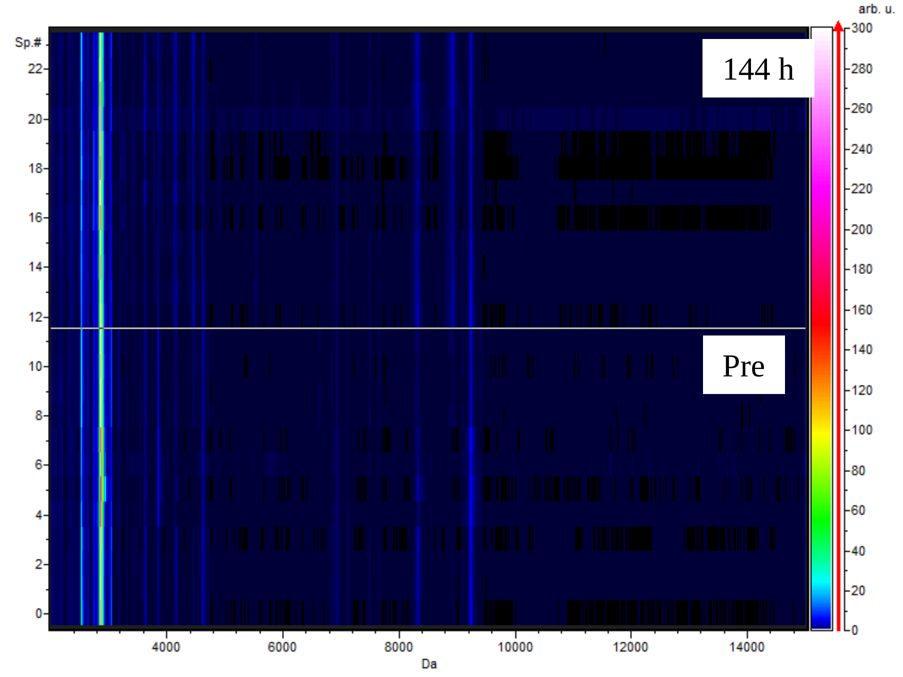
144 h
Pre
Sp.#
arb. u.
Da

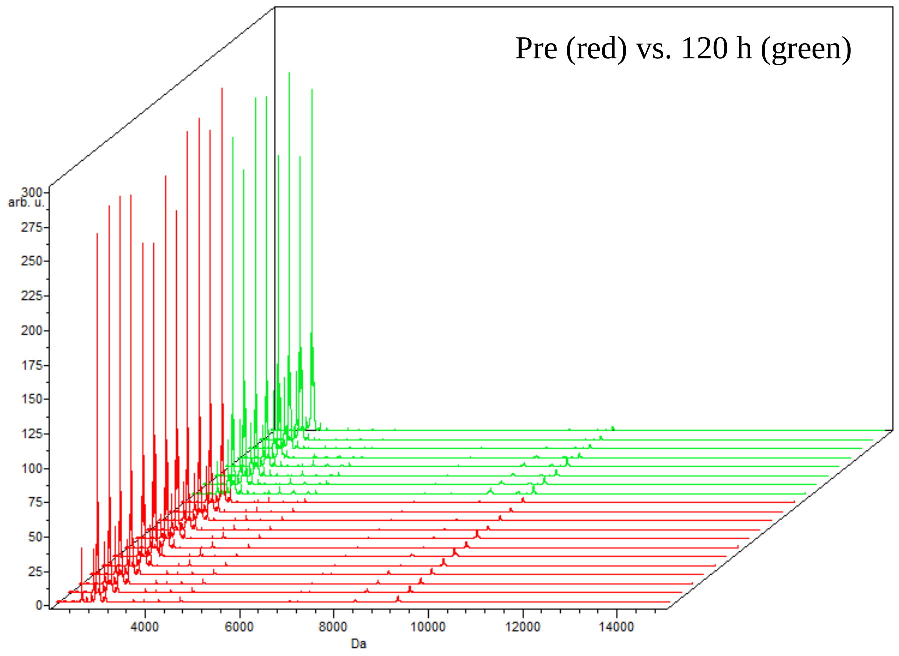
Pre (red) vs. 120 h (green)
arb. u.
Da

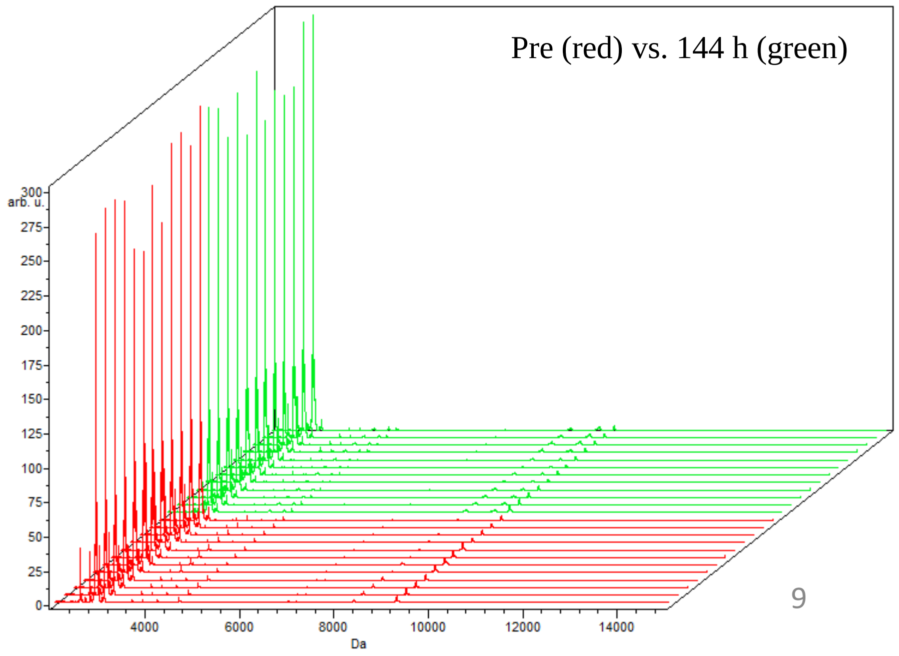
Pre (red) vs. 144 h (green)
arb. u.
Da

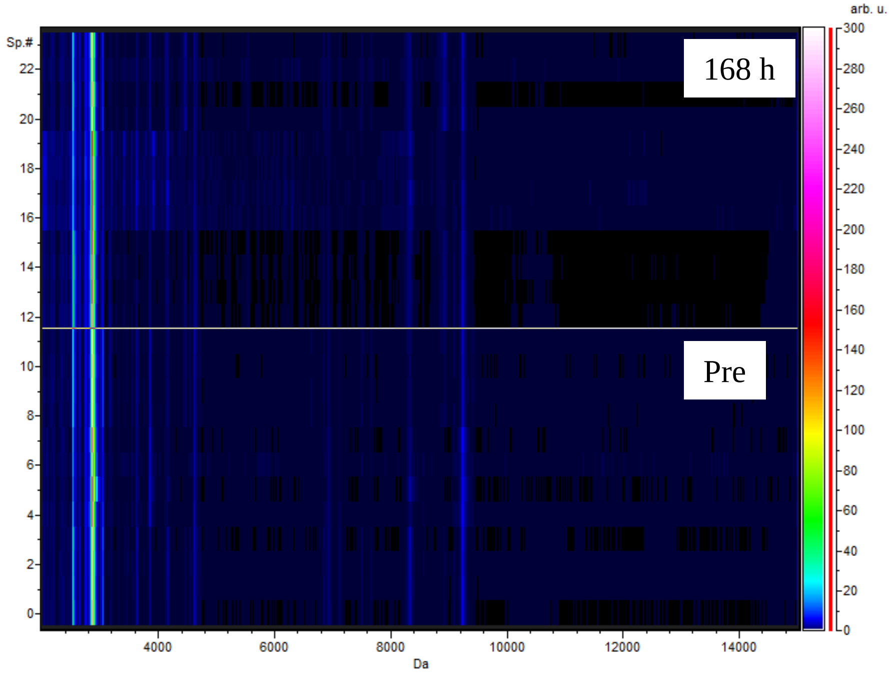
168 h
Pre
Sp.#
arb. u.
Da

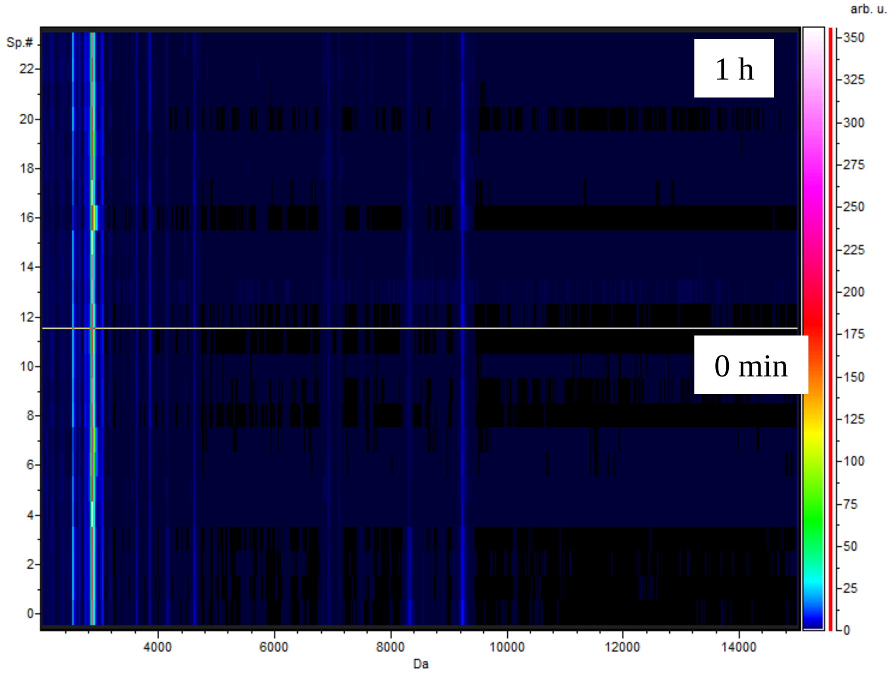
1 h
0 min
Sp.#
arb. u.
Da

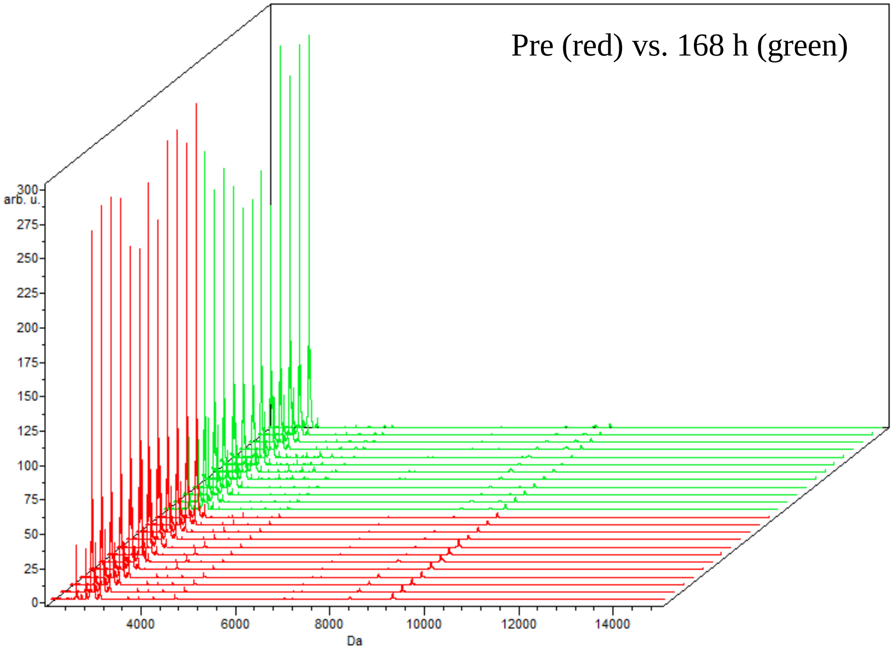
Pre (red) vs. 168 h (green)
arb. u.
Da

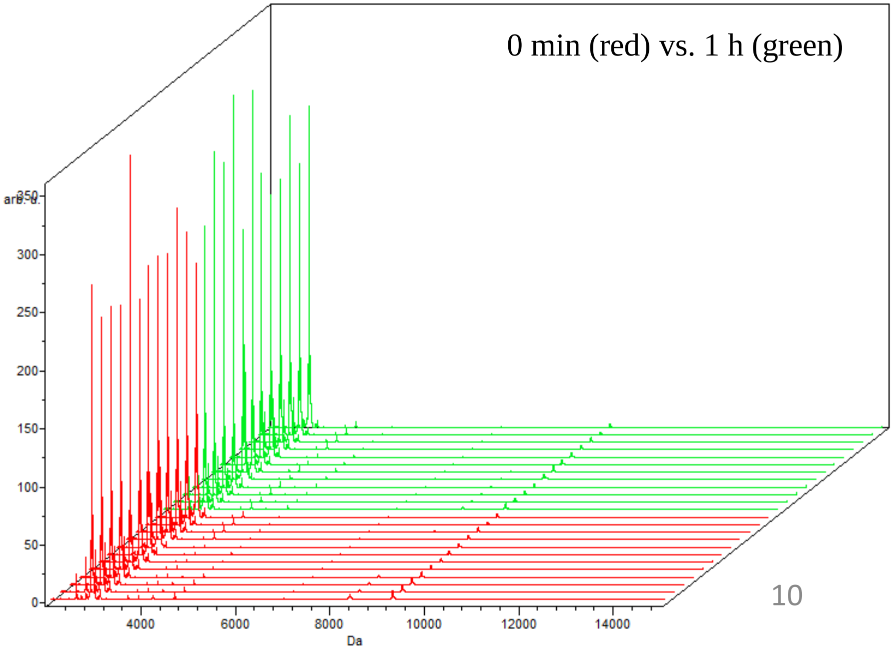
0 min (red) vs. 1 h (green)
arb. u.
Da

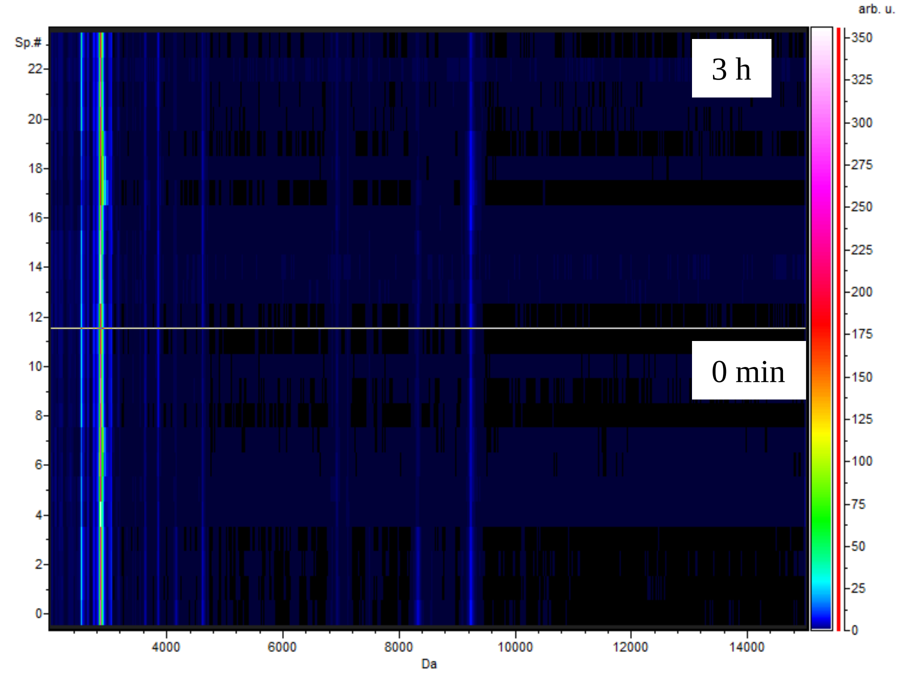
3 h
0 min
Sp.#
Da
arb. u.

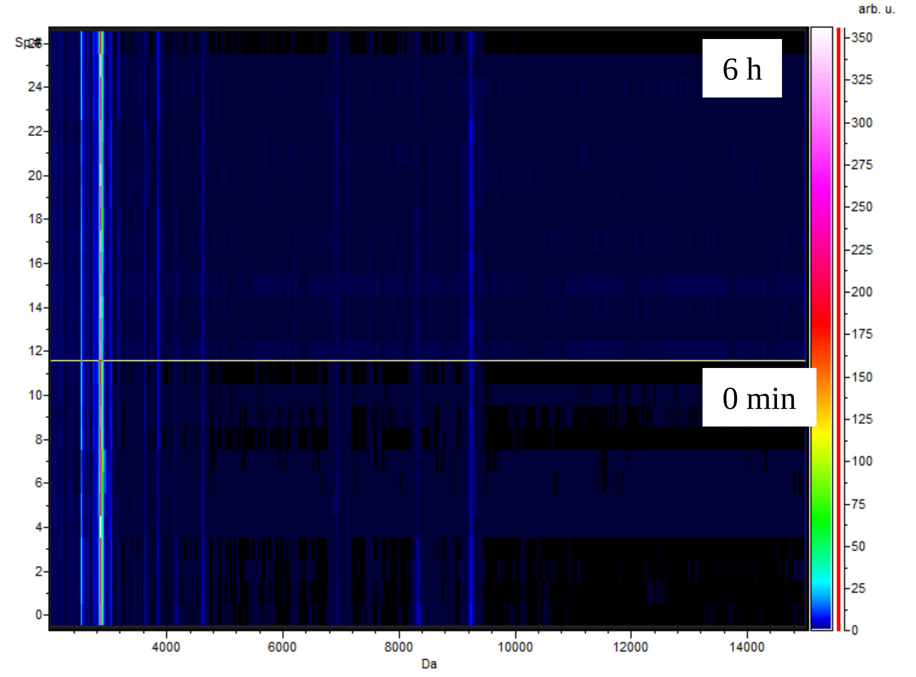
6 h
0 min
Sp.#
Da
arb. u.

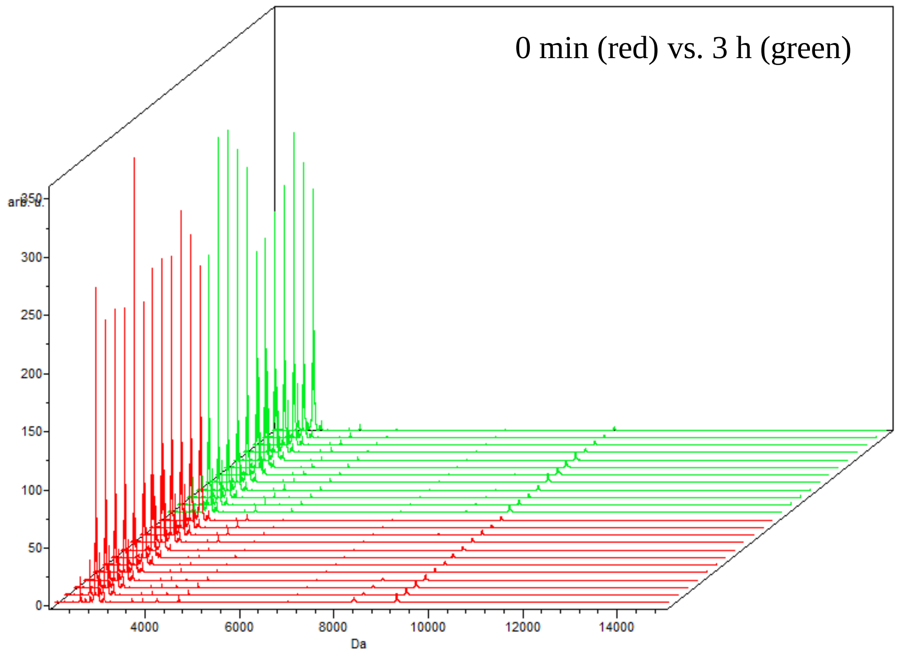
0 min (red) vs. 3 h (green)
arb. u.
Da

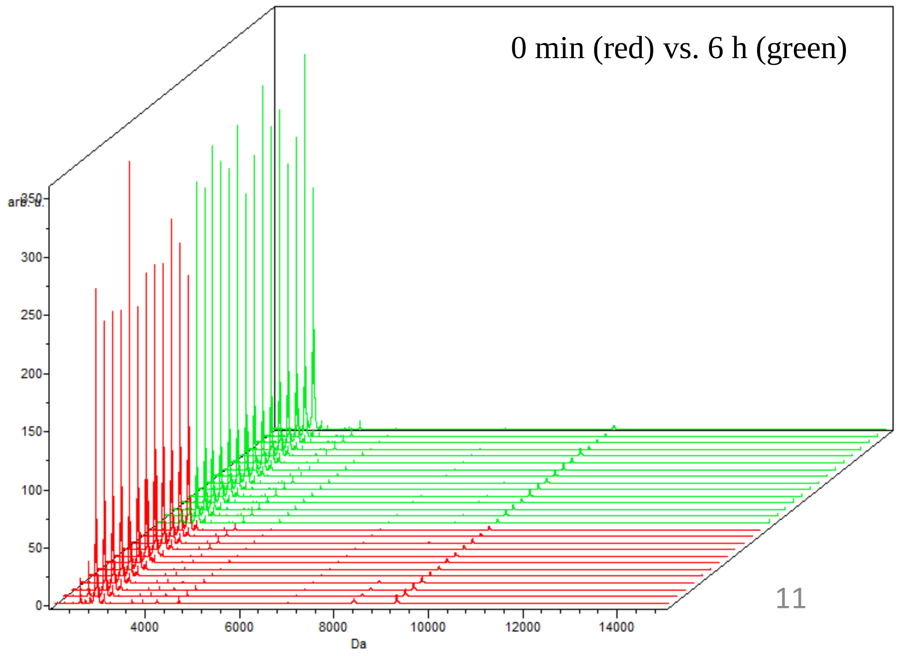
0 min (red) vs. 6 h (green)
arb. u.
Da

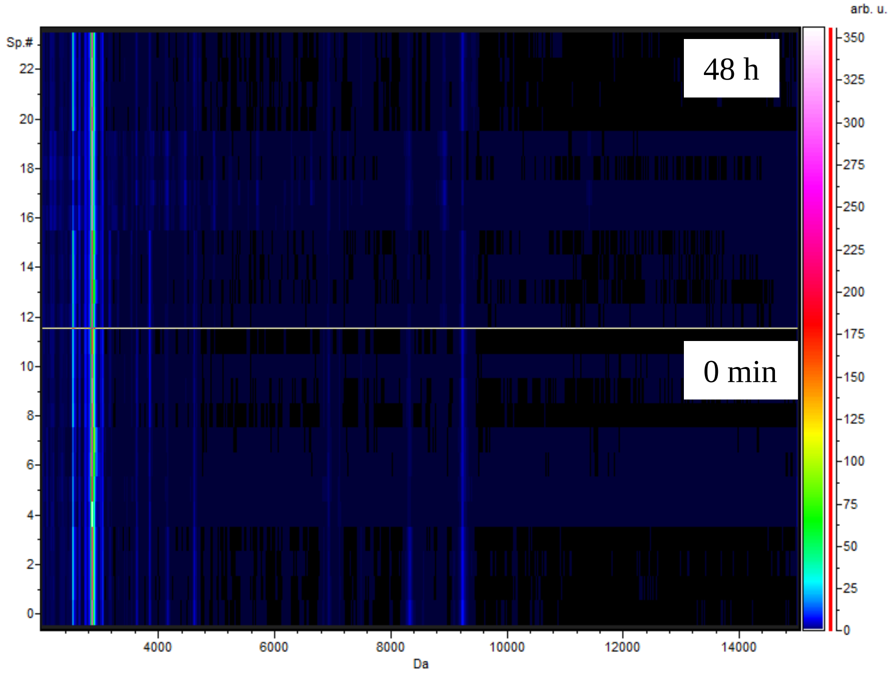
48 h
0 min
Sp.#
Da
arb. u.
4000
6000
8000
10000
12000
14000

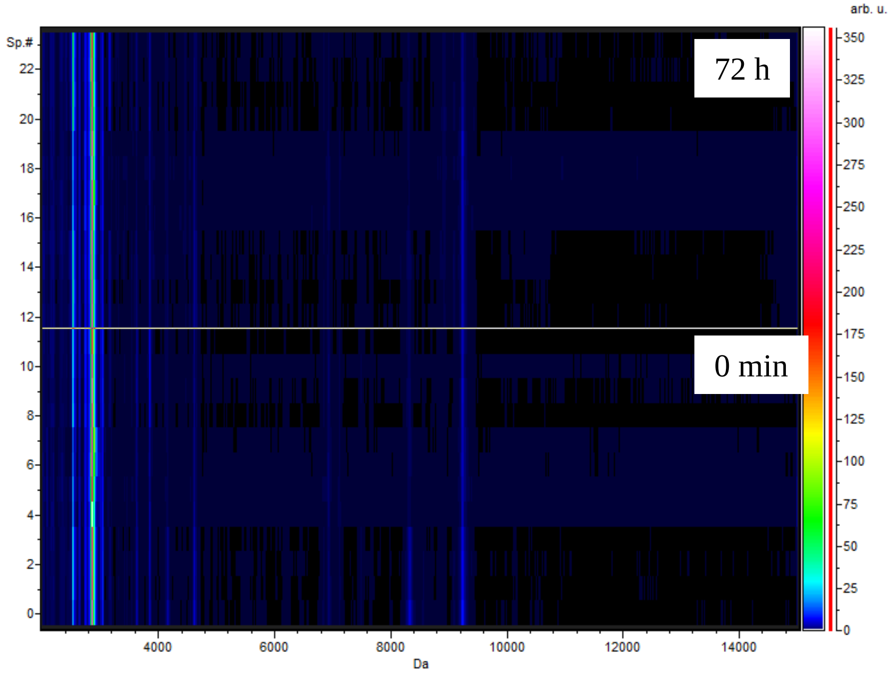
72 h
0 min
Sp.#
Da
arb. u.
4000
6000
8000
10000
12000
14000

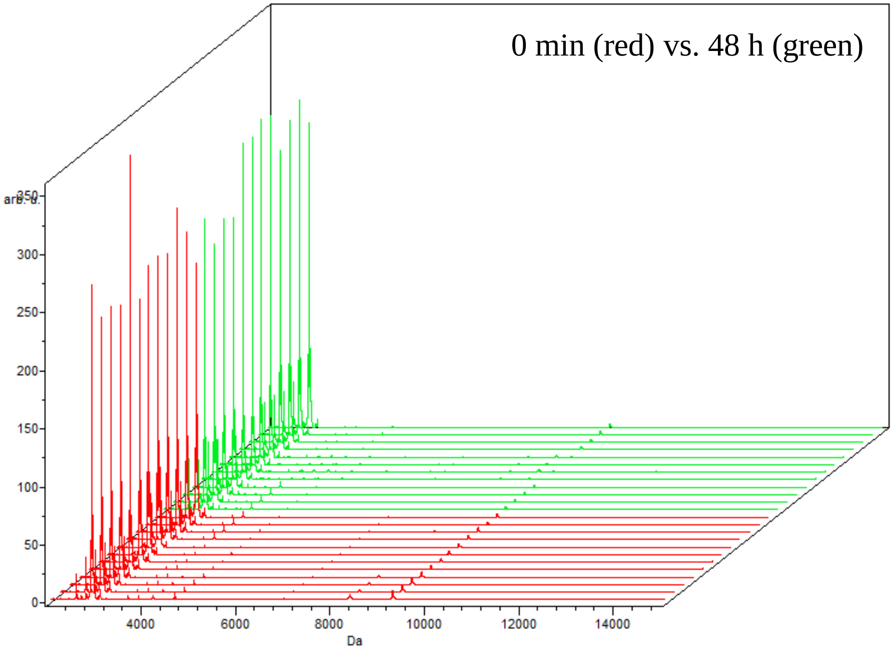
0 min (red) vs. 48 h (green)
arb. u.
Da
4000
6000
8000
10000
12000
14000

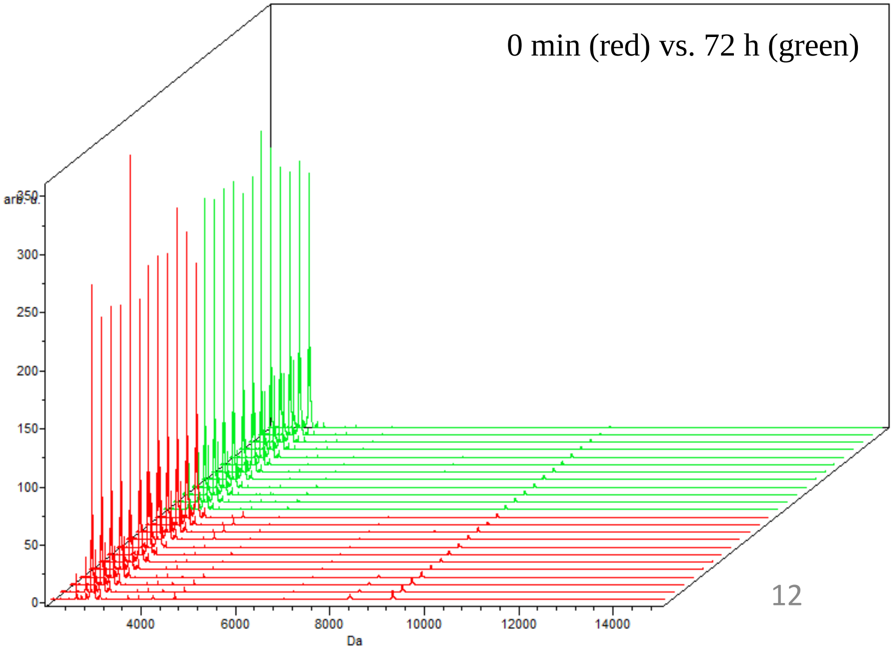
0 min (red) vs. 72 h (green)
arb. u.
Da
4000
6000
8000
10000
12000
14000

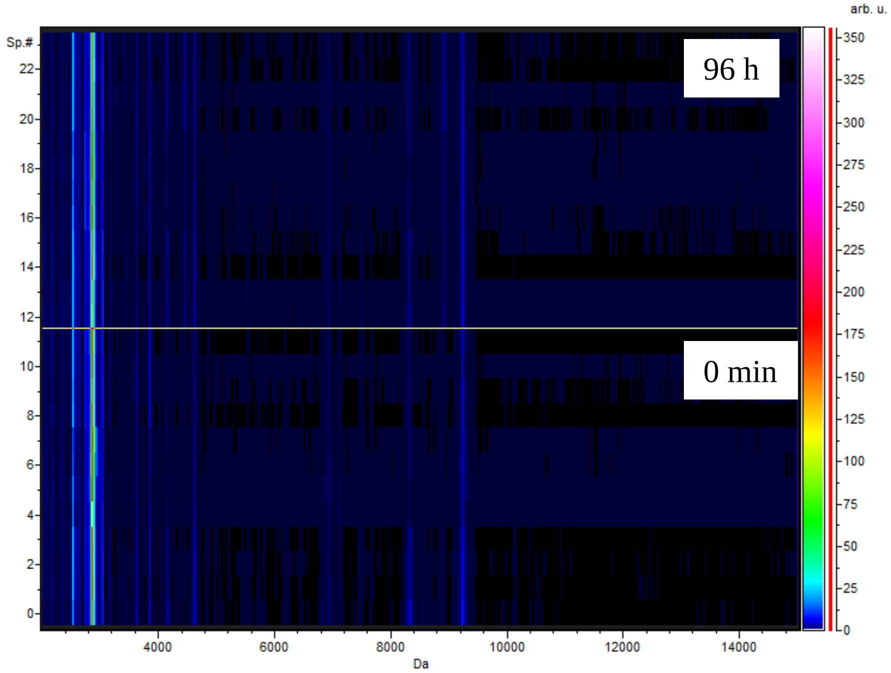
96 h
0 min
Sp.#
Da
arb. u.

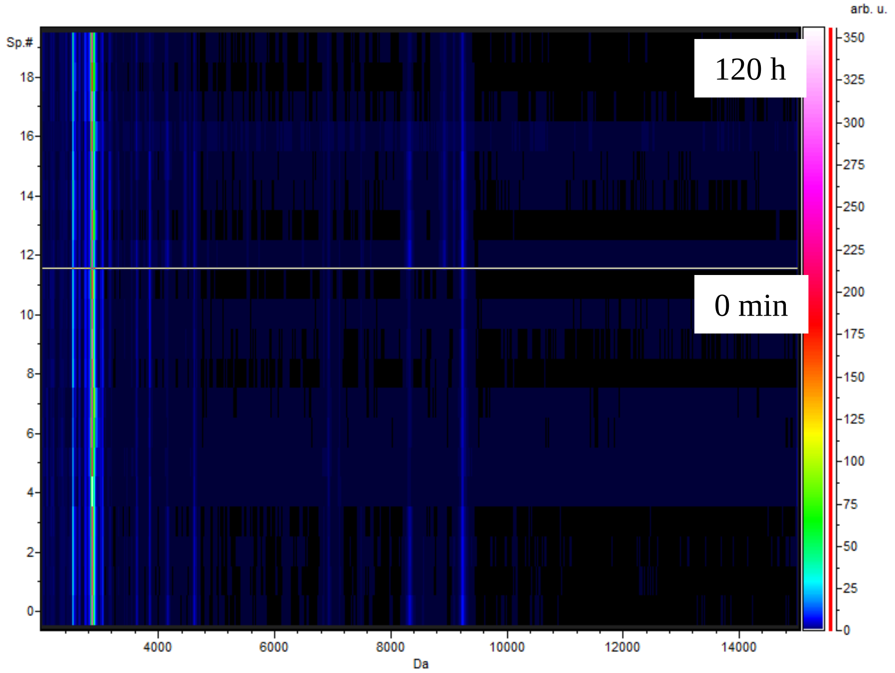
120 h
0 min
Sp.#
Da
arb. u.

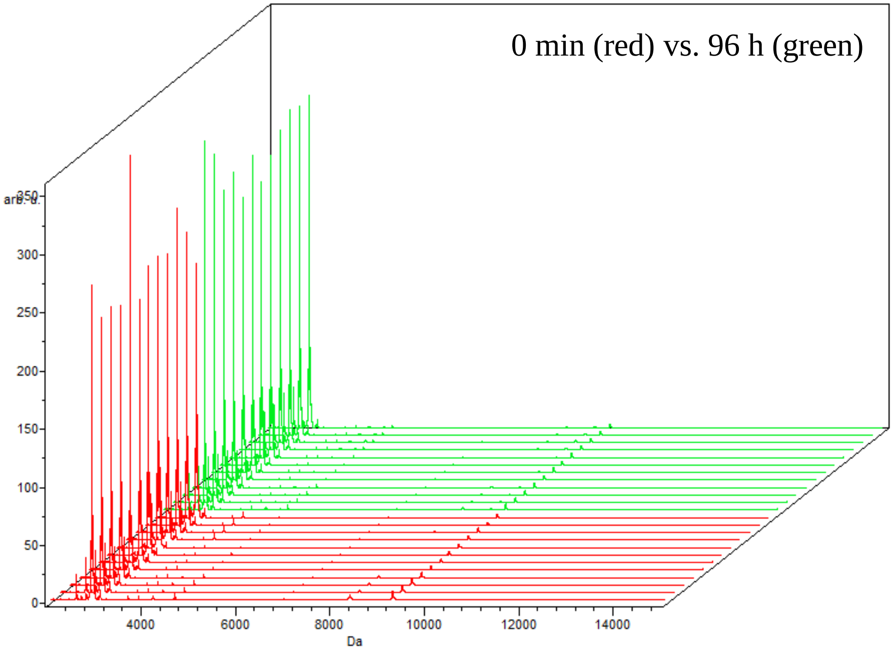
0 min (red) vs. 96 h (green)
arb. u.
Da

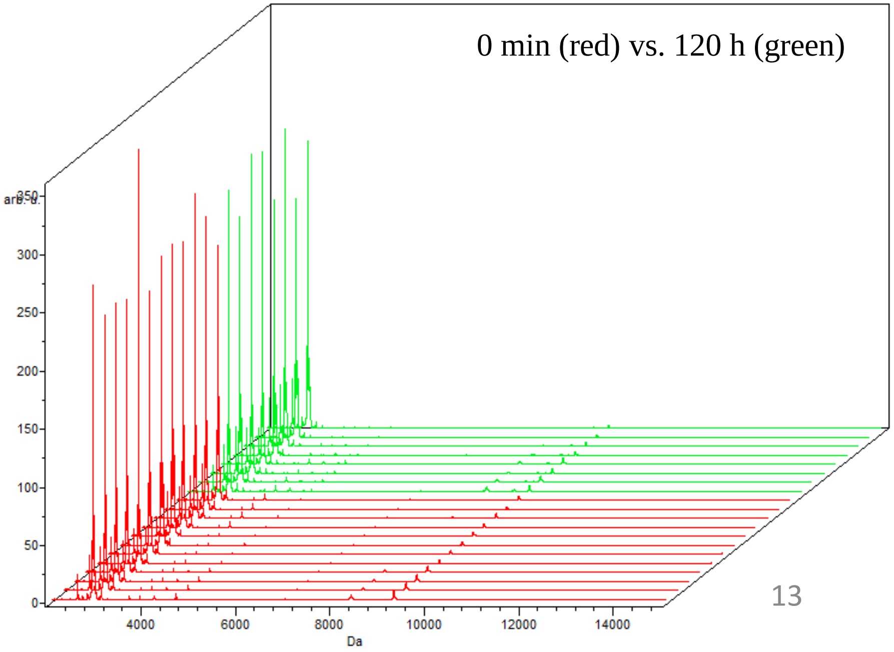
0 min (red) vs. 120 h (green)
arb. u.
Da

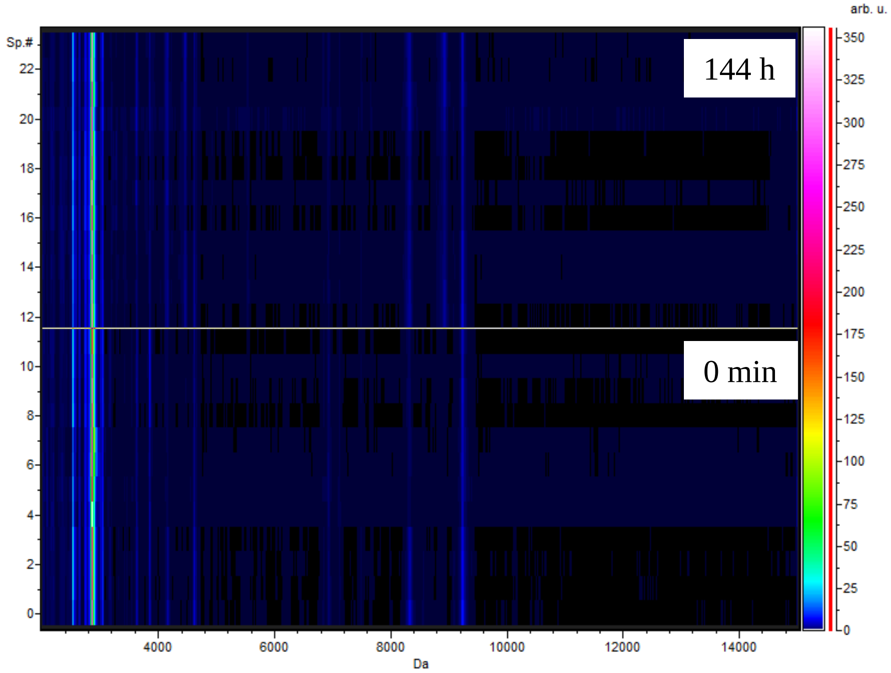
144 h
0 min
Sp.#
arb. u.
Da

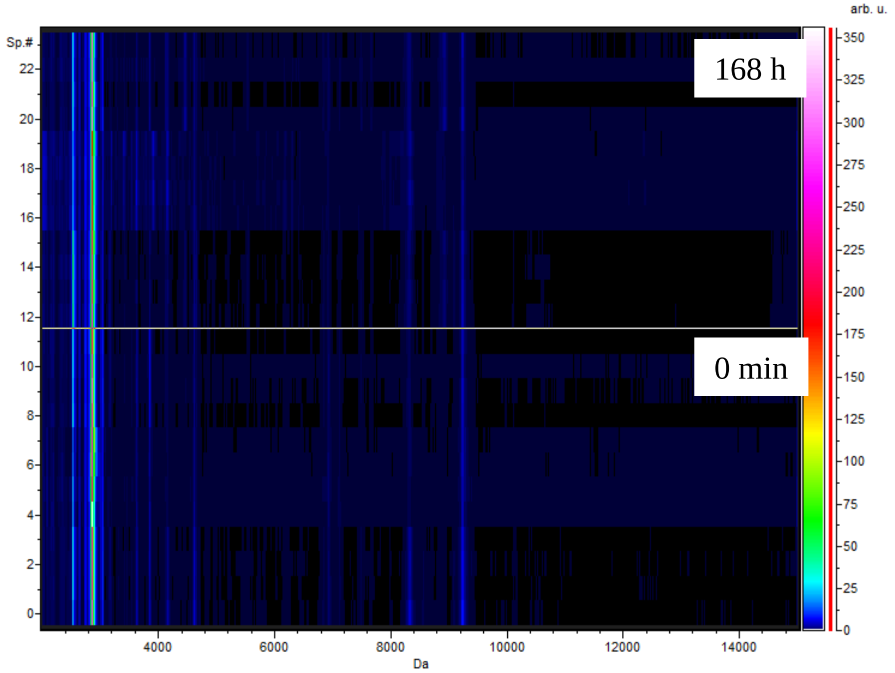
168 h
0 min
Sp.#
arb. u.
Da

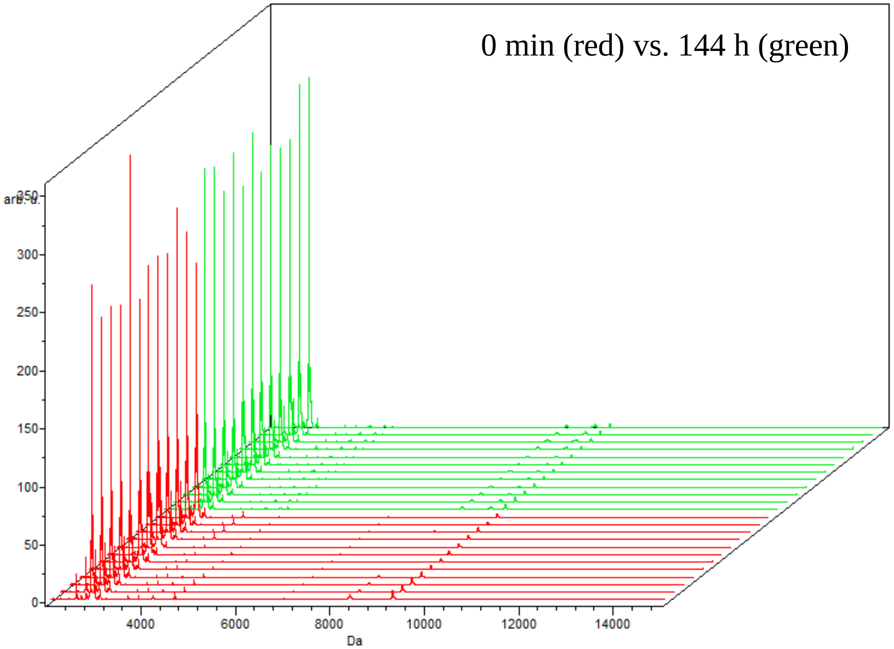
0 min (red) vs. 144 h (green)
arb. u.
Da

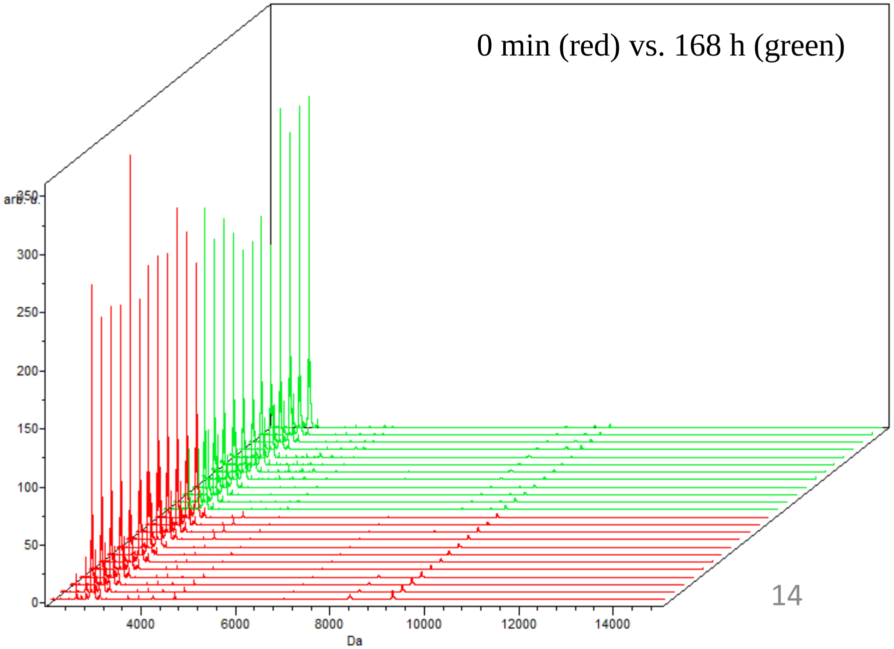
0 min (red) vs. 168 h (green)
arb. u.
Da

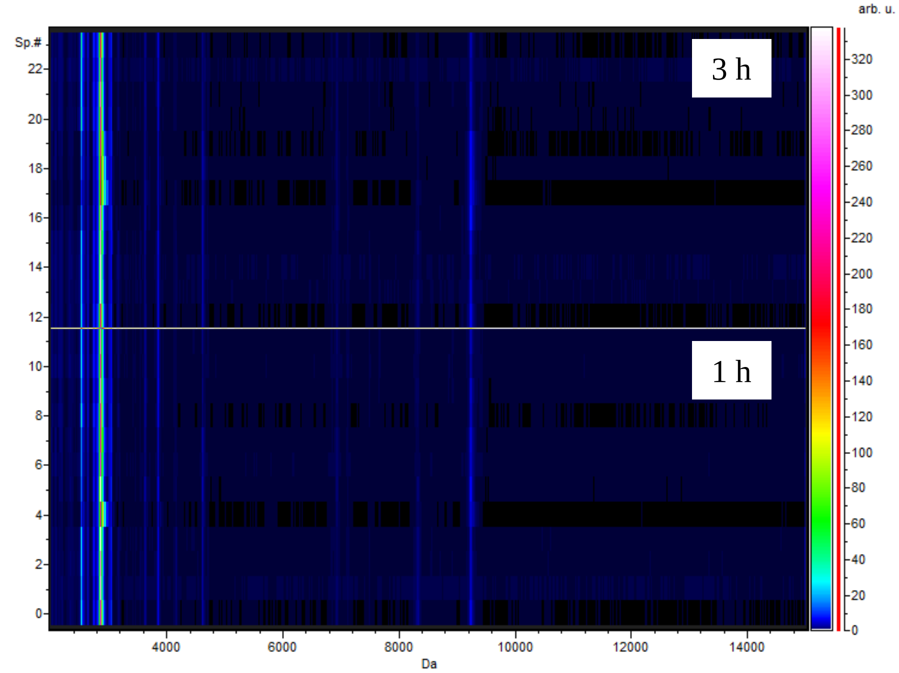
3 h
1 h
Sp.#
arb. u.
Da

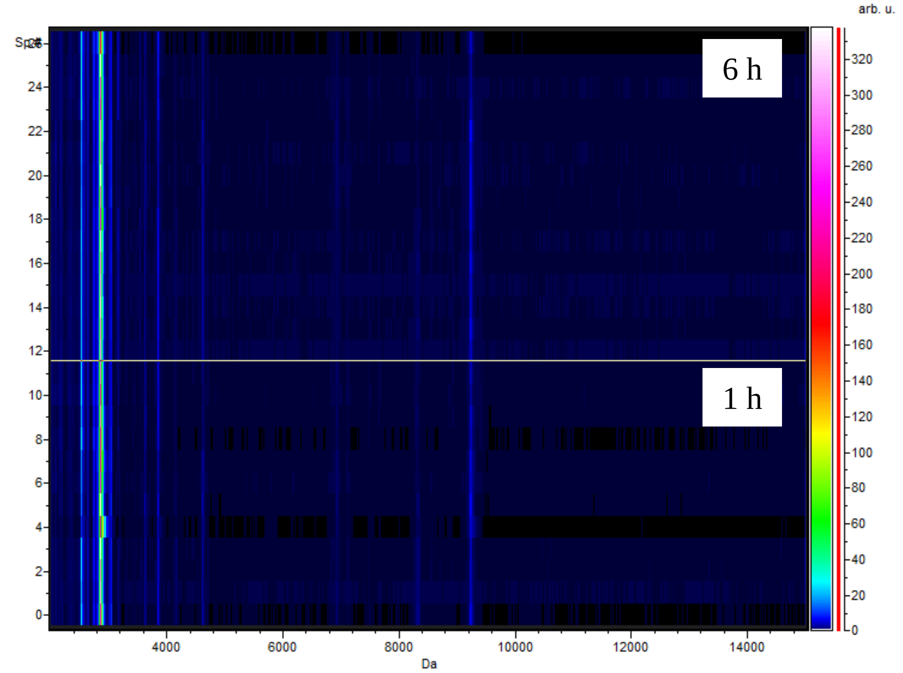
6 h
1 h
Sp.#
arb. u.
Da

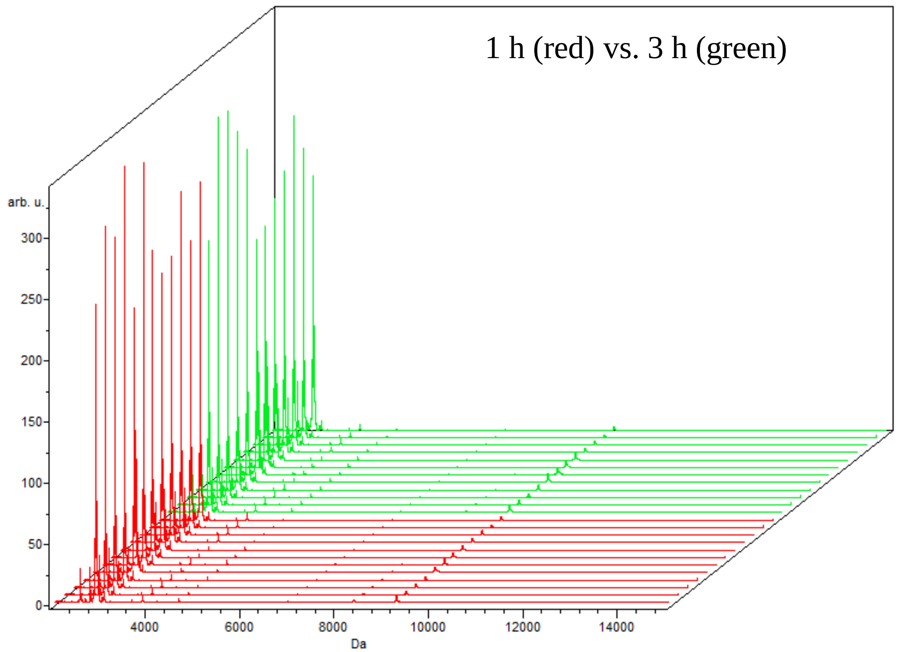
1 h (red) vs. 3 h (green)
arb. u.
Da

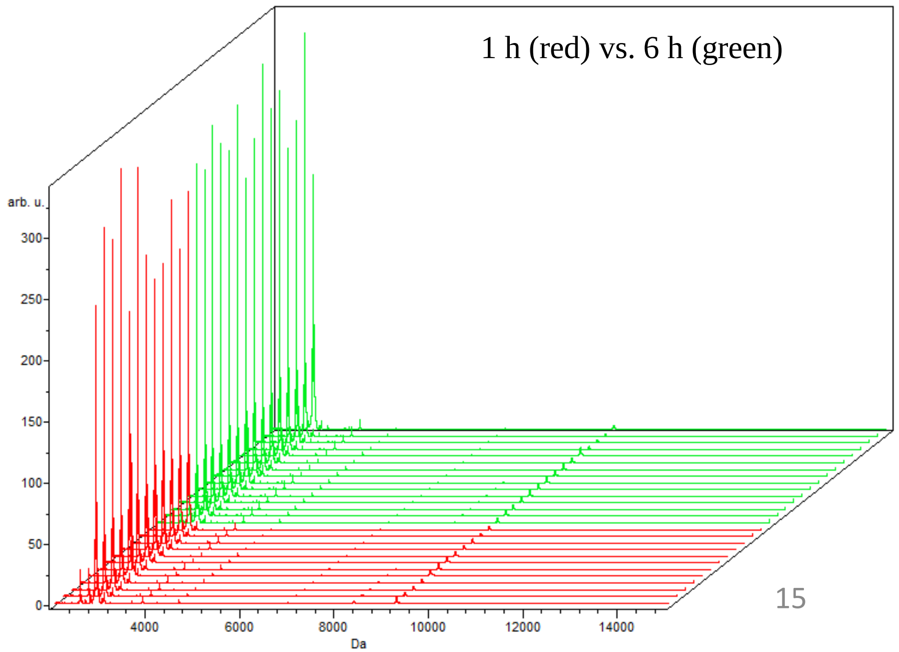
1 h (red) vs. 6 h (green)
arb. u.
Da

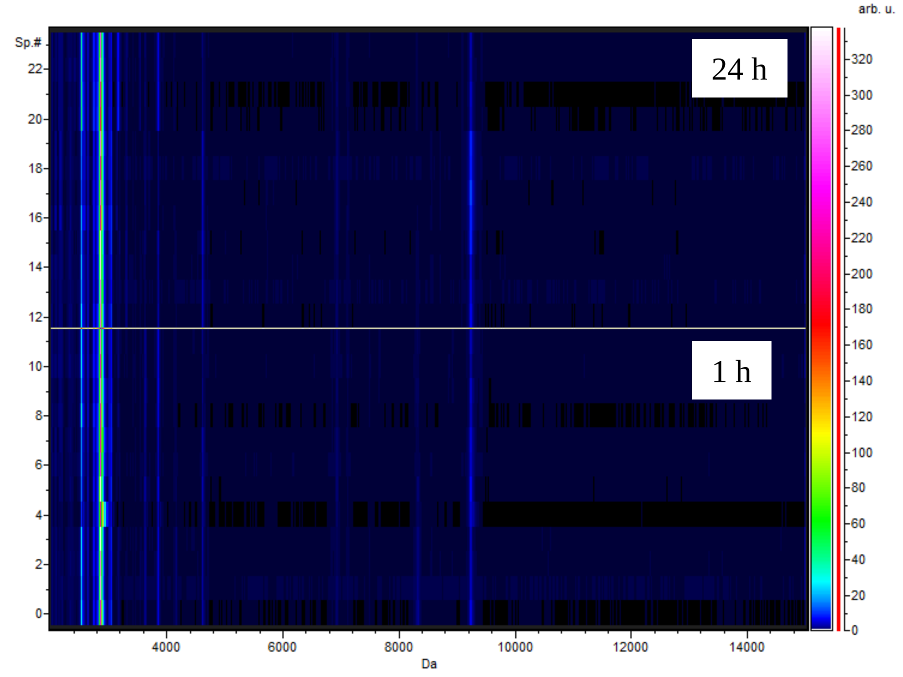
24 h
1 h
Sp.#
Da
arb. u.

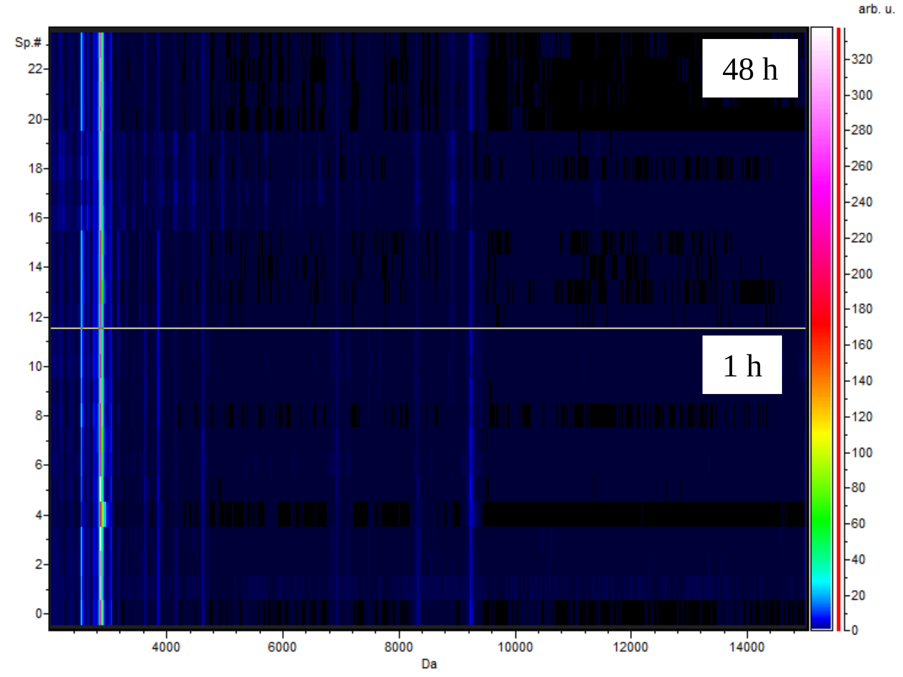
48 h
1 h
Sp.#
Da
arb. u.

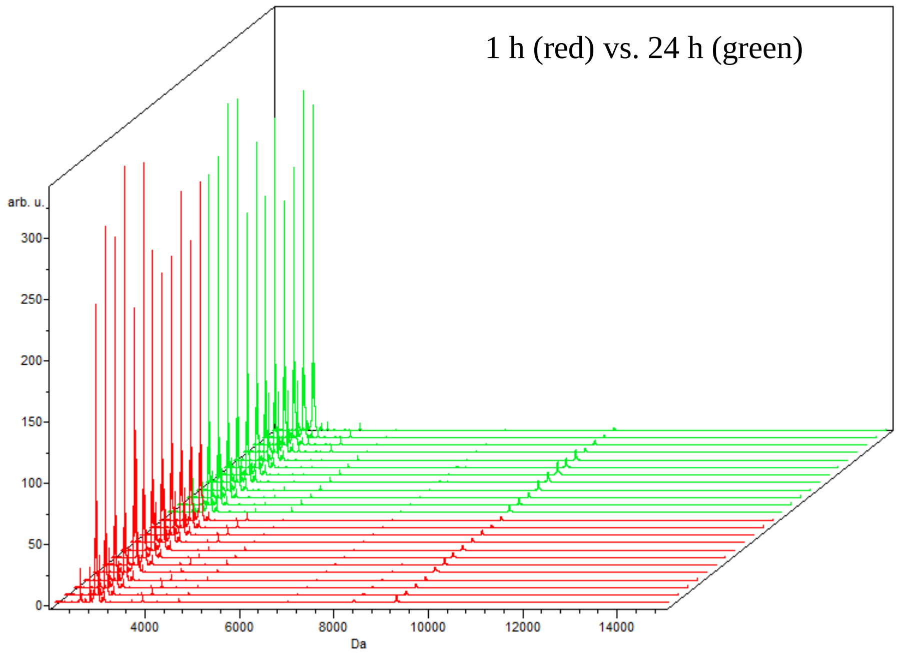
1 h (red) vs. 24 h (green)
arb. u.
Da

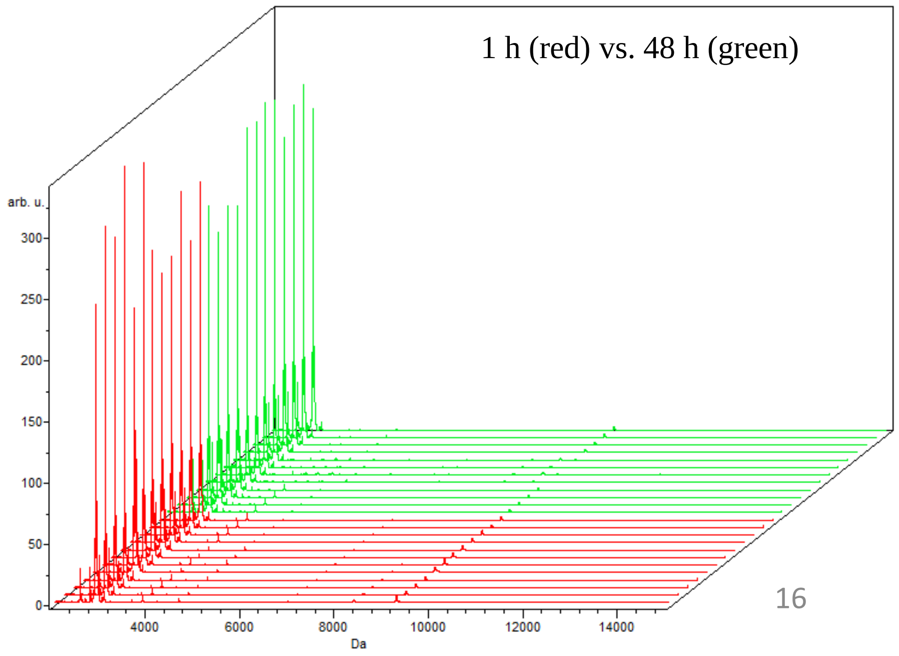
1 h (red) vs. 48 h (green)
arb. u.
Da

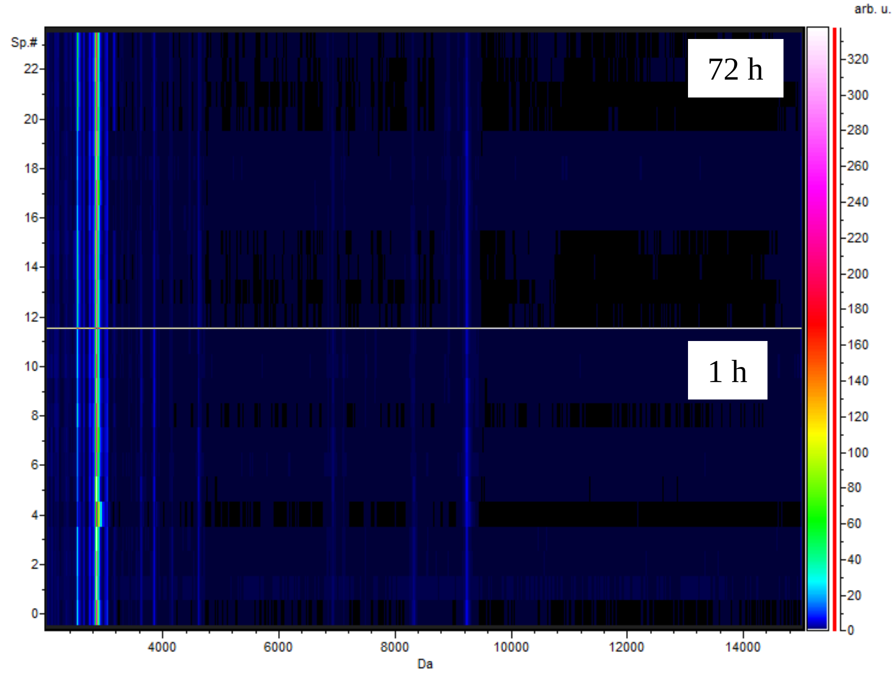
72 h
1 h
Sp.#
Da
arb. u.

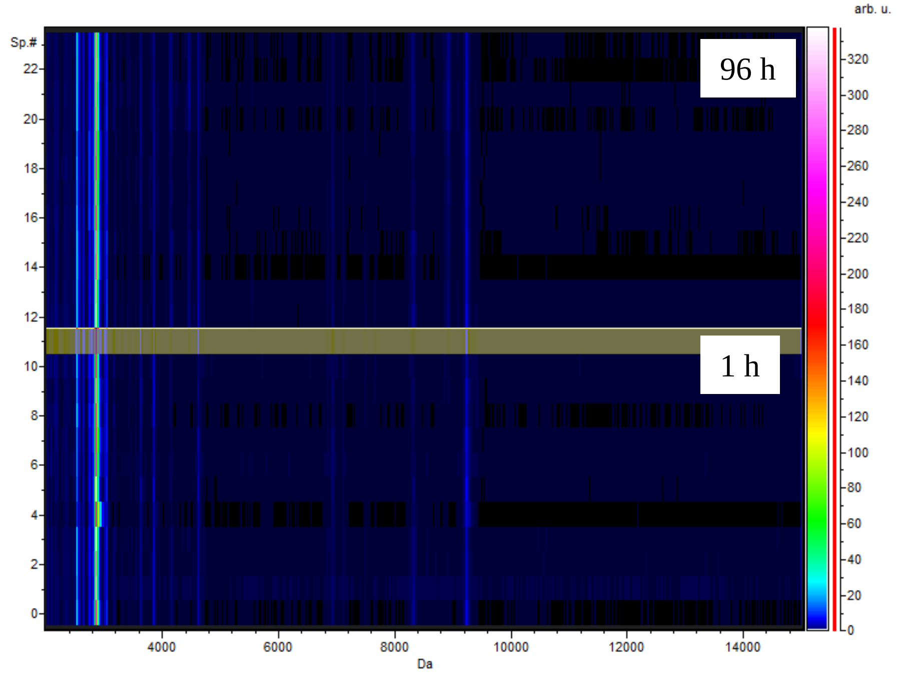
96 h
1 h
Sp.#
Da
arb. u.

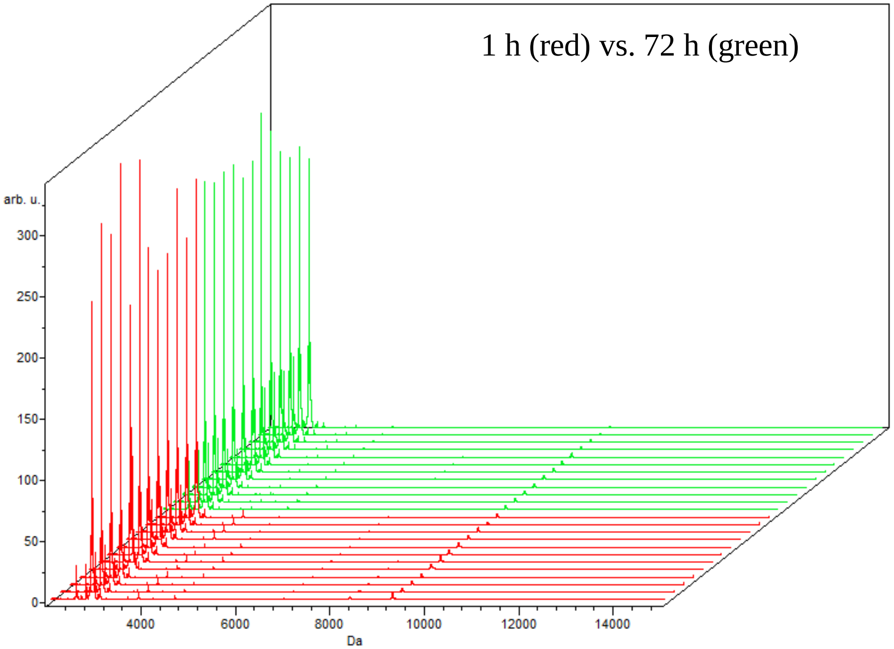
1 h (red) vs. 72 h (green)
arb. u.
Da

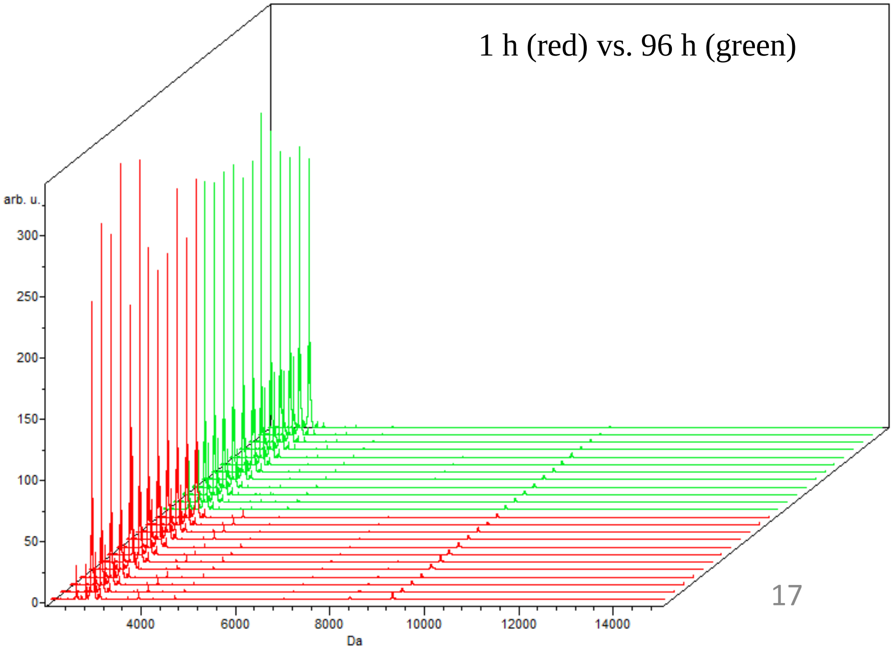
1 h (red) vs. 96 h (green)
arb. u.
Da

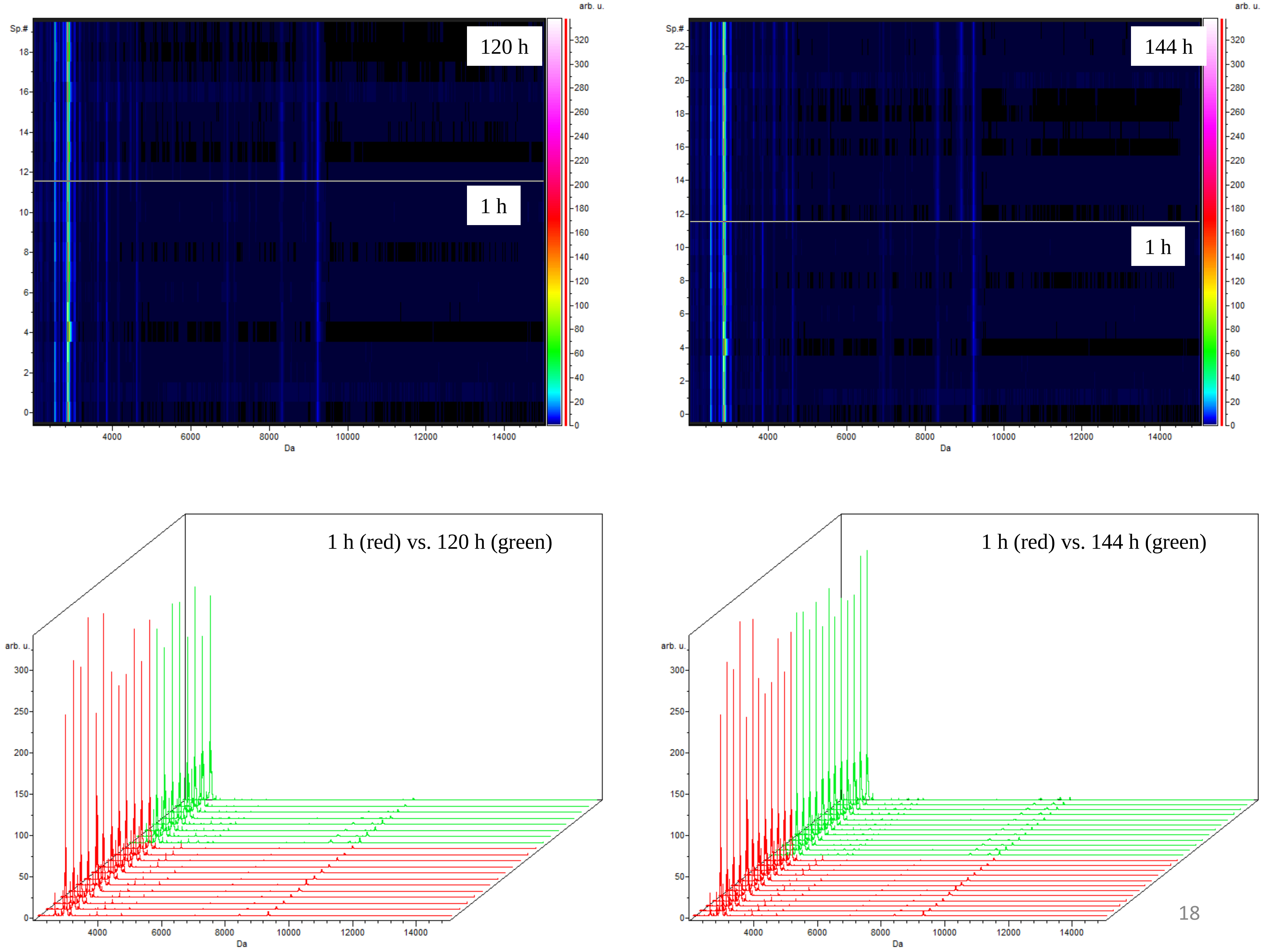

120 h
1 h
144 h
1 h
1 h (red) vs. 120 h (green)
1 h (red) vs. 144 h (green)
Sp.#
Da
arb. u.

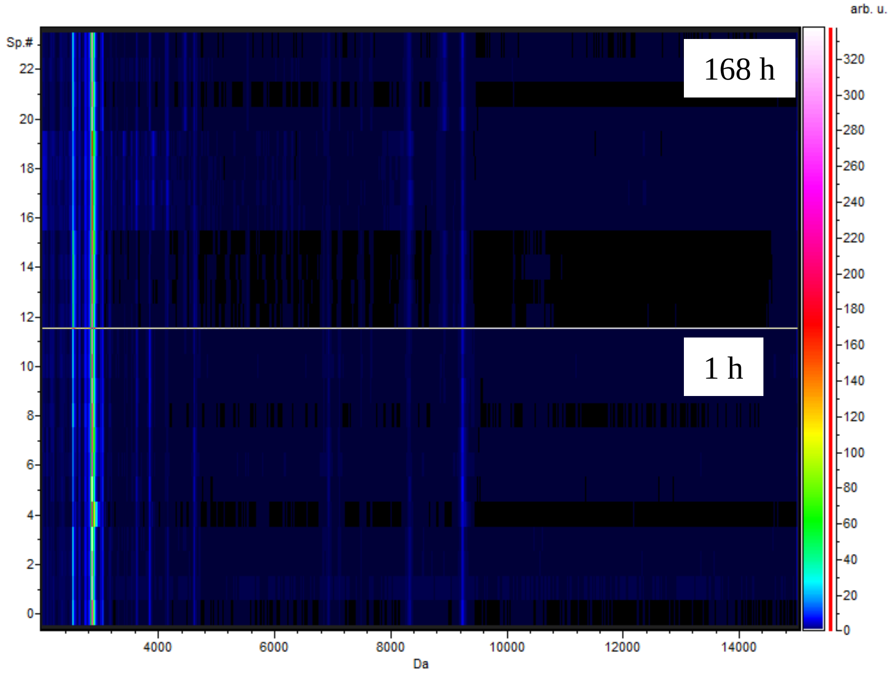
168 h
1 h
Sp.#
arb. u.
Da

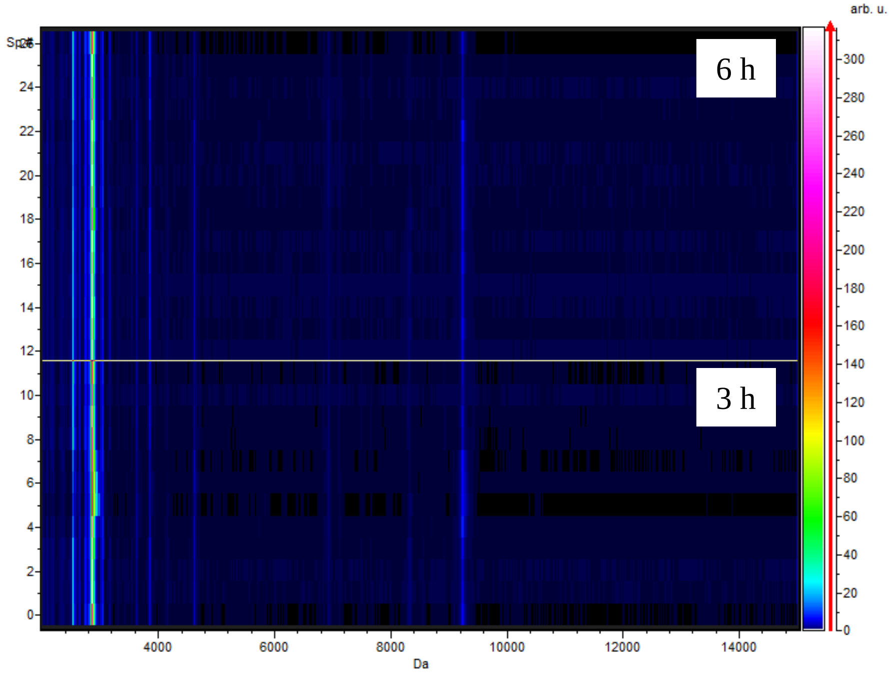
6 h
3 h
Sp.#
arb. u.
Da

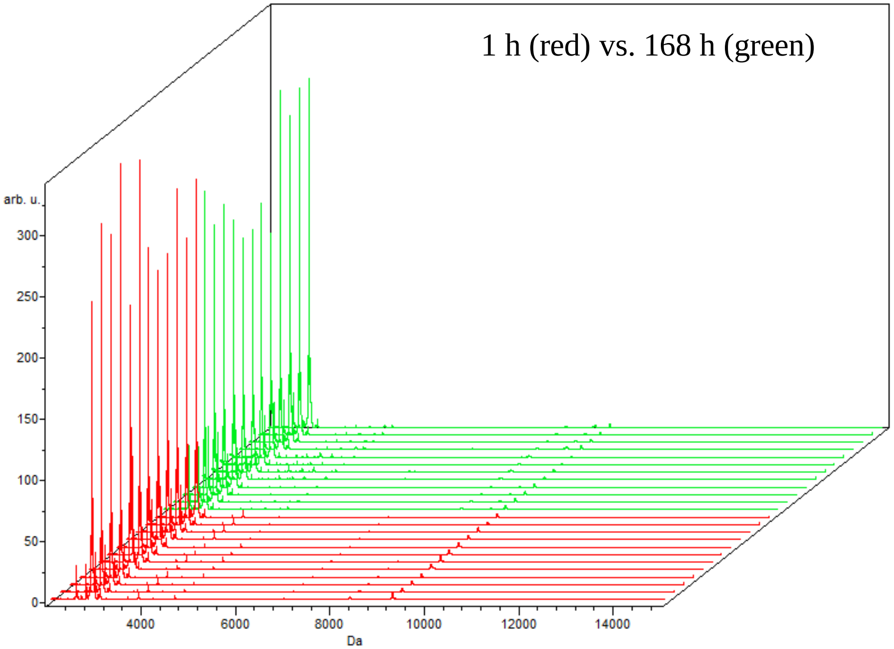
1 h (red) vs. 168 h (green)
arb. u.
Da

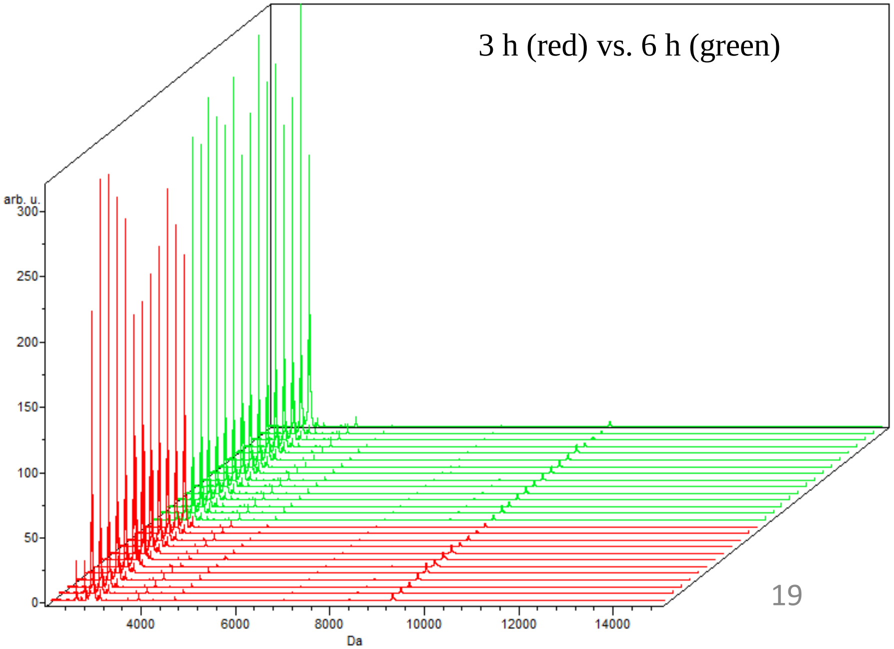
3 h (red) vs. 6 h (green)
arb. u.
Da

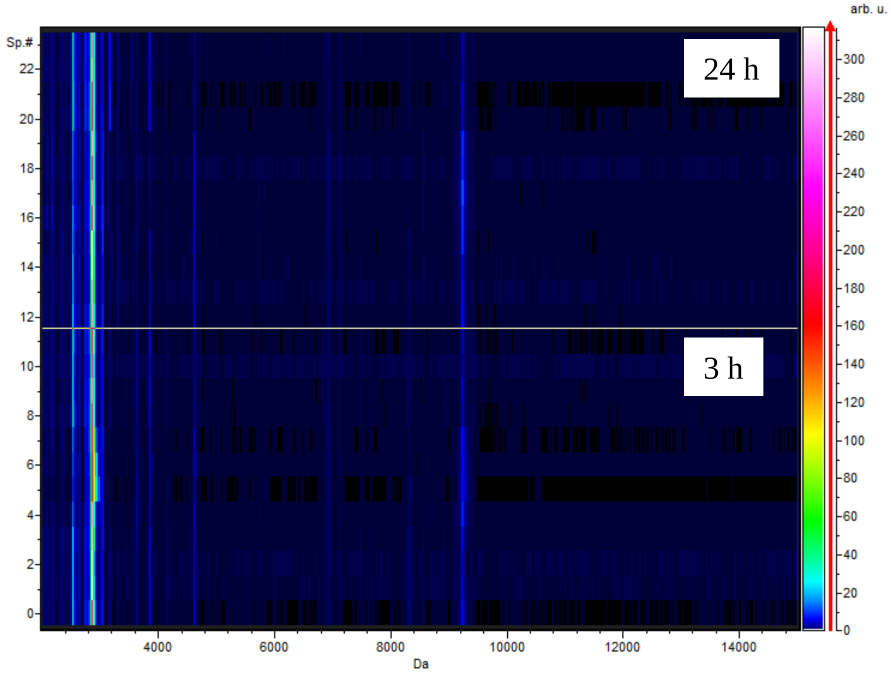
24 h
3 h
Sp.#
arb. u.
Da

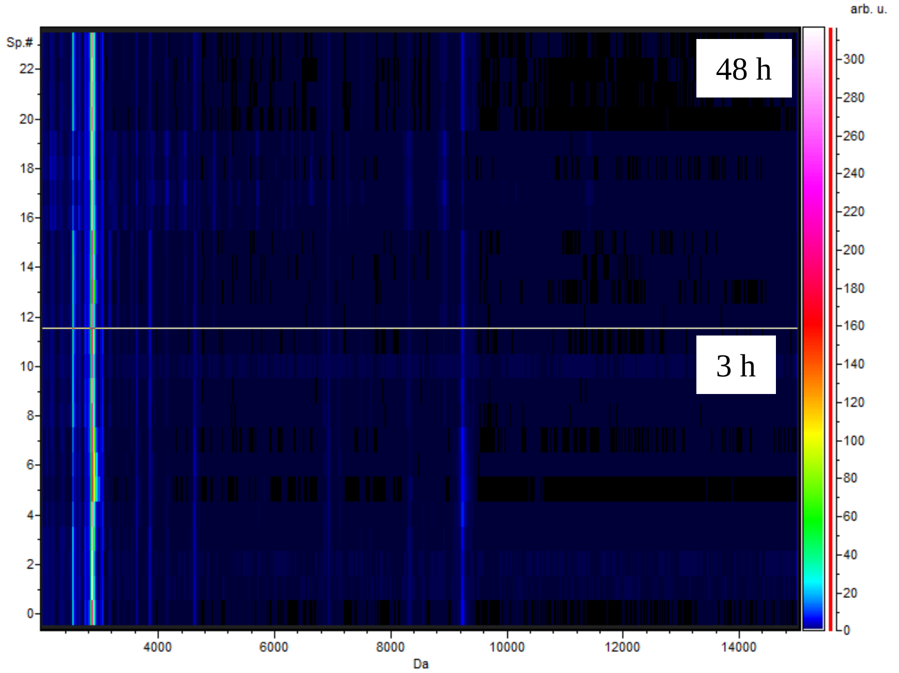
48 h
3 h
Sp.#
arb. u.
Da

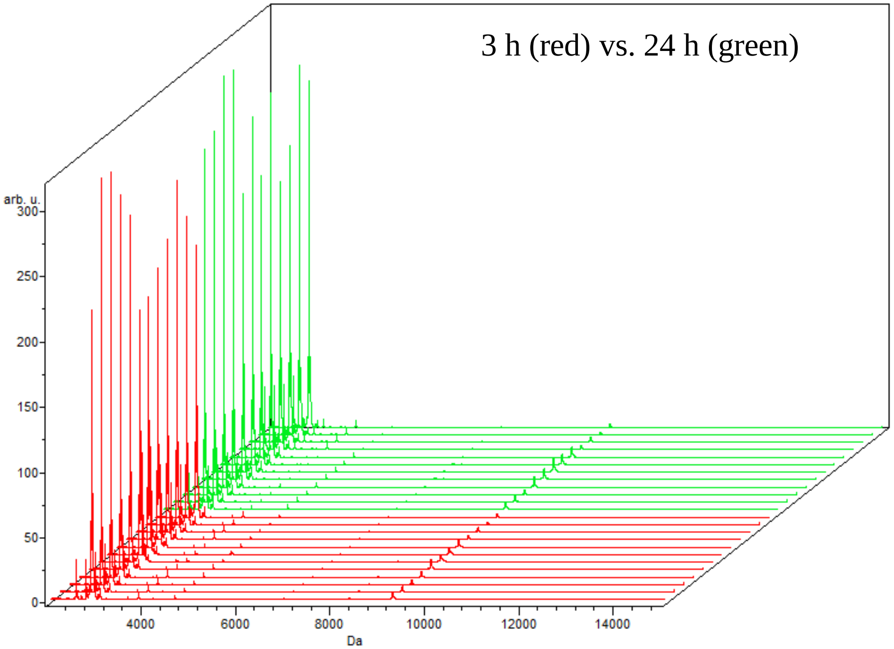
3 h (red) vs. 24 h (green)
arb. u.
Da

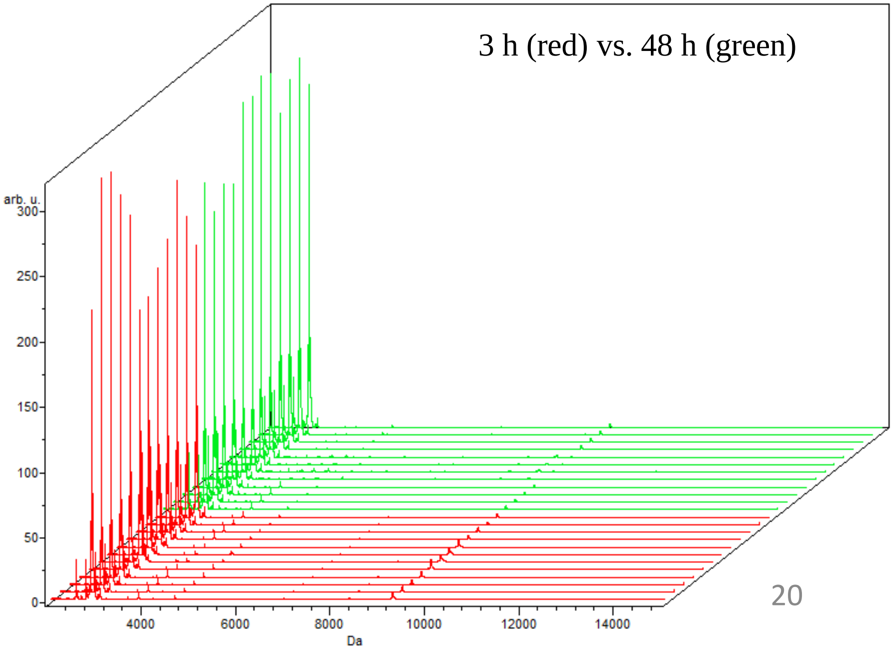
3 h (red) vs. 48 h (green)
arb. u.
Da

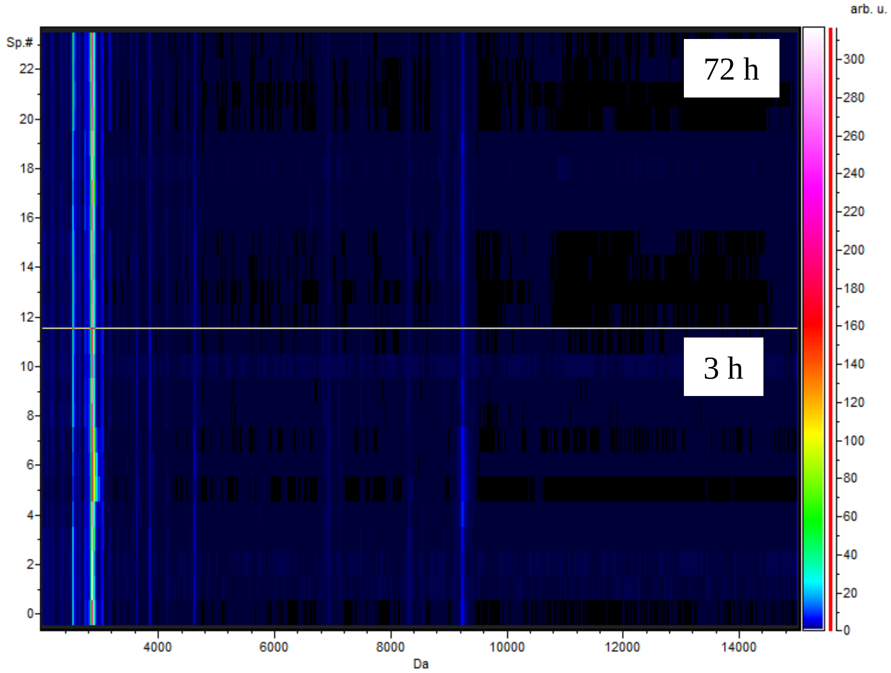
72 h
3 h
Sp.#
Da
arb. u.

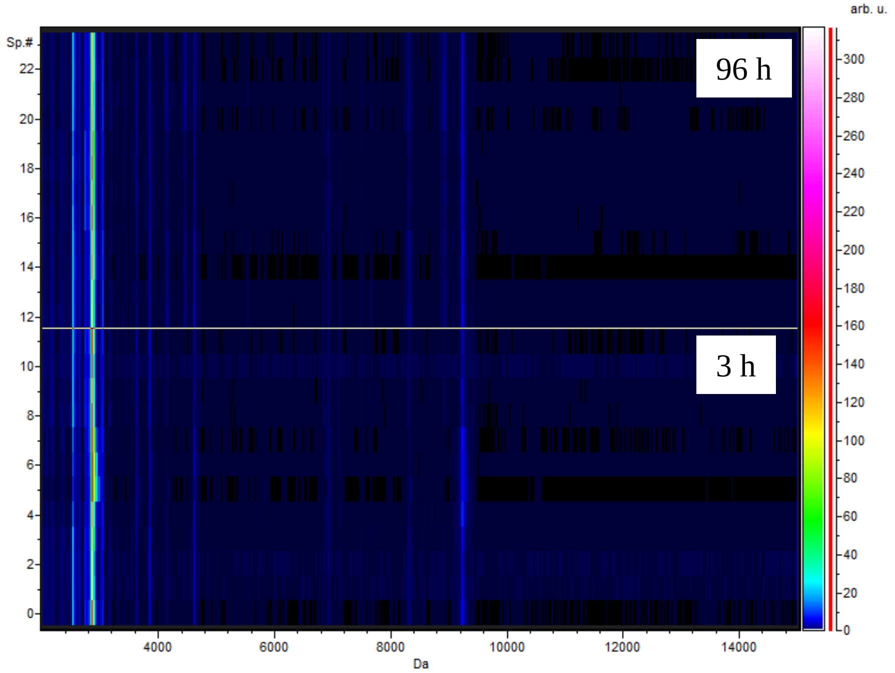
96 h
3 h
Sp.#
Da
arb. u.

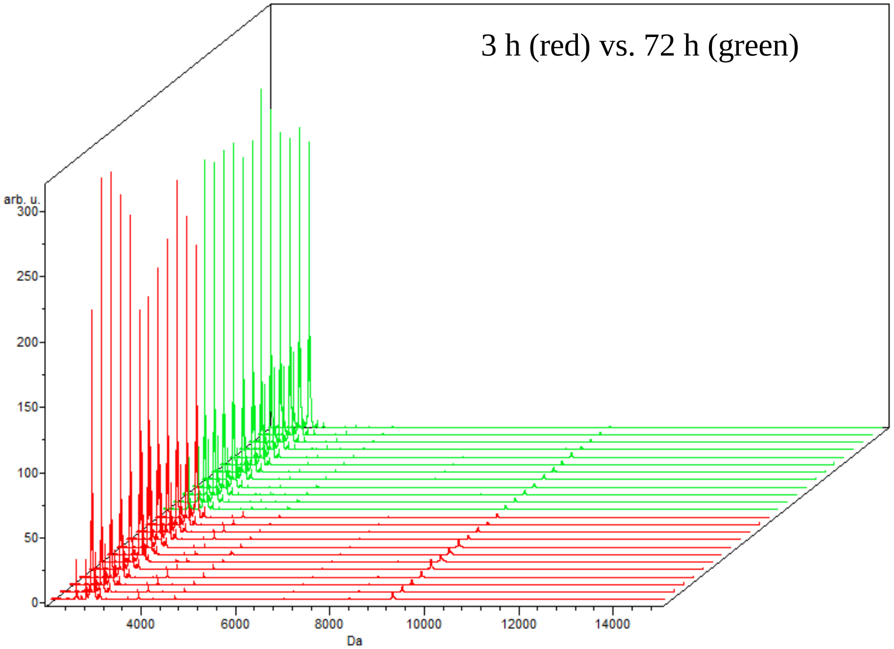
3 h (red) vs. 72 h (green)
arb. u.
Da

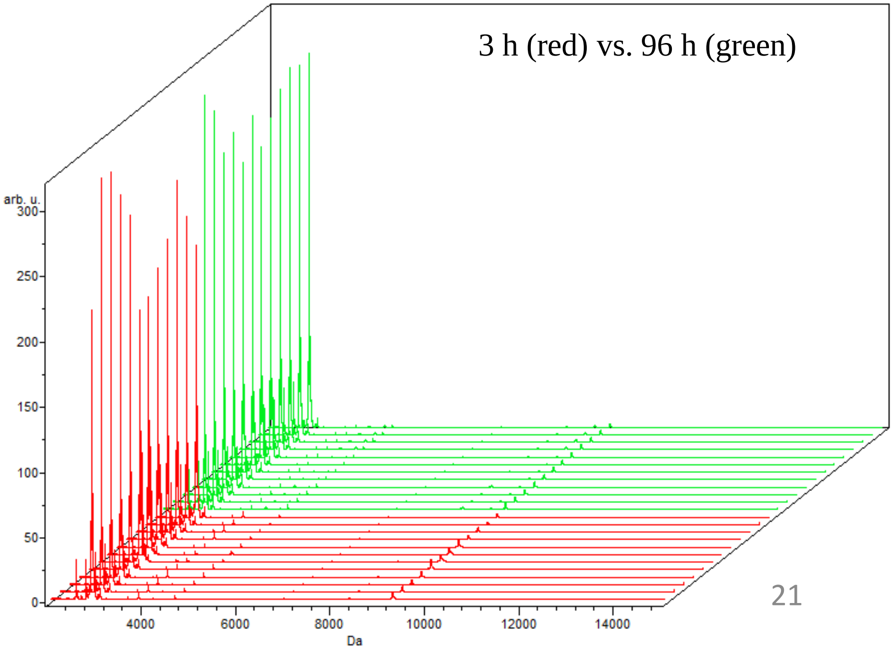
3 h (red) vs. 96 h (green)
arb. u.
Da

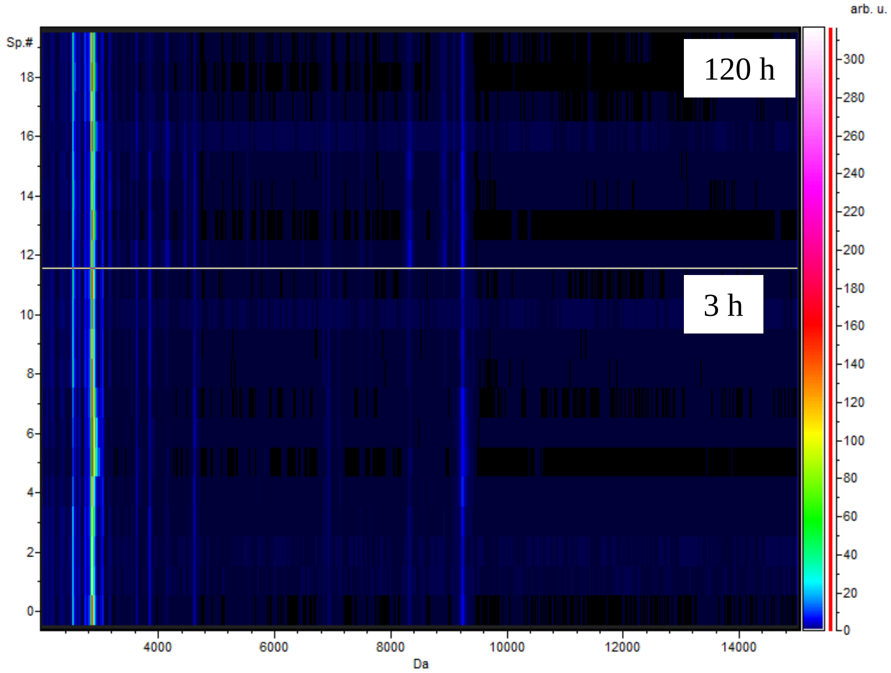
120 h
3 h
Sp.#
arb. u.
Da

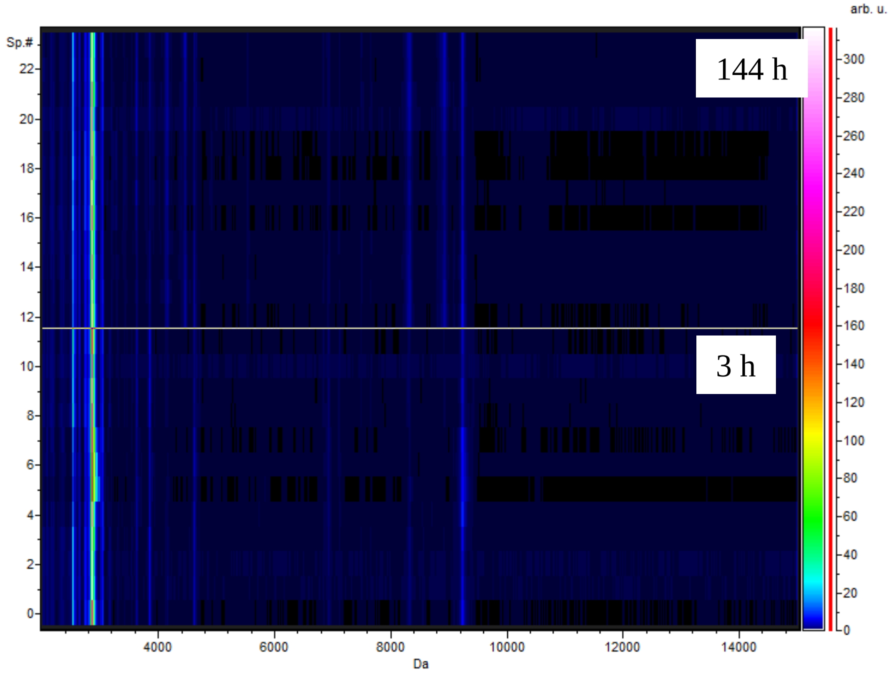
144 h
3 h
Sp.#
arb. u.
Da

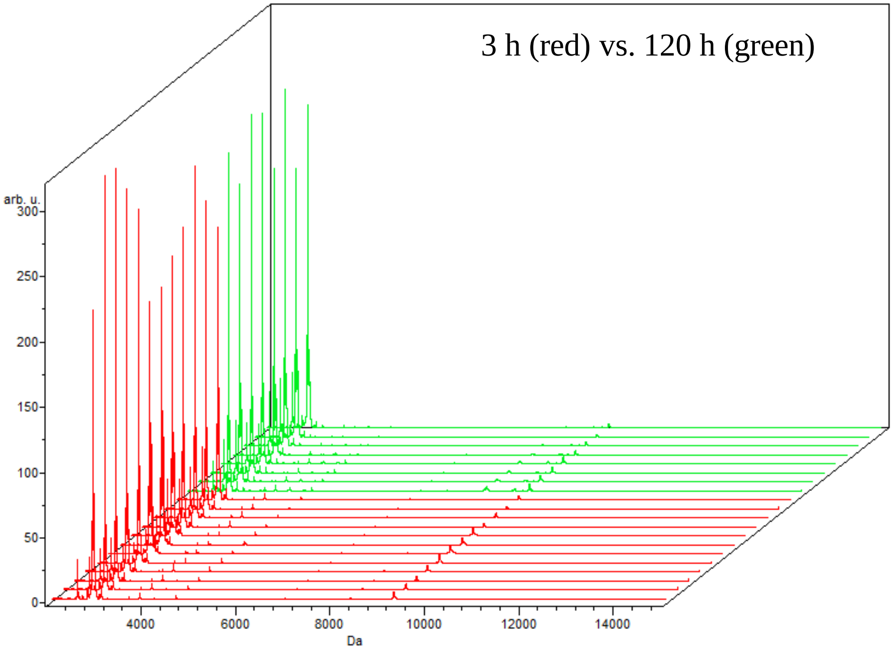
3 h (red) vs. 120 h (green)
arb. u.
Da

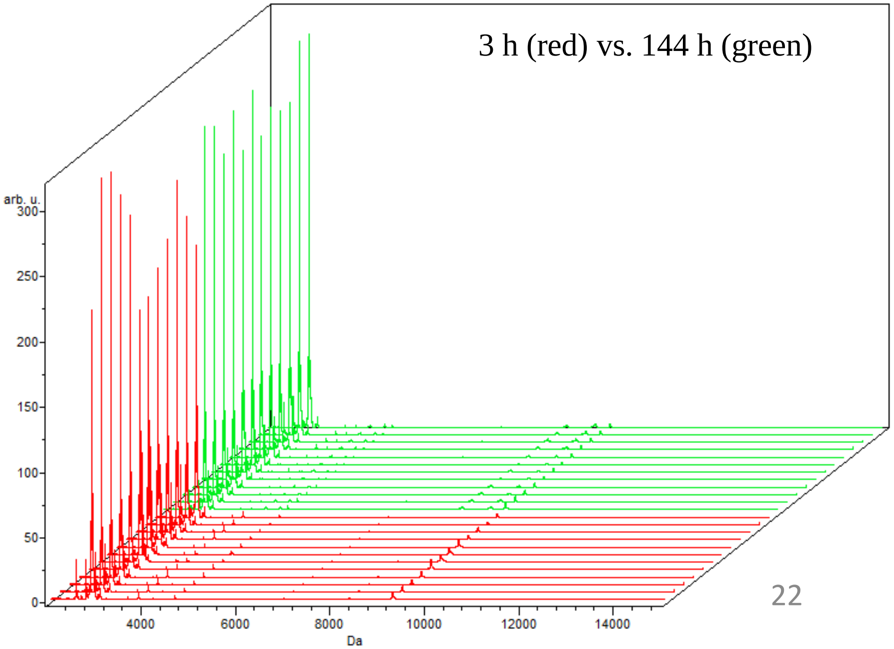
3 h (red) vs. 144 h (green)
arb. u.
Da

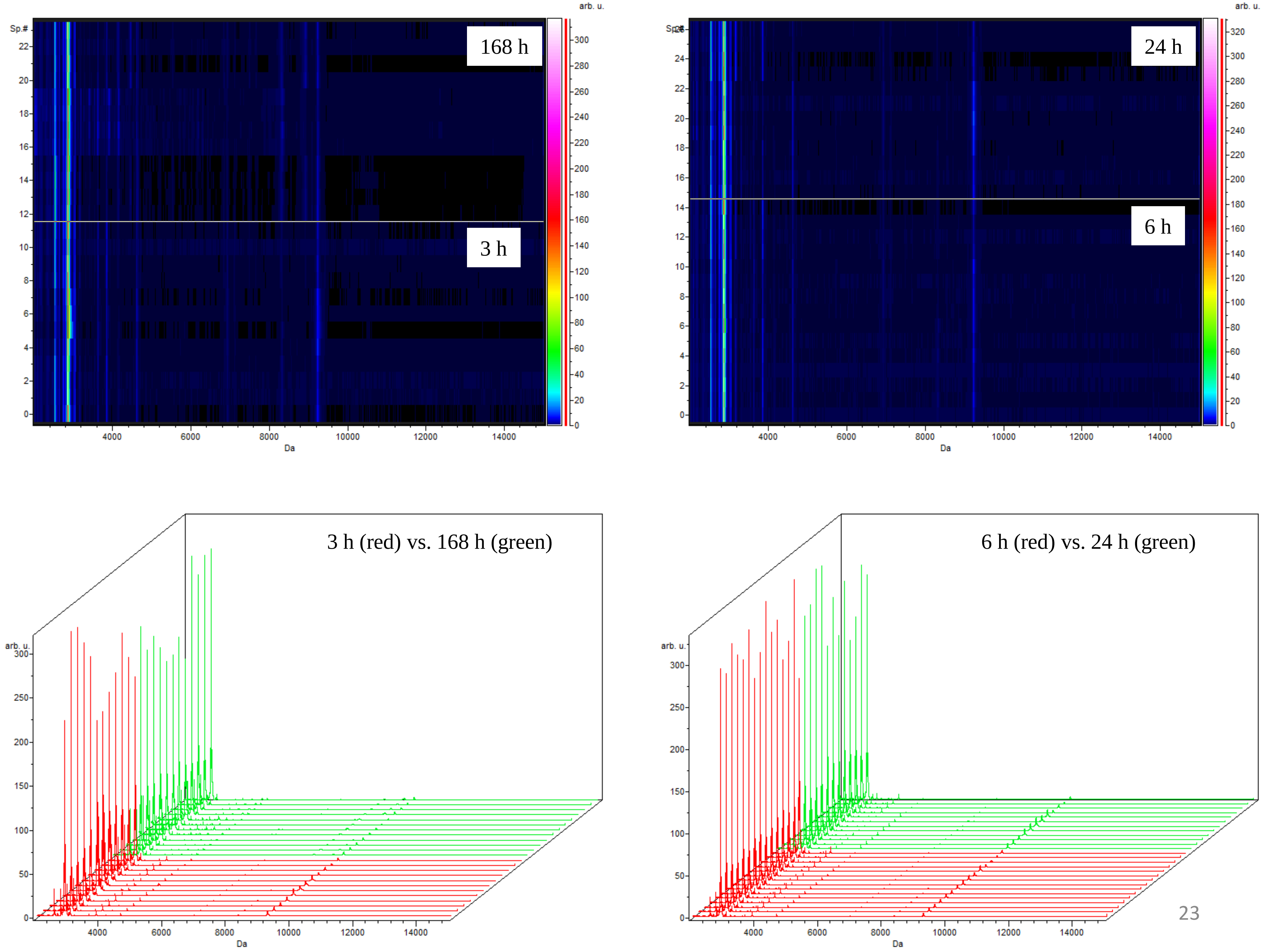
168 h
3 h
24 h
6 h
Sp.#
Da
arb. u.
3 h (red) vs. 168 h (green)
6 h (red) vs. 24 h (green)

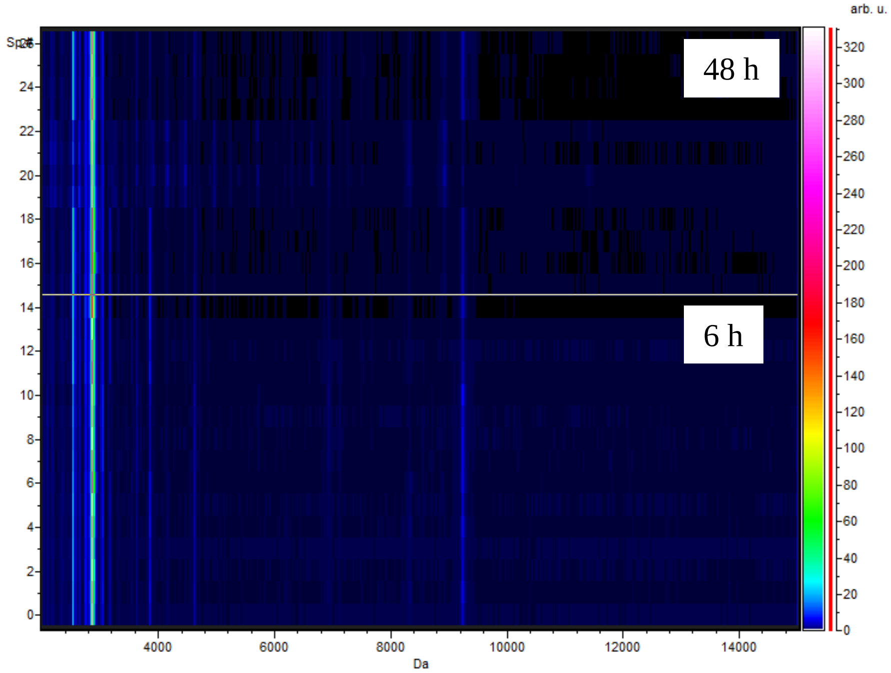
48 h
6 h
Spc#
Da
arb. u.

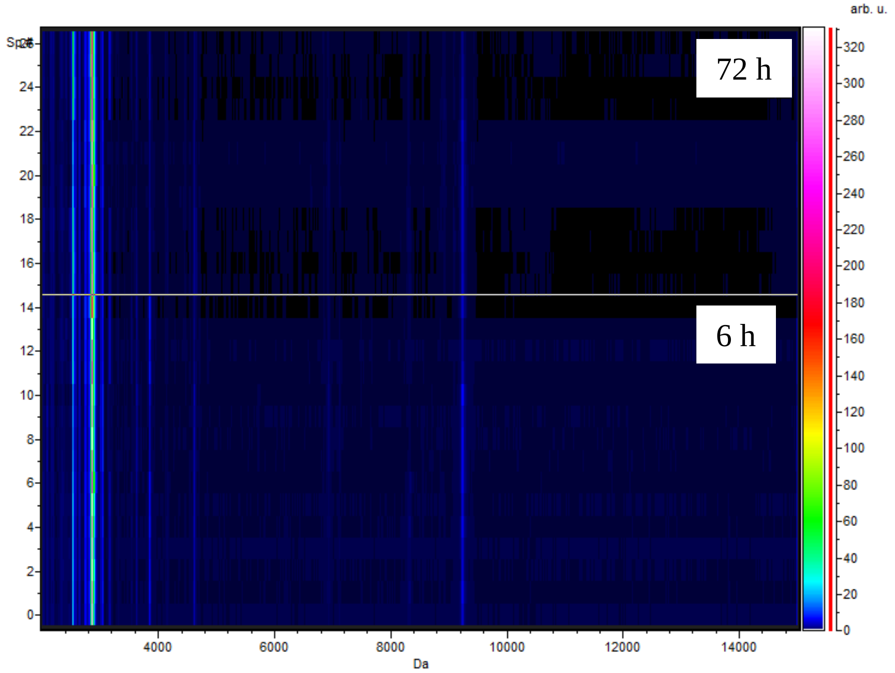
72 h
6 h
Spc#
Da
arb. u.

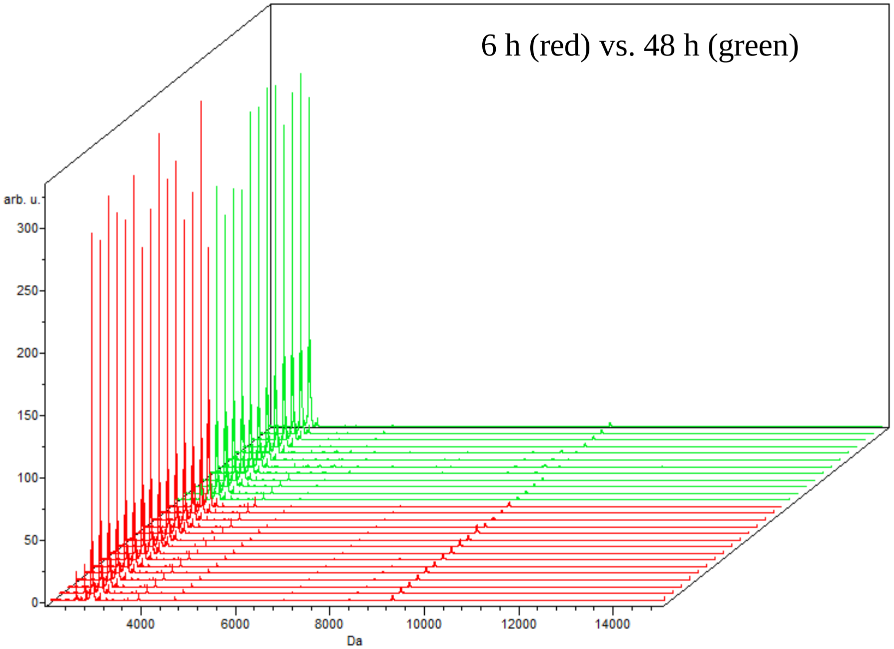
6 h (red) vs. 48 h (green)
arb. u.
Da

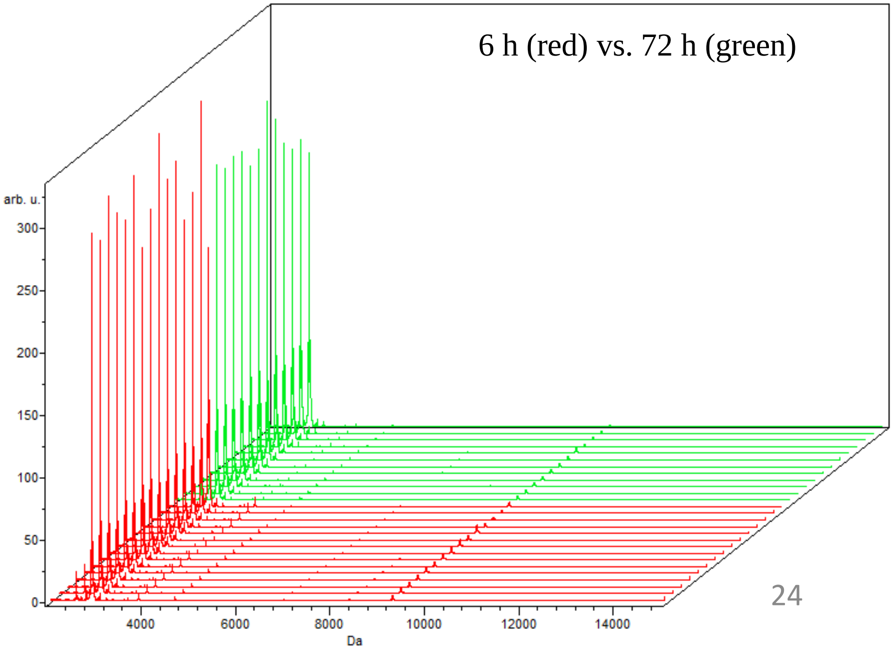
6 h (red) vs. 72 h (green)
arb. u.
Da

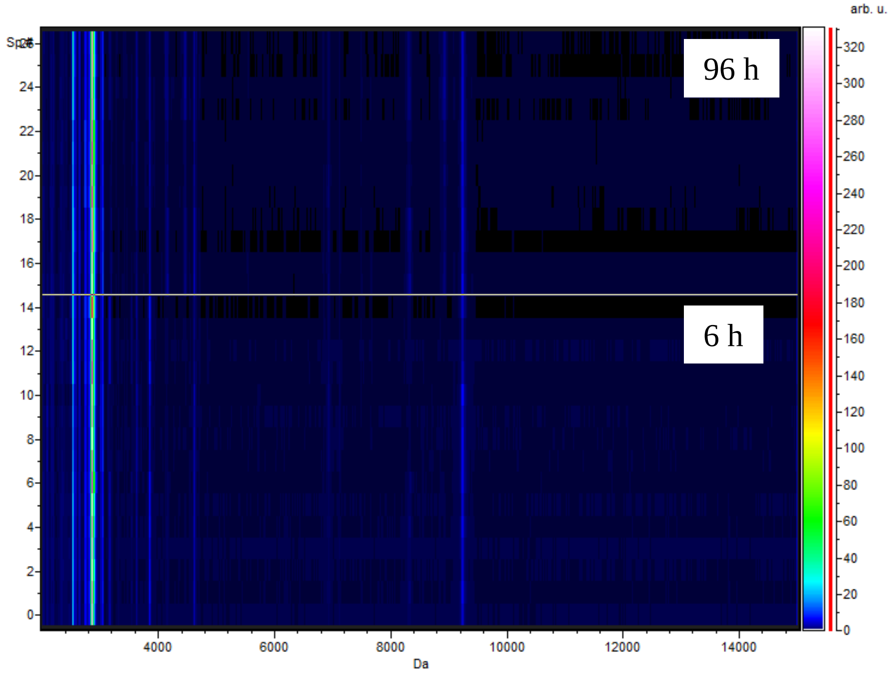
96 h
6 h
arb. u.
Da

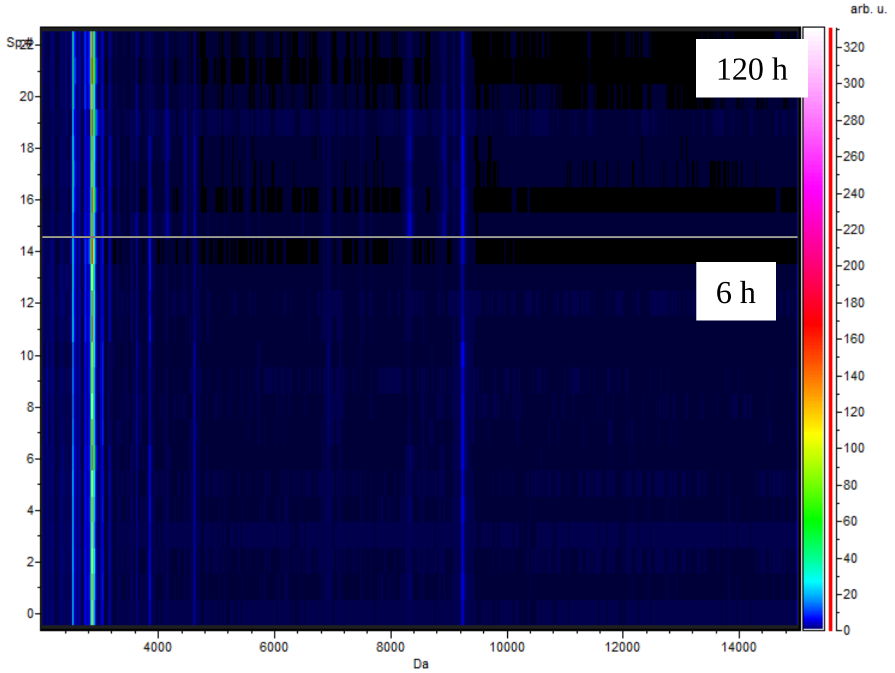
120 h
6 h
arb. u.
Da

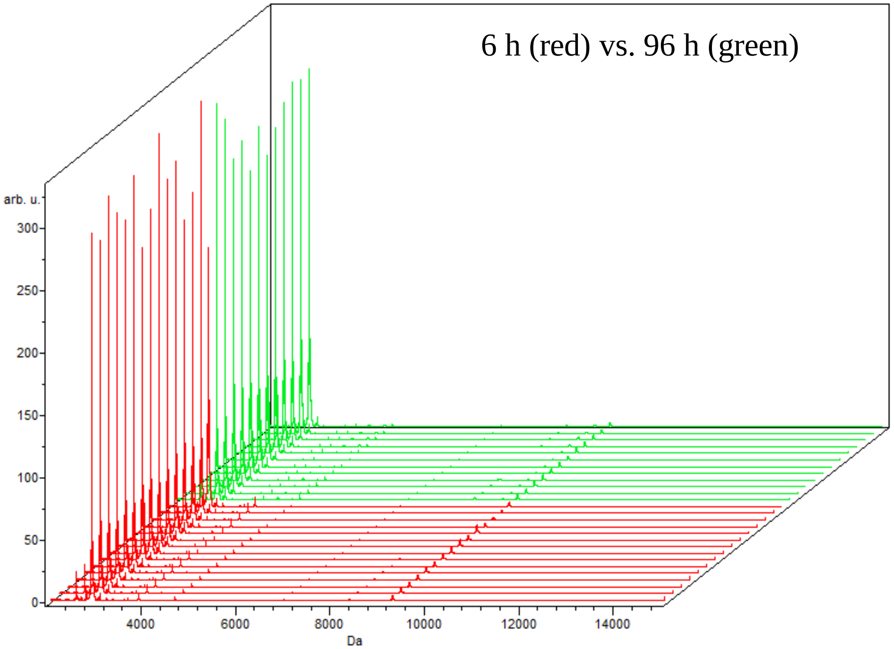
6 h (red) vs. 96 h (green)
arb. u.
Da

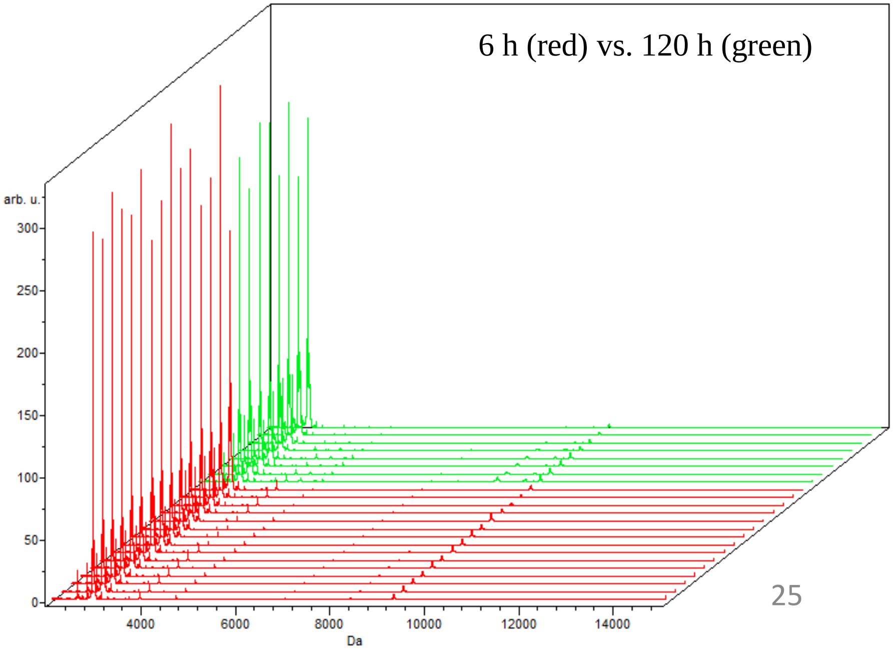
6 h (red) vs. 120 h (green)
arb. u.
Da

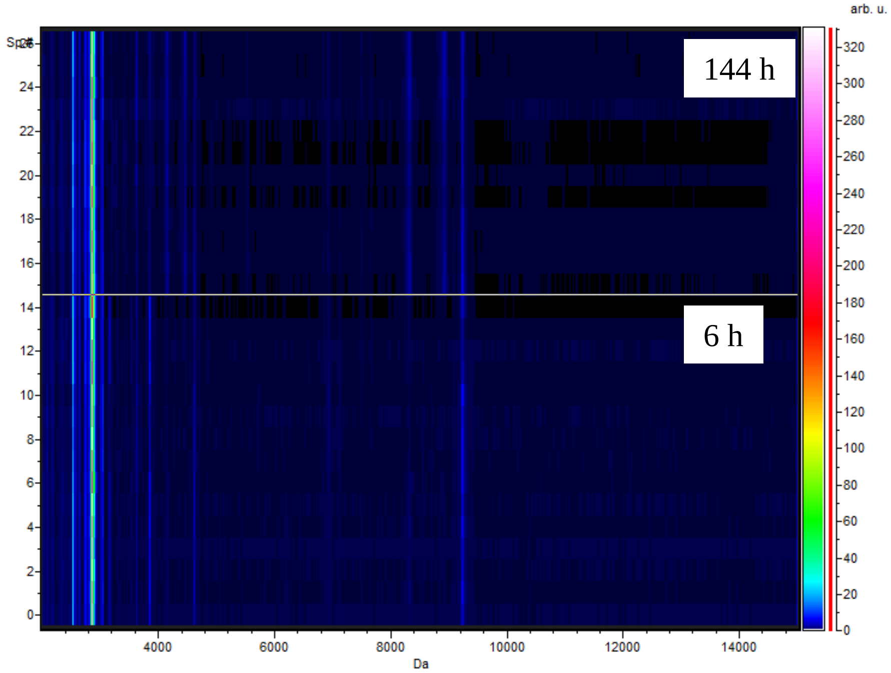
144 h
6 h
arb. u.
Spc#
Da
4000
6000
8000
10000
12000
14000

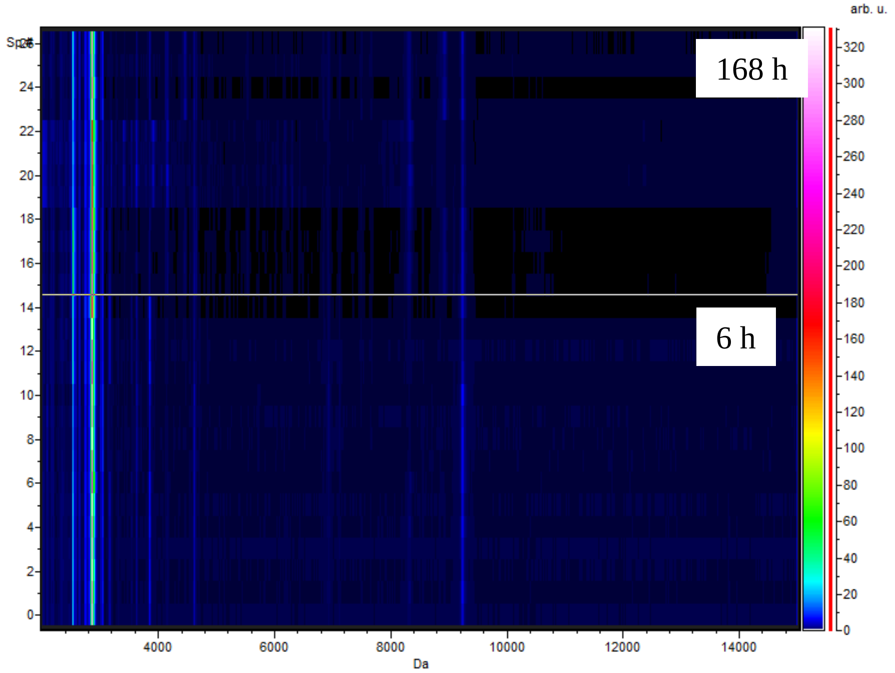
168 h
6 h
arb. u.
Spc#
Da
4000
6000
8000
10000
12000
14000

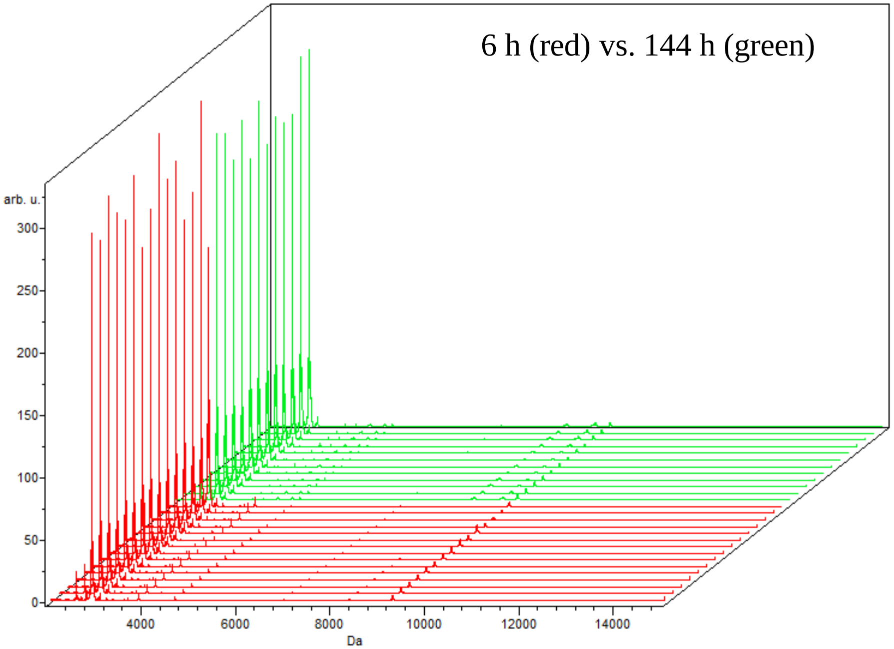
6 h (red) vs. 144 h (green)
arb. u.
Da
4000
6000
8000
10000
12000
14000

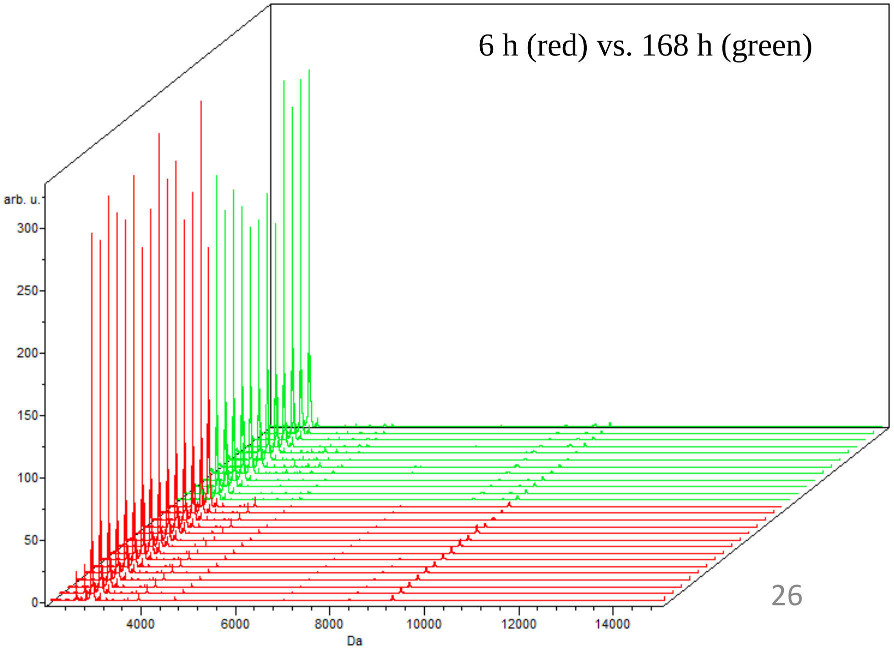
6 h (red) vs. 168 h (green)
arb. u.
Da
4000
6000
8000
10000
12000
14000

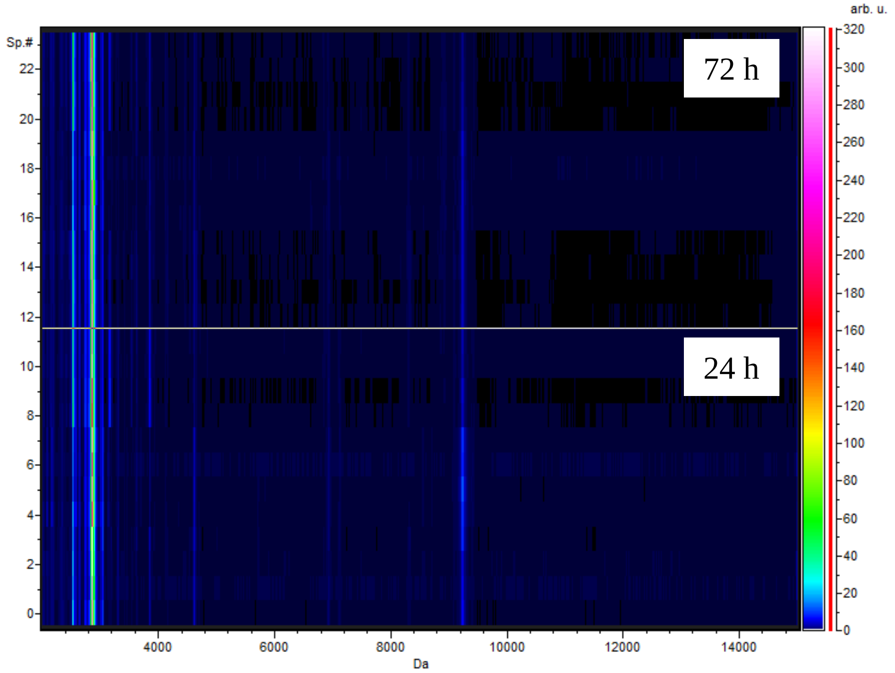
72 h
24 h
Sp.#
Da
arb. u.

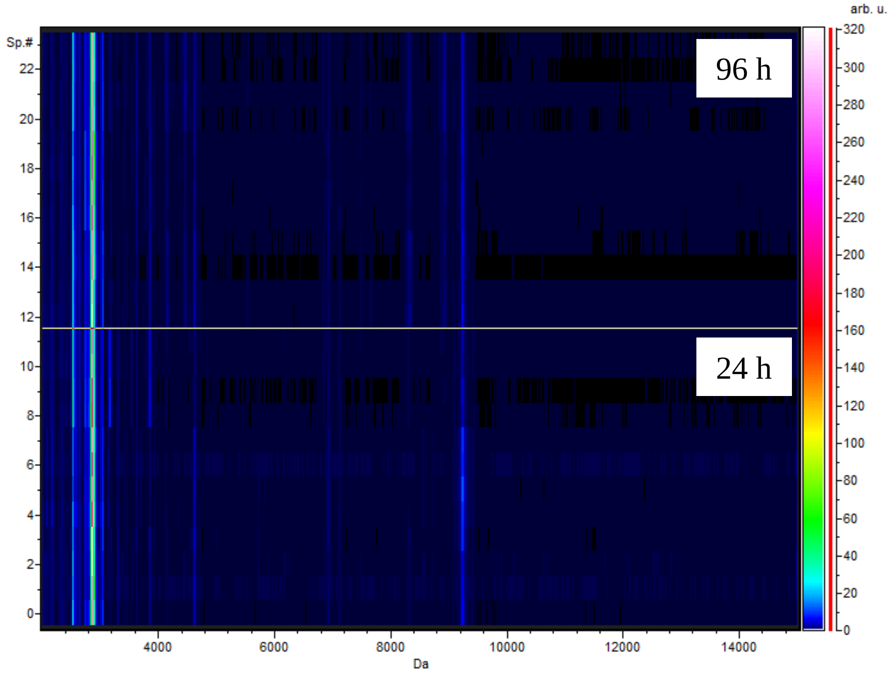
96 h
24 h
Sp.#
Da
arb. u.

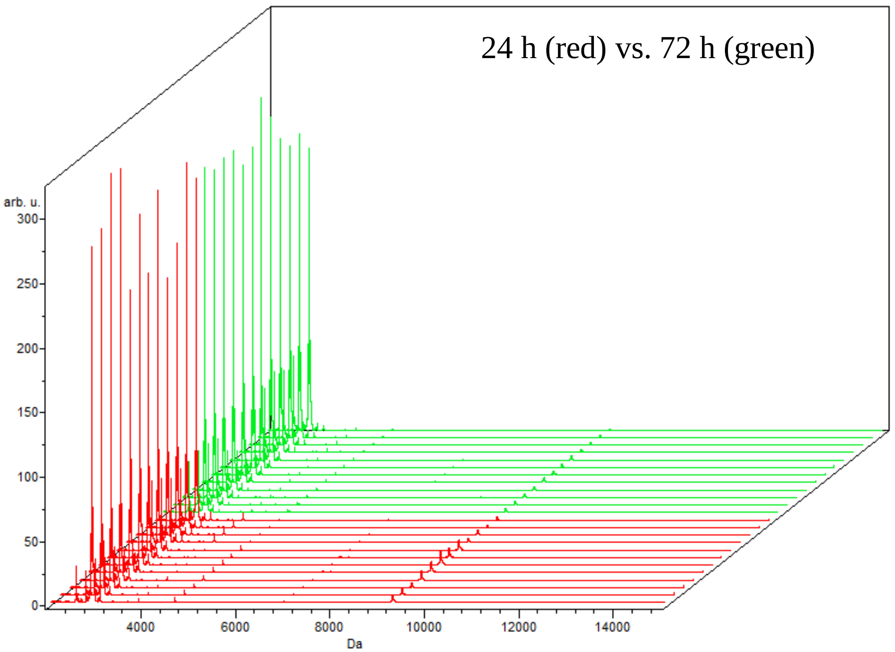
24 h (red) vs. 72 h (green)
arb. u.
Da

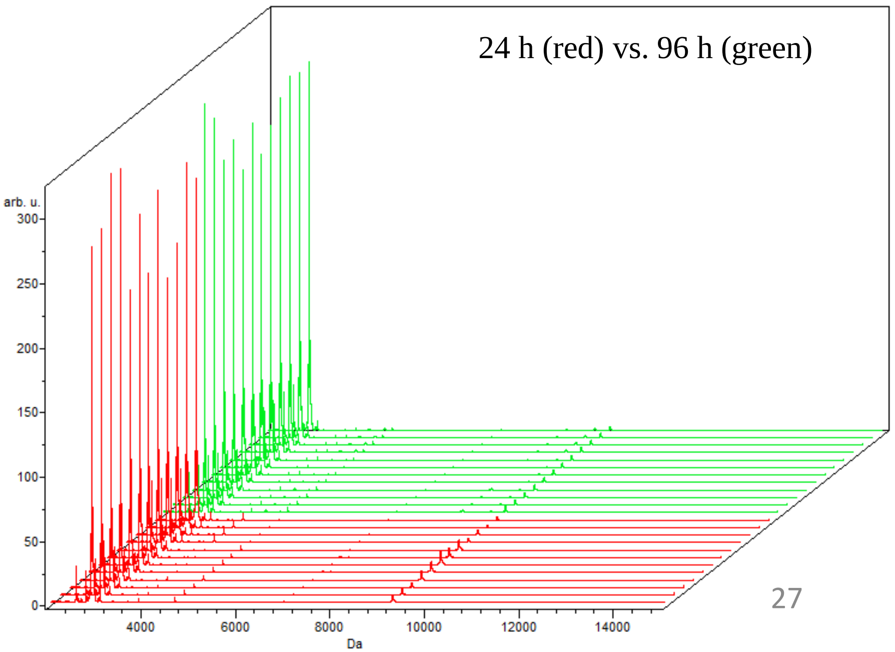
24 h (red) vs. 96 h (green)
arb. u.
Da

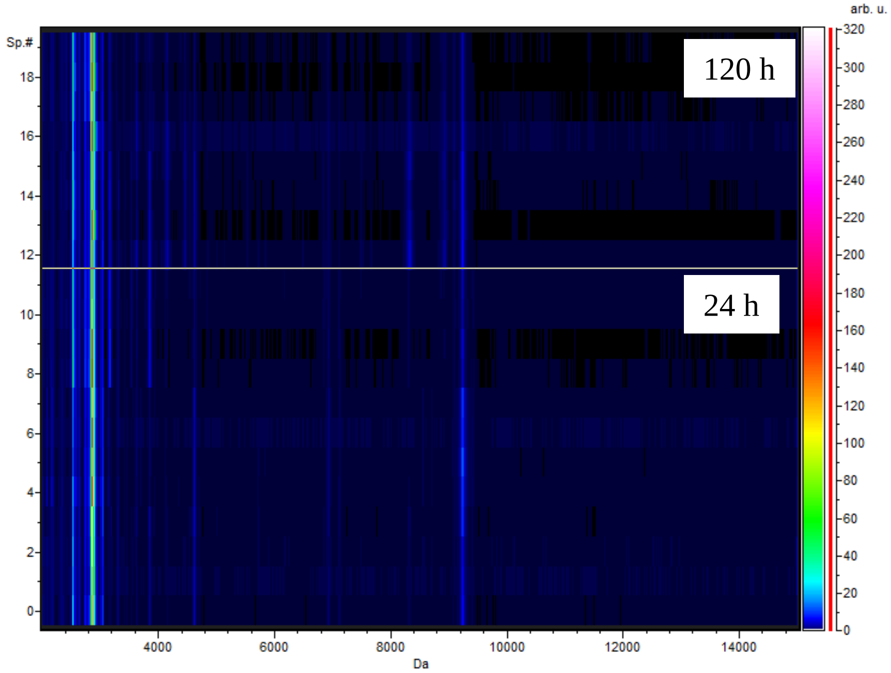
120 h
24 h
Sp.#
Da
arb. u.

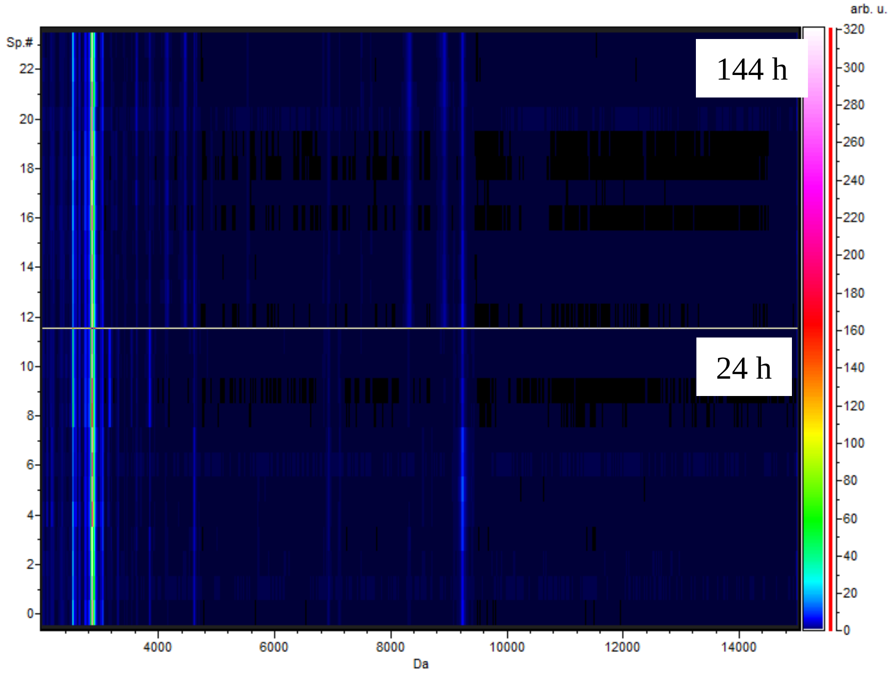
144 h
24 h
Sp.#
Da
arb. u.

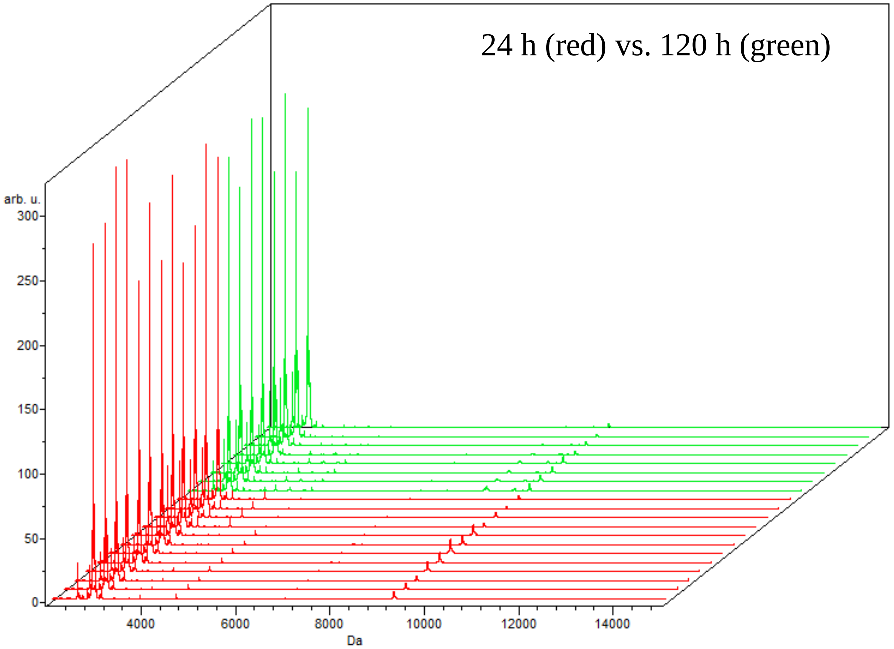
24 h (red) vs. 120 h (green)
arb. u.
Da

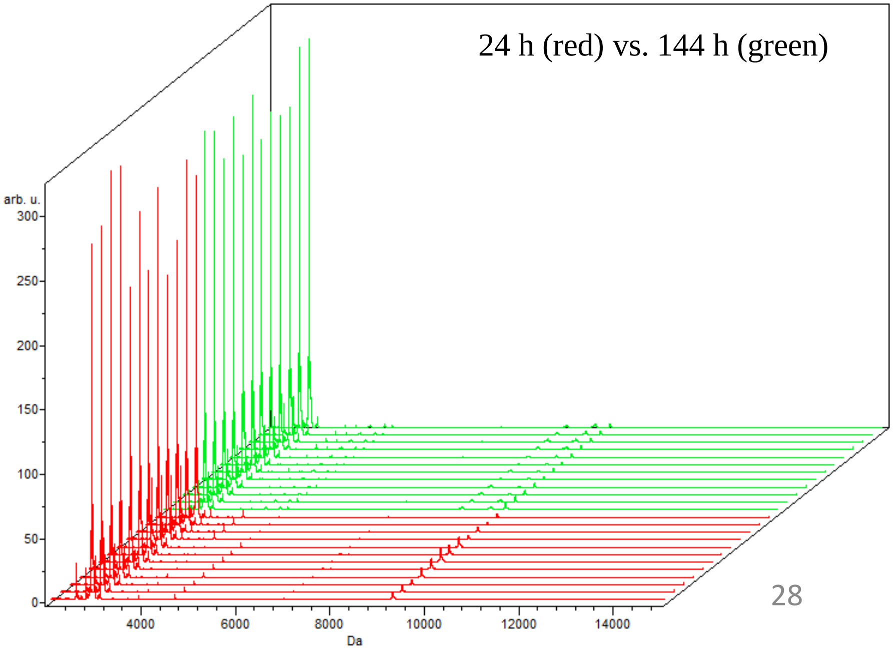
24 h (red) vs. 144 h (green)
arb. u.
Da

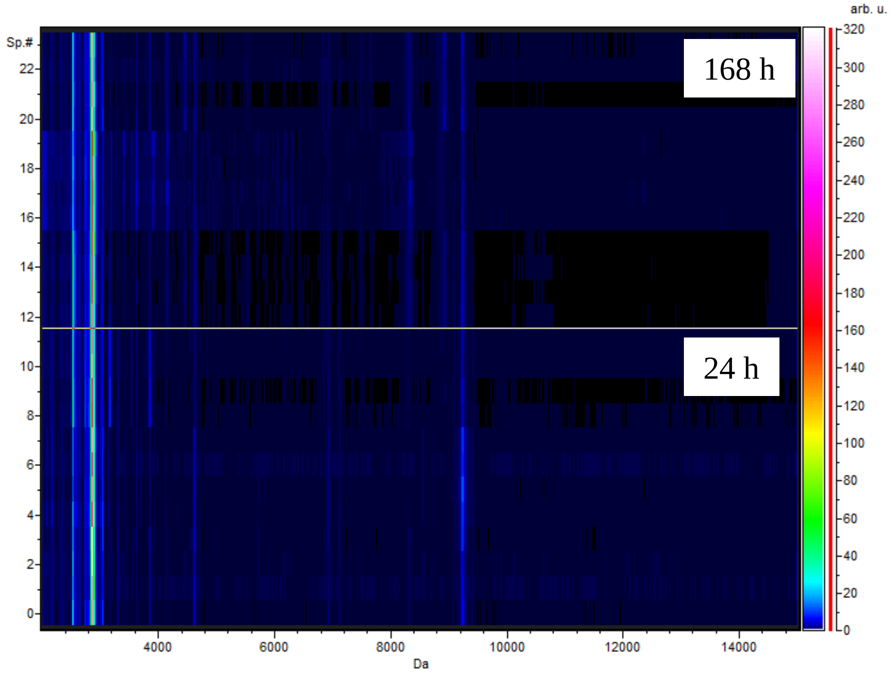
arb. u.
Sp.#
168 h
24 h
Da

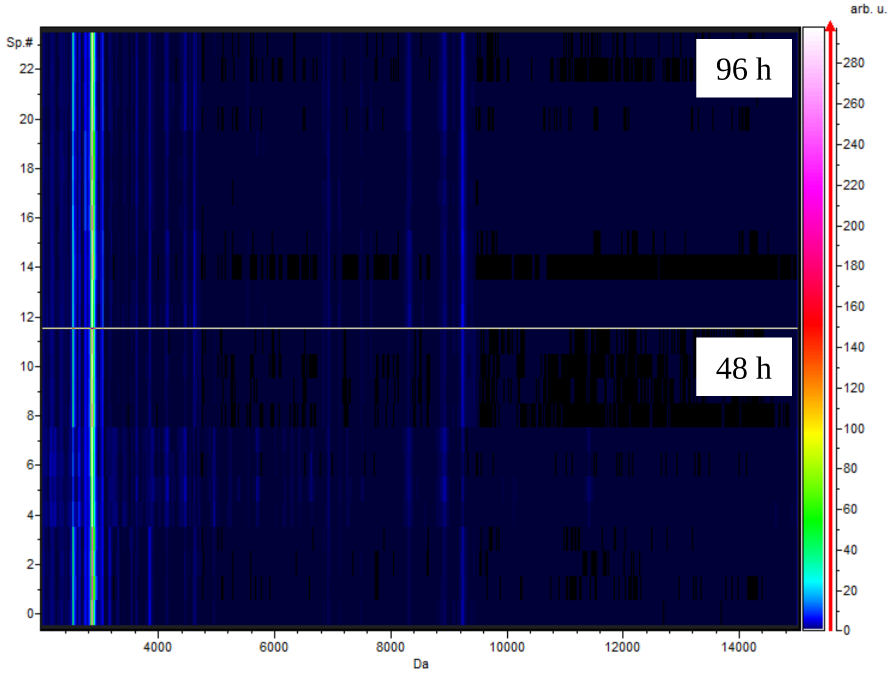
arb. u.
Sp.#
96 h
48 h
Da

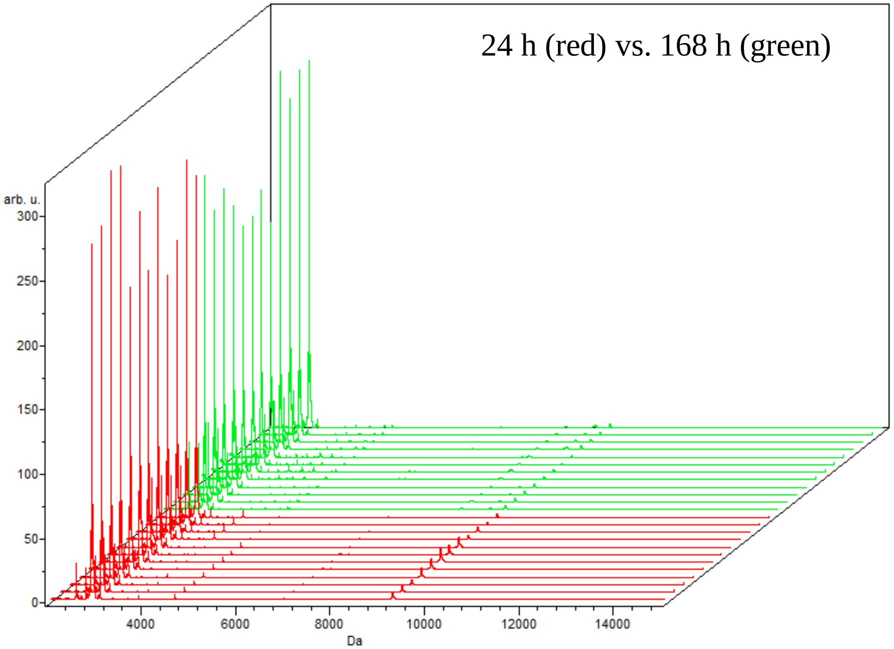
24 h (red) vs. 168 h (green)
arb. u.
Da

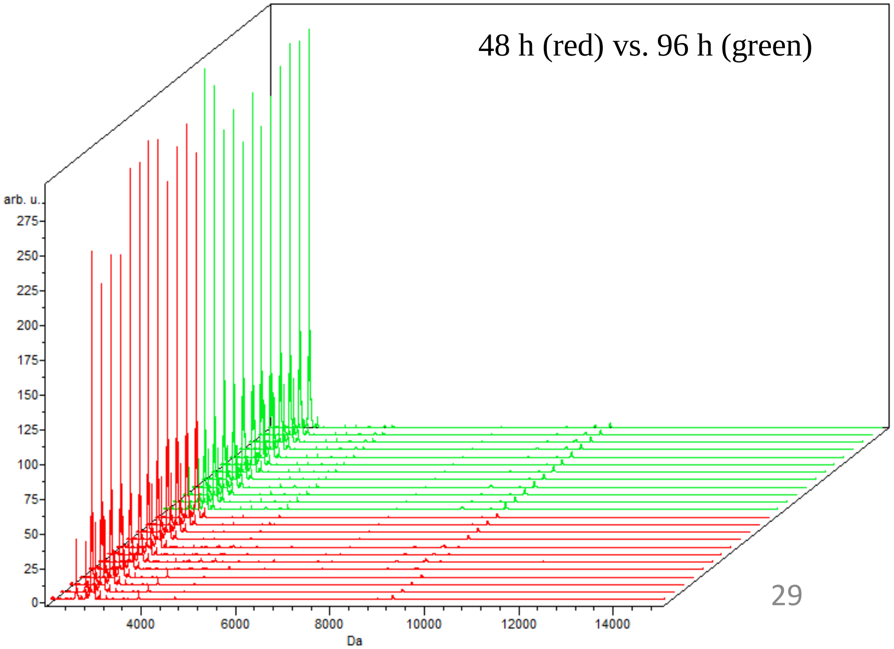
48 h (red) vs. 96 h (green)
arb. u.
Da

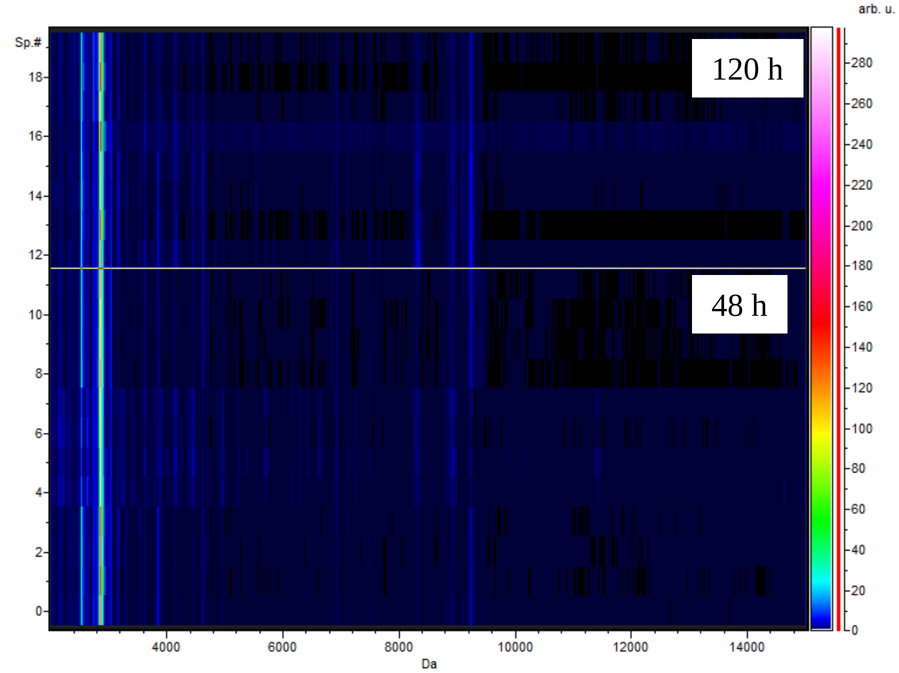
120 h
48 h
Sp.#
Da
arb. u.

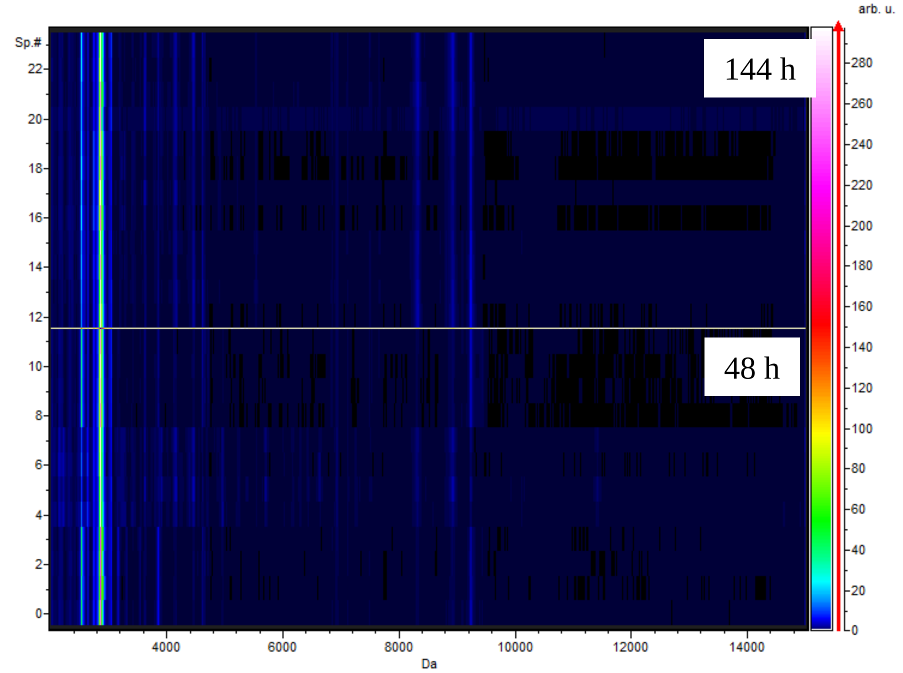
144 h
48 h
Sp.#
Da
arb. u.

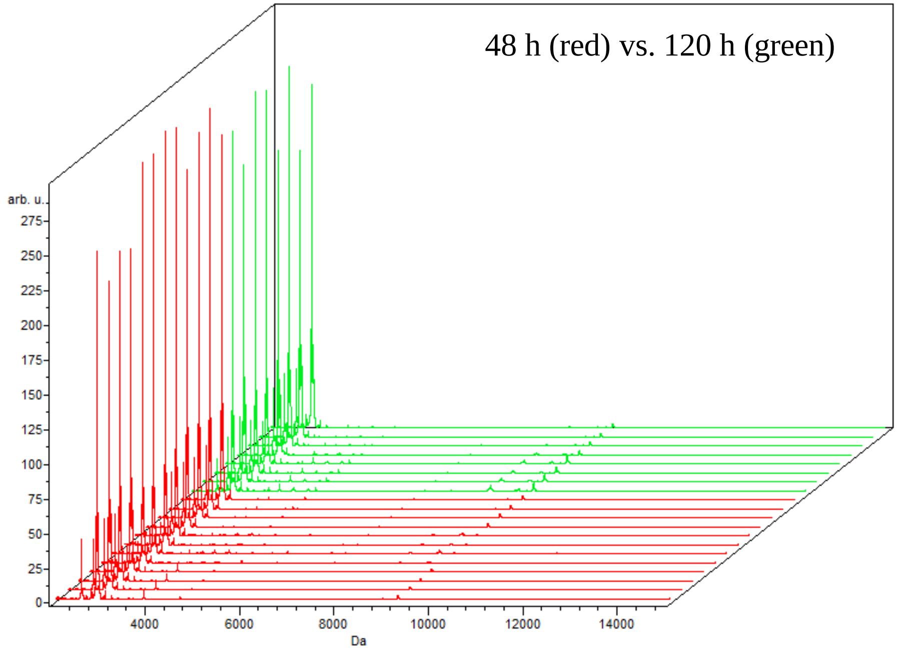
48 h (red) vs. 120 h (green)
arb. u.
Da

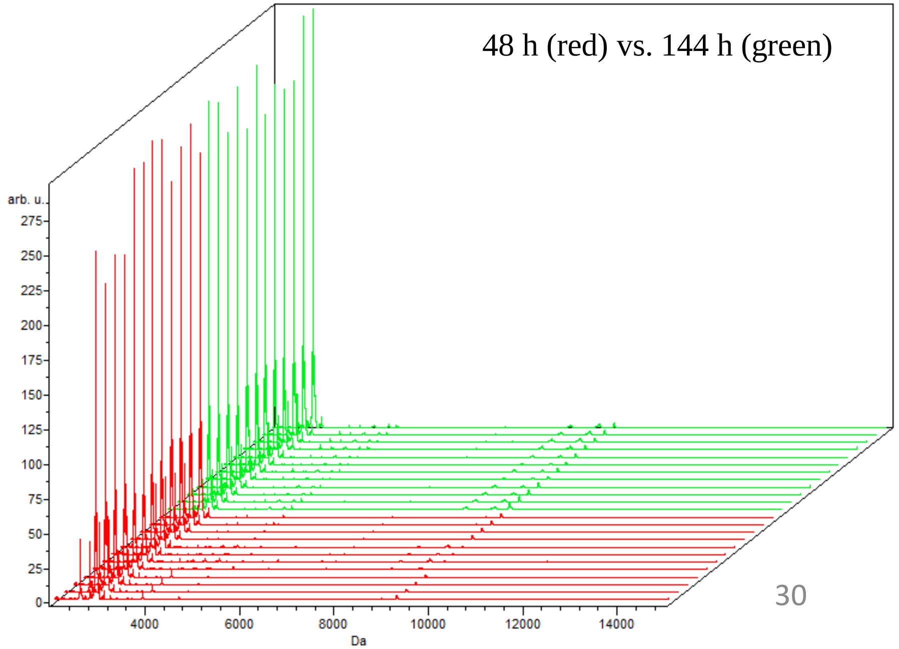
48 h (red) vs. 144 h (green)
arb. u.
Da

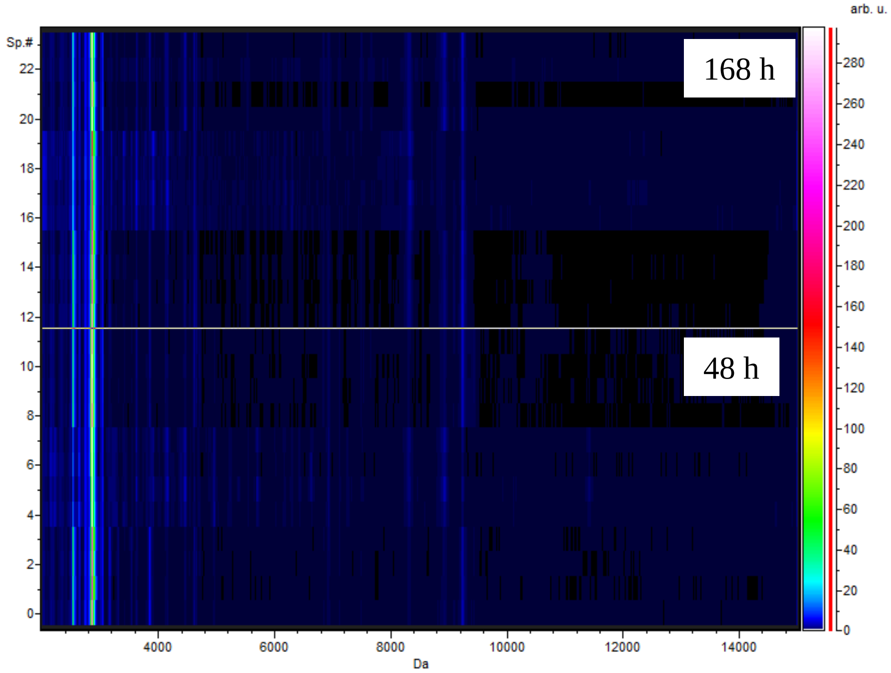
arb. u.
Sp.#
168 h
48 h
Da

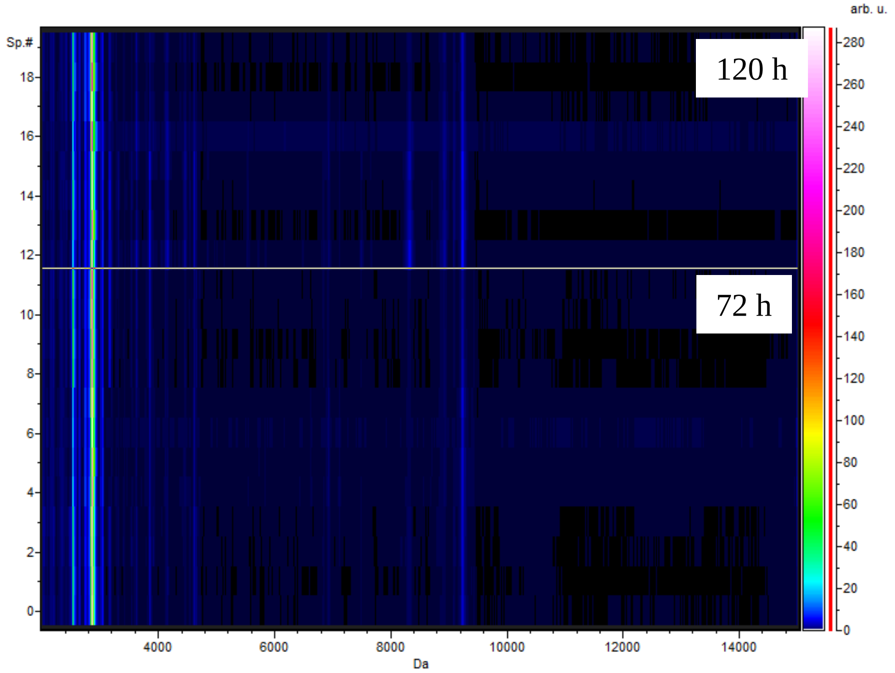
arb. u.
Sp.#
120 h
72 h
Da

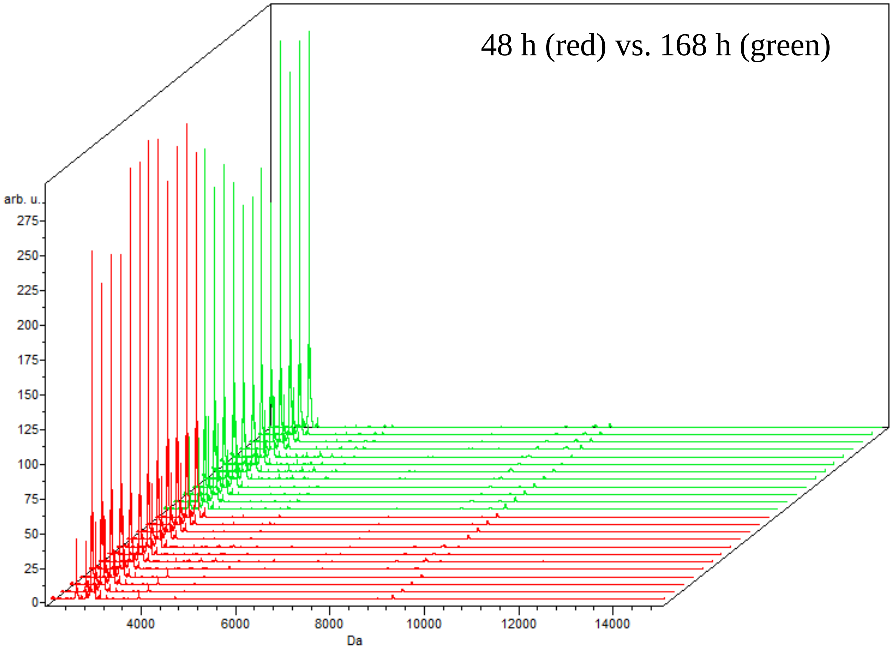
48 h (red) vs. 168 h (green)
arb. u.
Da

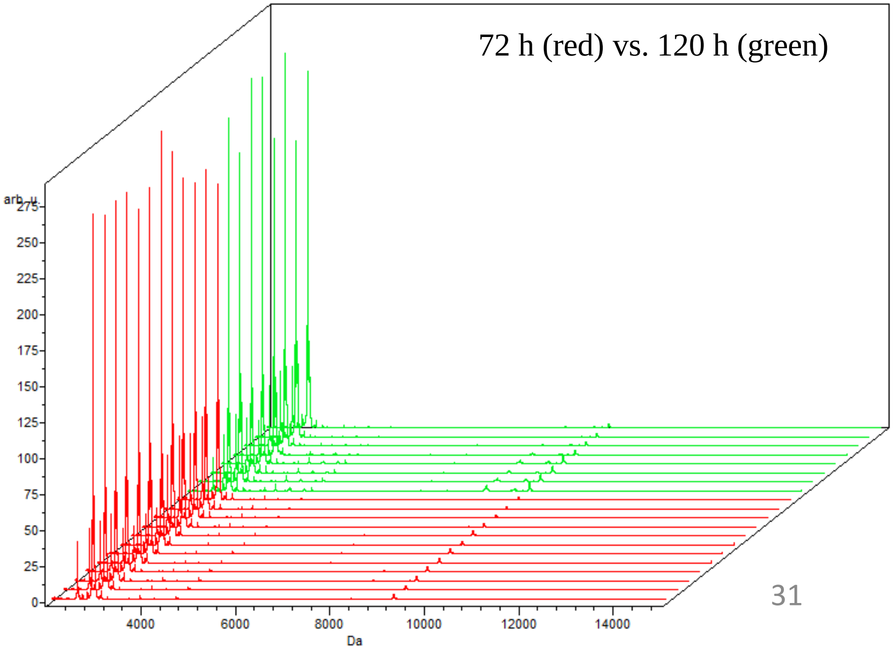
72 h (red) vs. 120 h (green)
arb. u.
Da

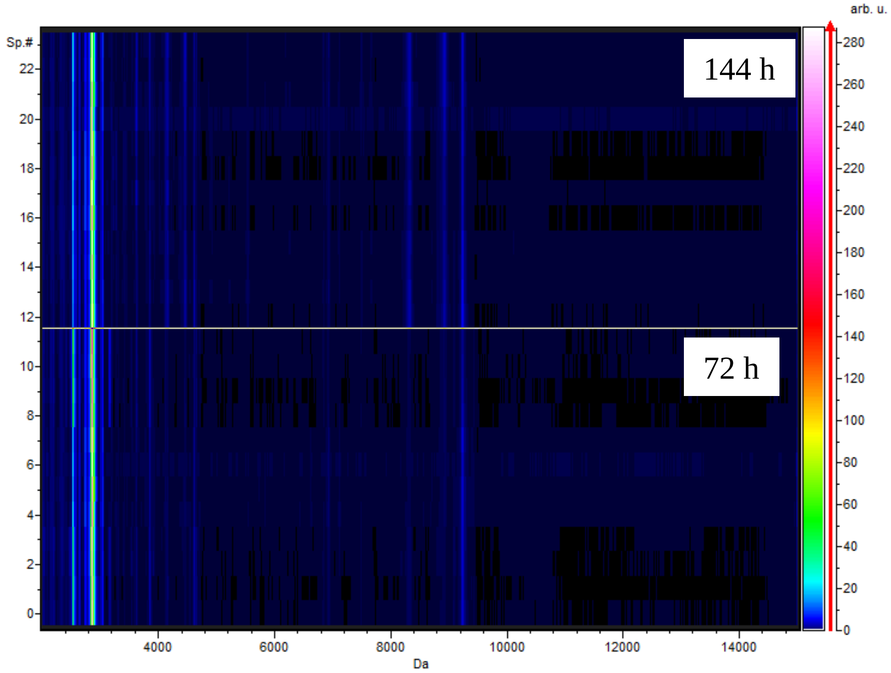
144 h
72 h
Sp.#
arb. u.
Da

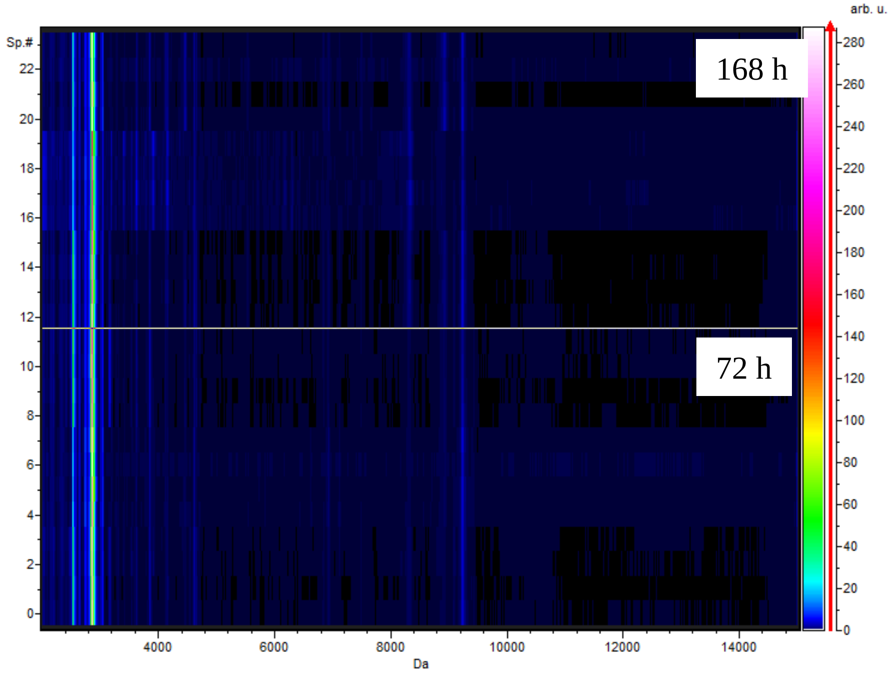
168 h
72 h
Sp.#
arb. u.
Da

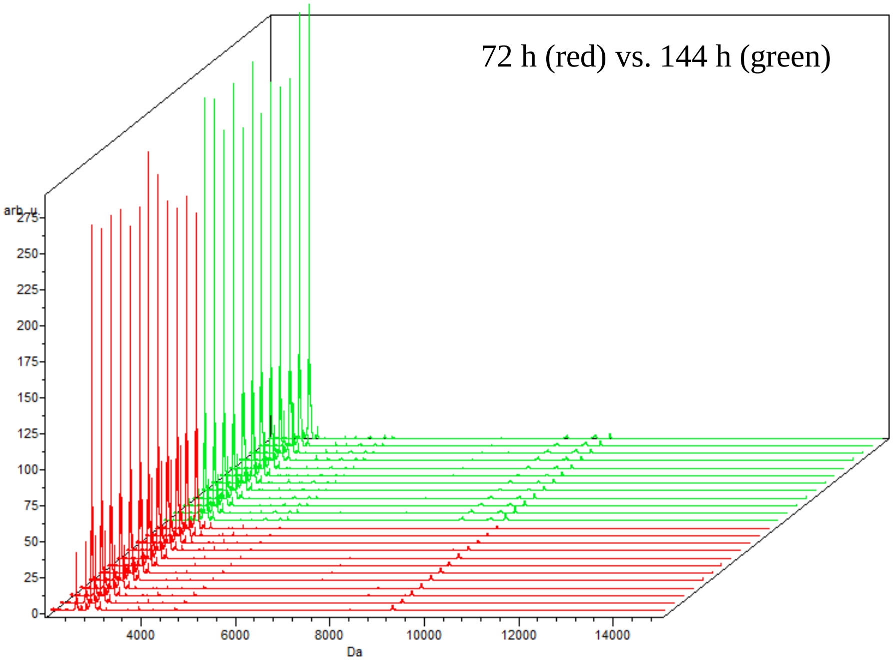
72 h (red) vs. 144 h (green)
arb. u.
Da

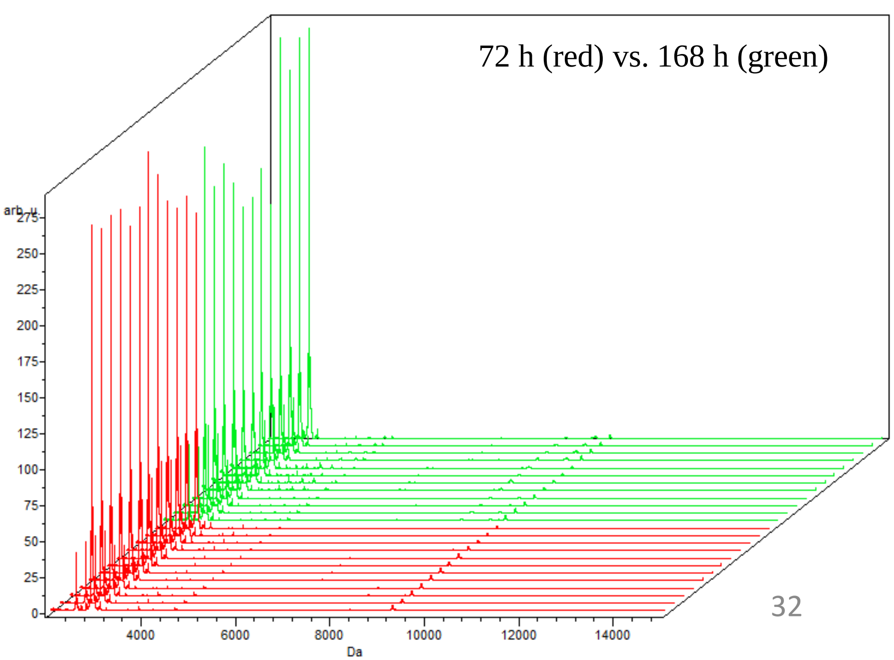
72 h (red) vs. 168 h (green)
arb. u.
Da

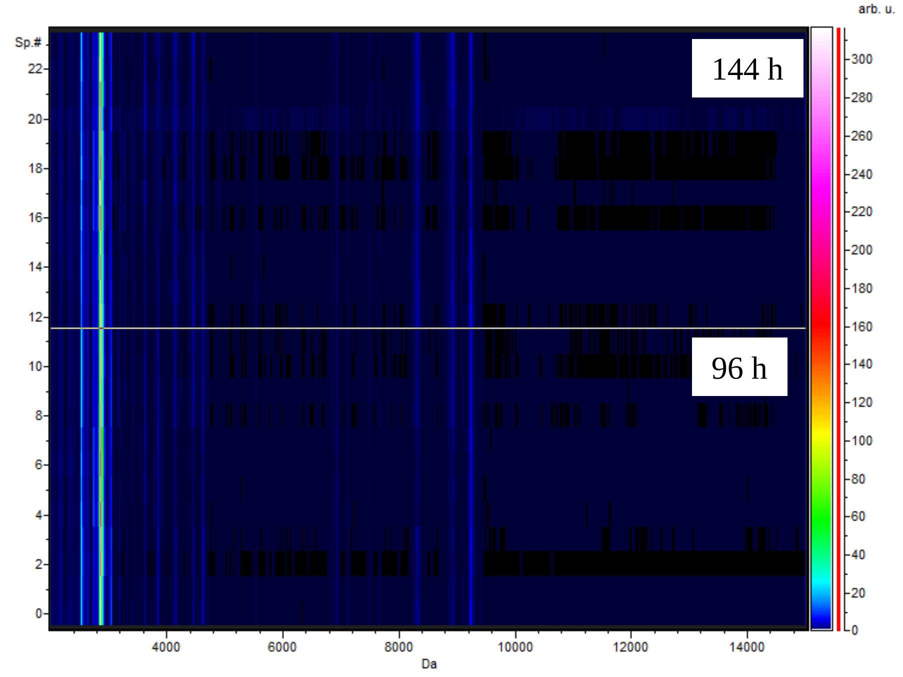
144 h
96 h
arb. u.
Sp.#
Da

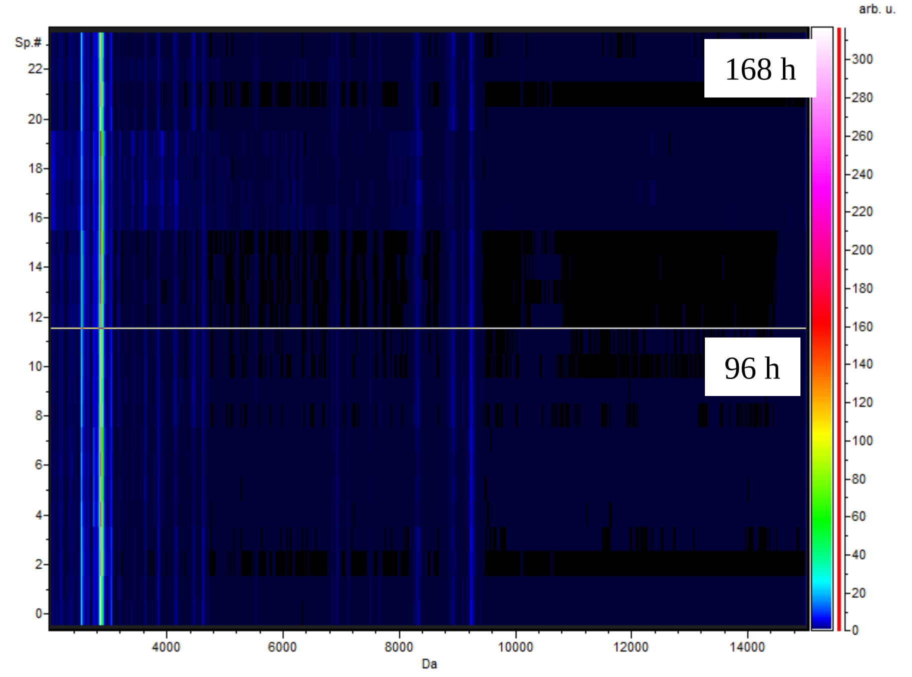
168 h
96 h
arb. u.
Sp.#
Da

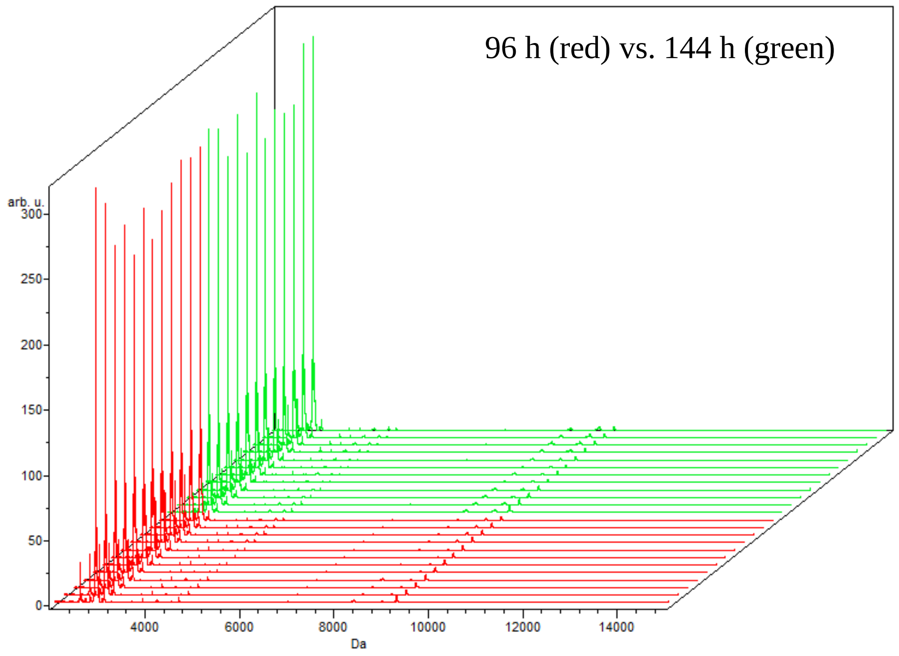
96 h (red) vs. 144 h (green)
arb. u.
Da

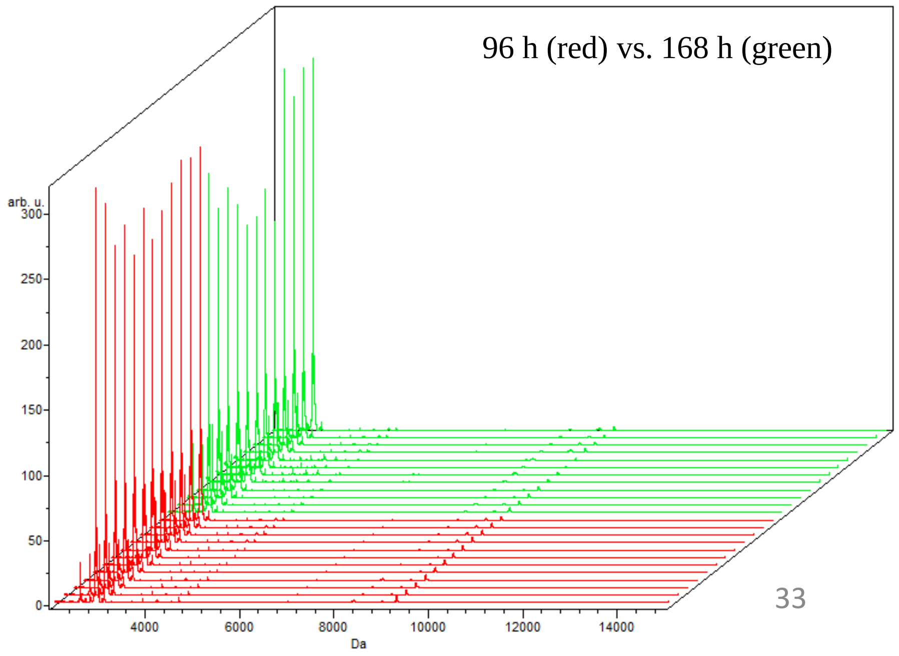
96 h (red) vs. 168 h (green)
arb. u.
Da

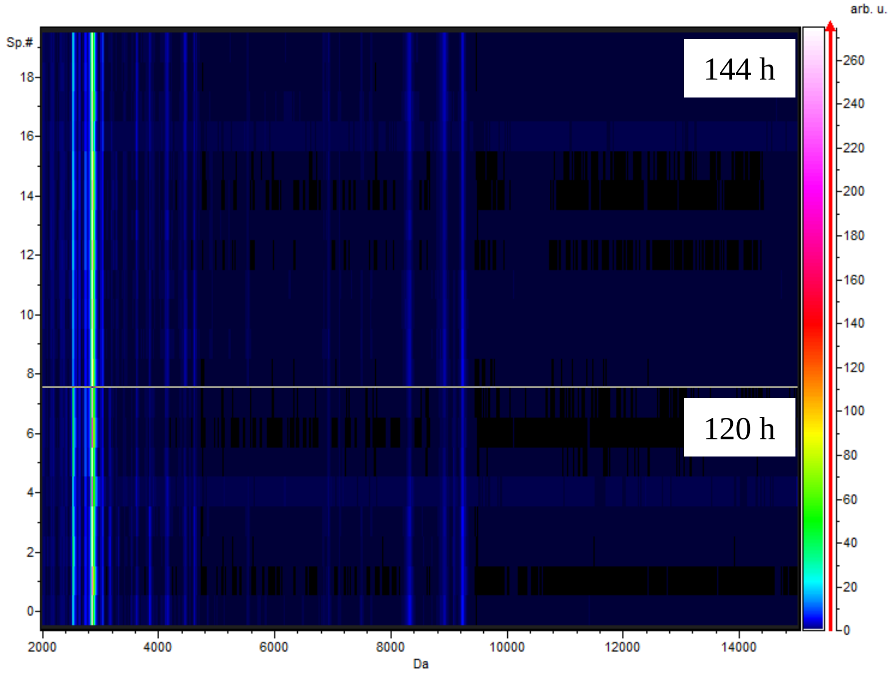
144 h
120 h
Sp.#
Da
arb. u.

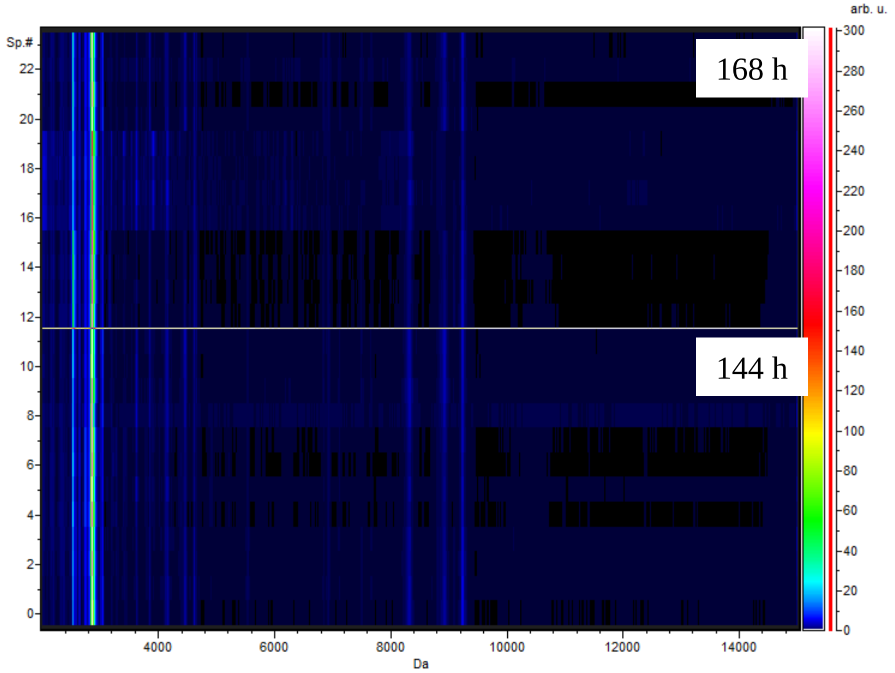
168 h
144 h
Sp.#
Da
arb. u.

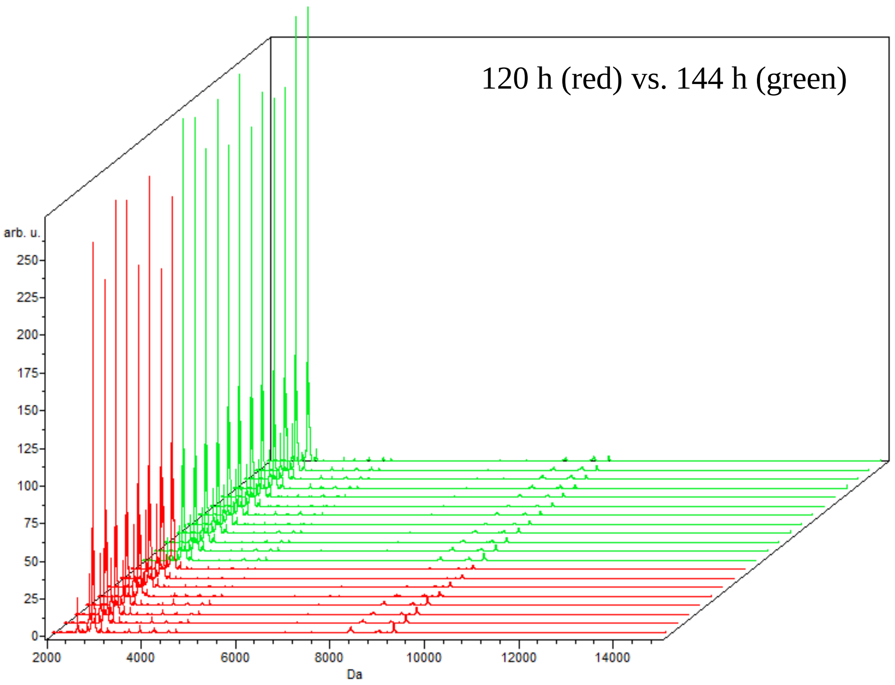
120 h (red) vs. 144 h (green)
arb. u.
Da

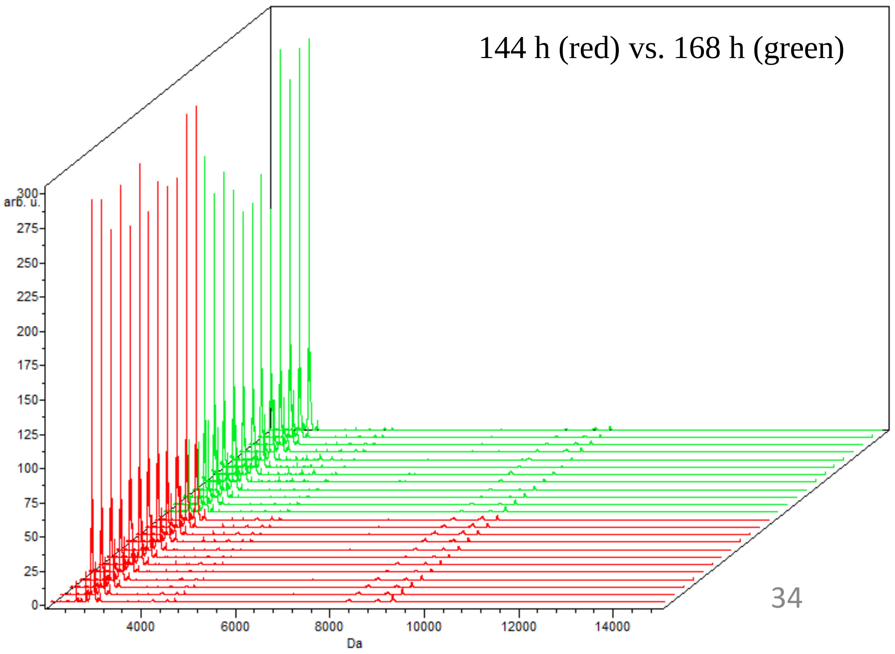
144 h (red) vs. 168 h (green)
arb. u.
Da

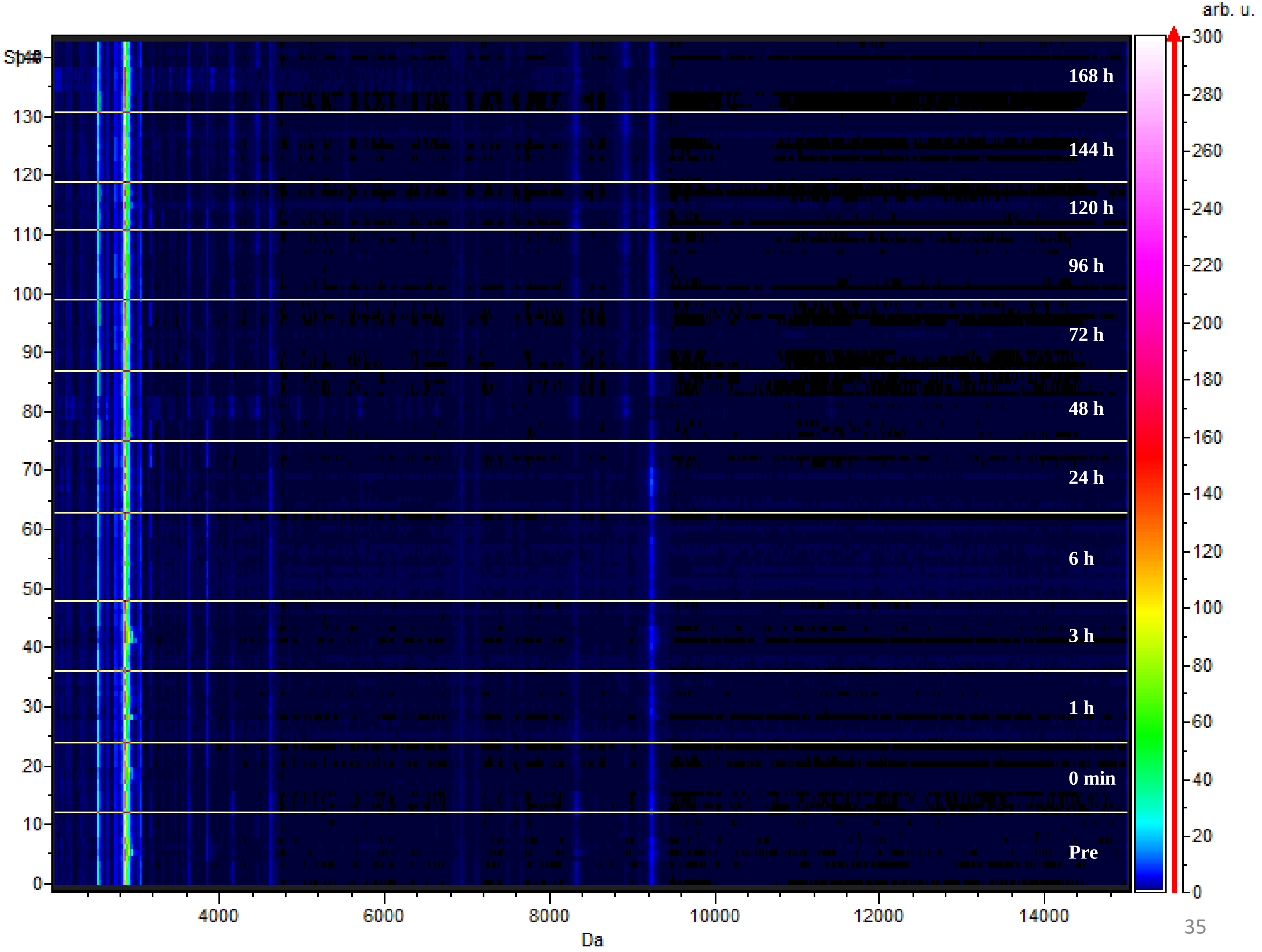
arb. u.
Spec #
168 h
144 h
120 h
96 h
72 h
48 h
24 h
6 h
3 h
1 h
0 min
Pre
Da
4000
6000
8000
10000
12000
14000
0
10
20
30
40
50
60
70
80
90
100
110
120
130
140
300
280
260
240
220
200
180
160
140
120
100
80
60
40
20
0

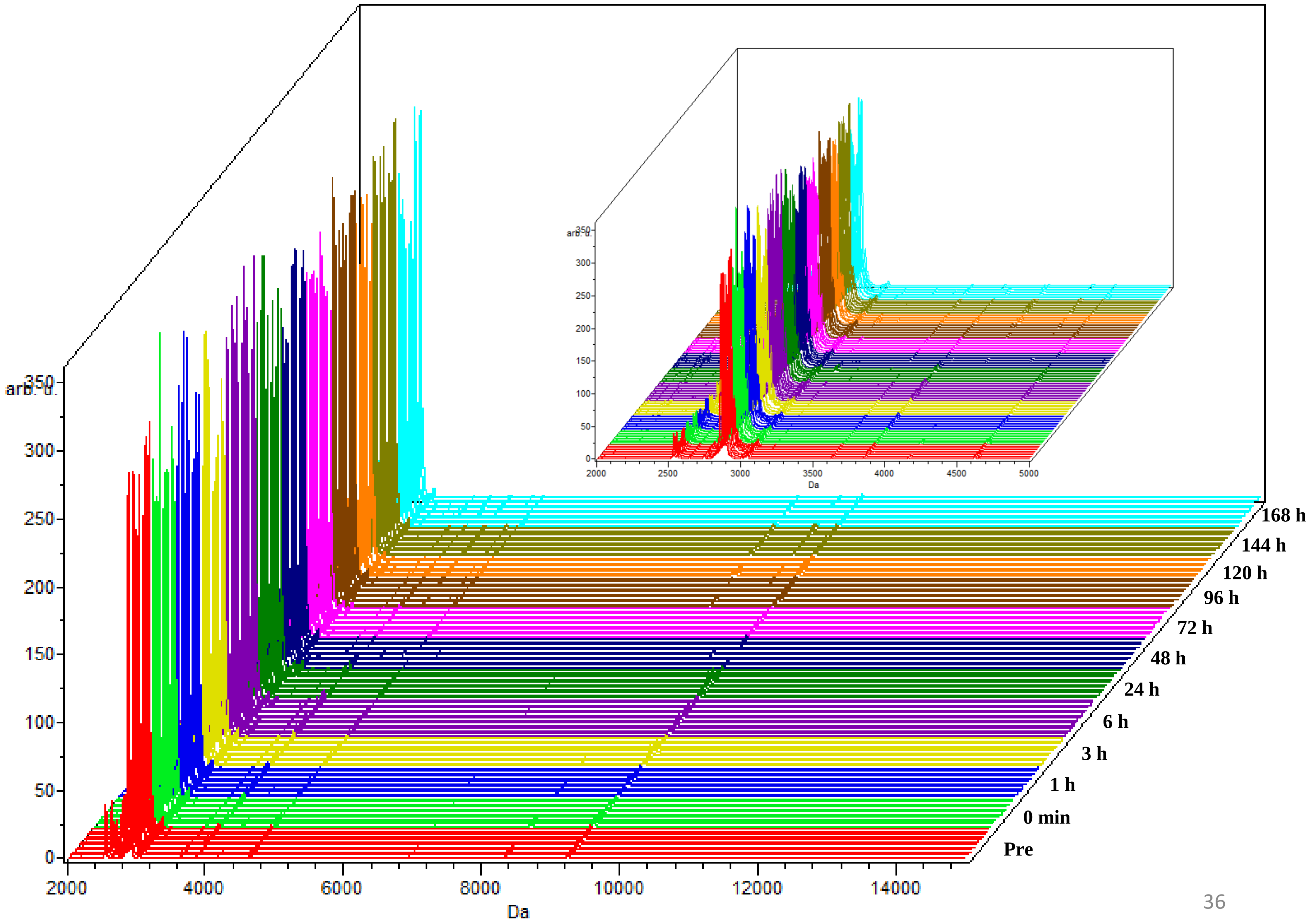

arb. u.
350
300
250
200
150
100
50
0
2000
4000
6000
8000
10000
12000
14000
Da
168 h
144 h
120 h
96 h
72 h
48 h
24 h
6 h
3 h
1 h
0 min
Pre
arb. u.
350
300
250
200
150
100
50
0
2000
2500
3000
3500
4000
4500
5000
Da
