# Supplementary material for: Chronological Profiling of Plasma Native Peptides after Hepatectomy in Pigs: Toward the Discovery of Human Biomarkers for Liver Regeneration
Source: PLoS One. 2017 Jan 6;12(1):e0167647. doi: 10.1371/journal.pone.0167647 (PMC5218562; doi:10.1371/journal.pone.0167647)
Supplement: S2 Fig — (Page 2~9) serum albumin, (Page 10~14) hemoglobin subunit α, (Page 15~17) basic proline-rich protein, (Page 18~19) histone H1.2, (Page 20) histone H2A1, (Page 21~22) histone H2B1, (Page 23~24) histone H3.1, (Page 25) histone H4, (Page 26~27) non-histone chromosomal protein HMG-17, (Page 28) serotransferrin, (Page 29) apolipoprotein C-III, (Page 30) ryanodine receptor I, and (Page 31) vasodilator-stimulated phosphoprotein. (PPTX) [file pone.0167647.s002.pptx]

## Slide 1
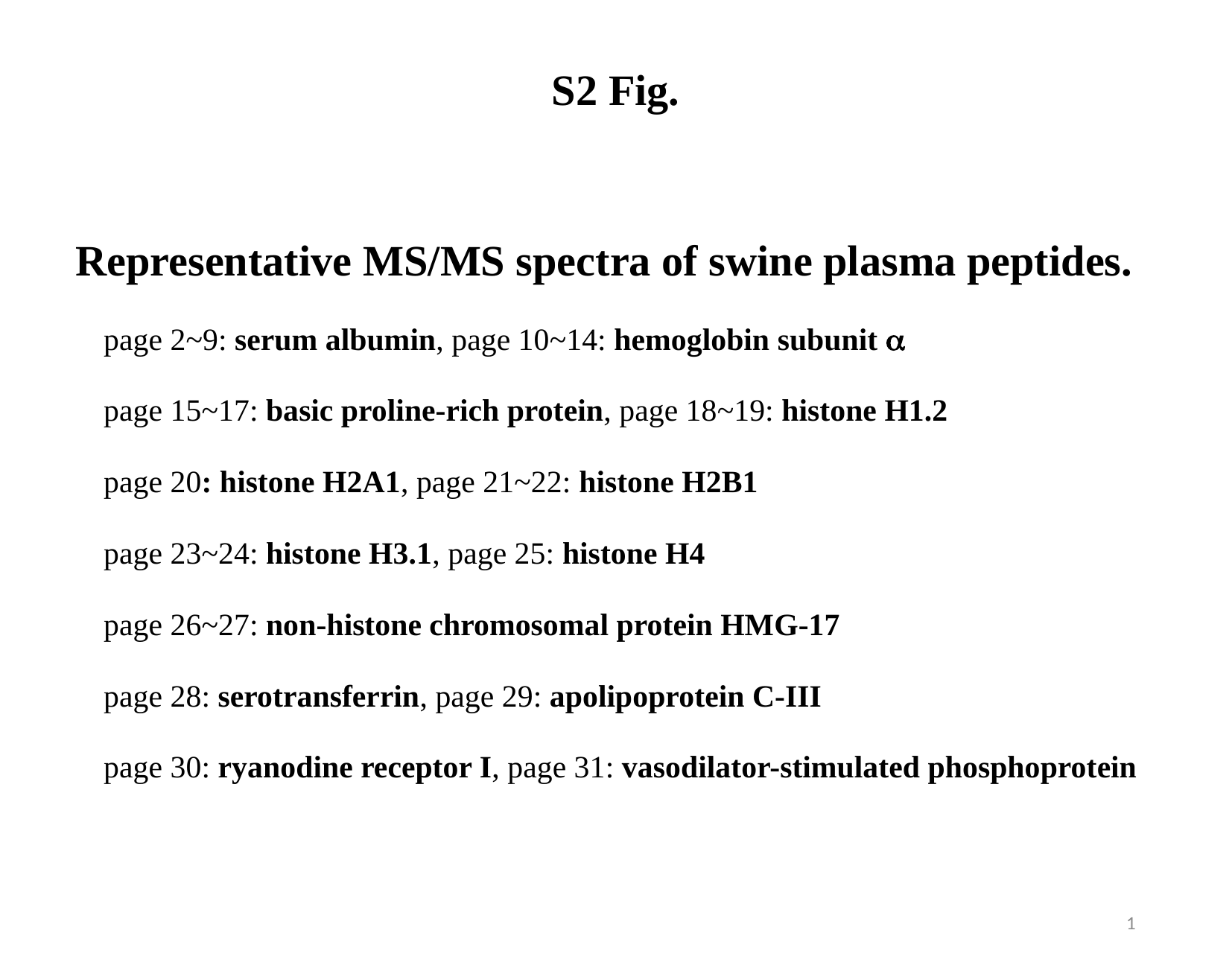

# S2 Fig.
Representative MS/MS spectra of swine plasma peptides.
	page 2~9: serum albumin, page 10~14: hemoglobin subunit a
	page 15~17: basic proline-rich protein, page 18~19: histone H1.2
	page 20: histone H2A1, page 21~22: histone H2B1
	page 23~24: histone H3.1, page 25: histone H4
	page 26~27: non-histone chromosomal protein HMG-17
	page 28: serotransferrin, page 29: apolipoprotein C-III
	page 30: ryanodine receptor I, page 31: vasodilator-stimulated phosphoprotein
1

## Slide 2
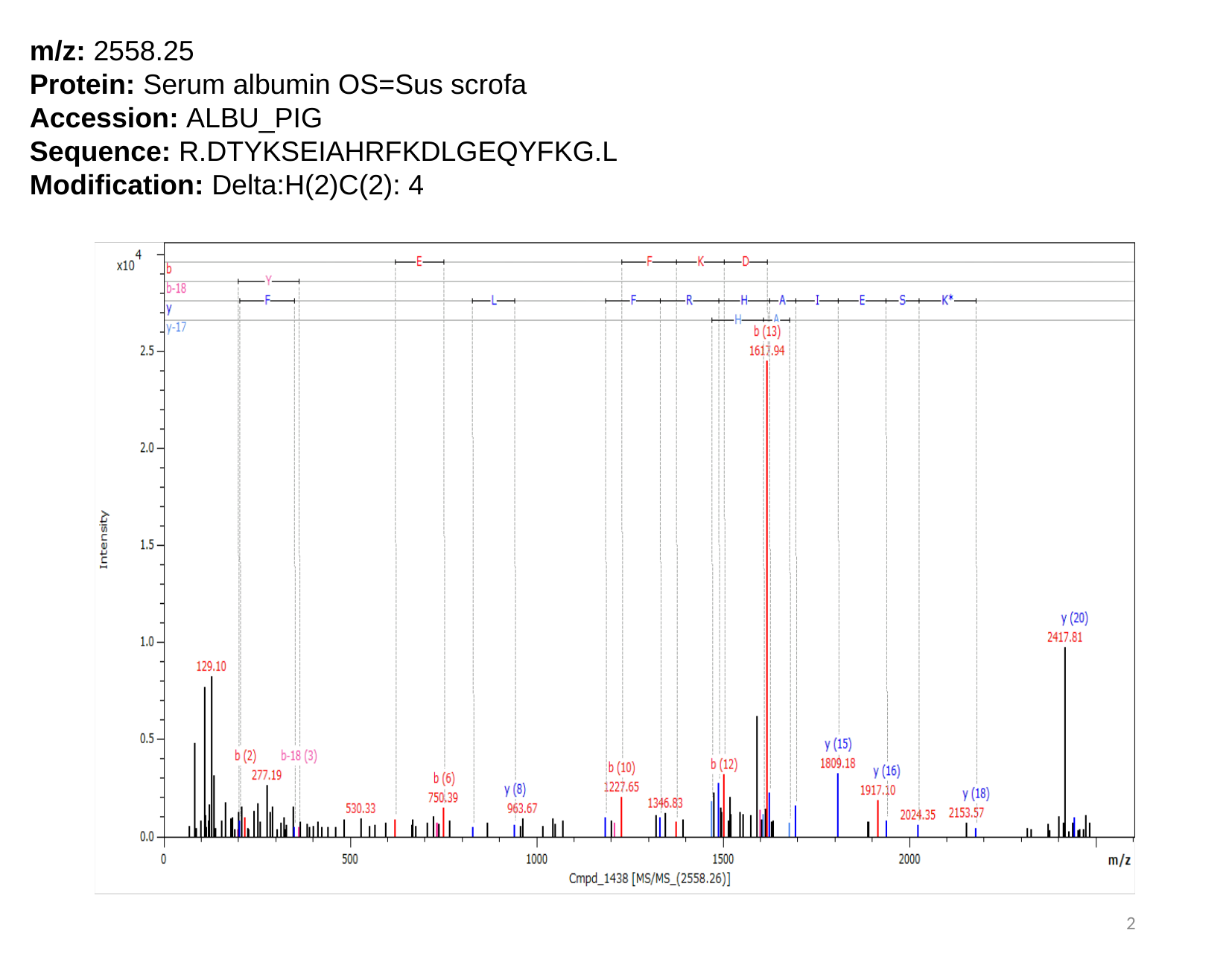

m/z: 2558.25
Protein: Serum albumin OS=Sus scrofa
Accession: ALBU_PIG
Sequence: R.DTYKSEIAHRFKDLGEQYFKG.L
Modification: Delta:H(2)C(2): 4
2

## Slide 3
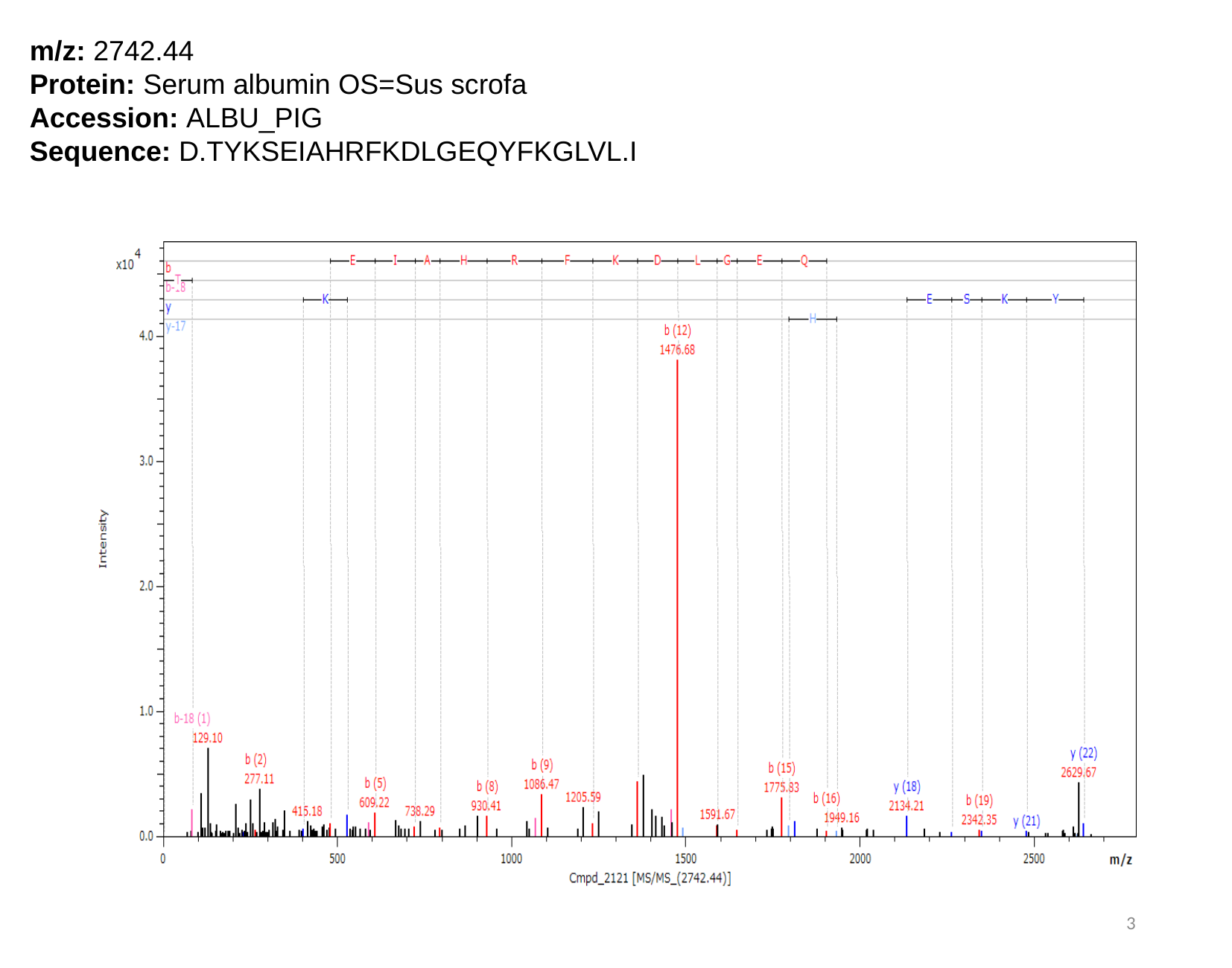

m/z: 2742.44
Protein: Serum albumin OS=Sus scrofa
Accession: ALBU_PIG
Sequence: D.TYKSEIAHRFKDLGEQYFKGLVL.I
3

## Slide 4
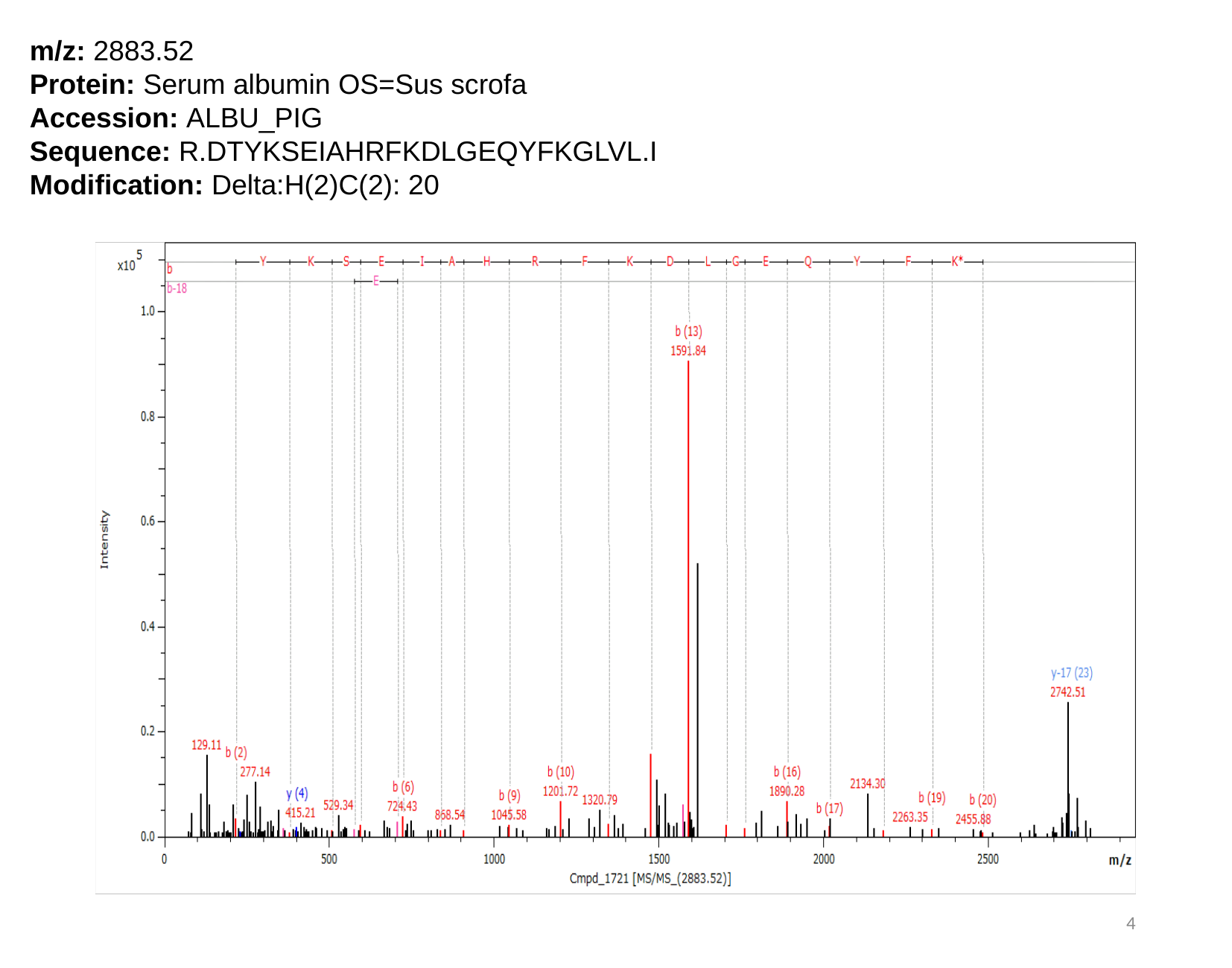

m/z: 2883.52
Protein: Serum albumin OS=Sus scrofa
Accession: ALBU_PIG
Sequence: R.DTYKSEIAHRFKDLGEQYFKGLVL.I
Modification: Delta:H(2)C(2): 20
4

## Slide 5
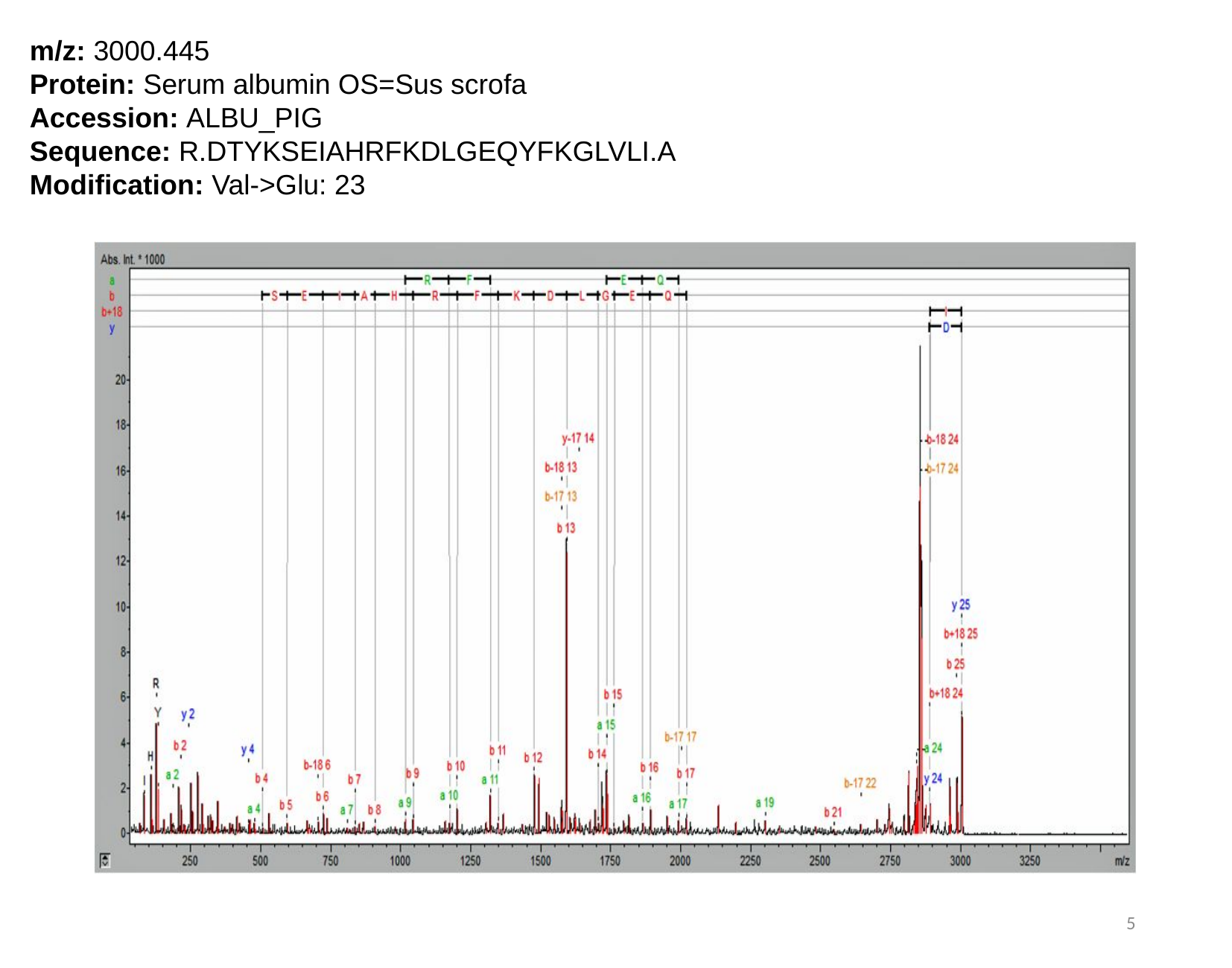

m/z: 3000.445
Protein: Serum albumin OS=Sus scrofa
Accession: ALBU_PIG
Sequence: R.DTYKSEIAHRFKDLGEQYFKGLVLI.A
Modification: Val->Glu: 23
5

## Slide 6
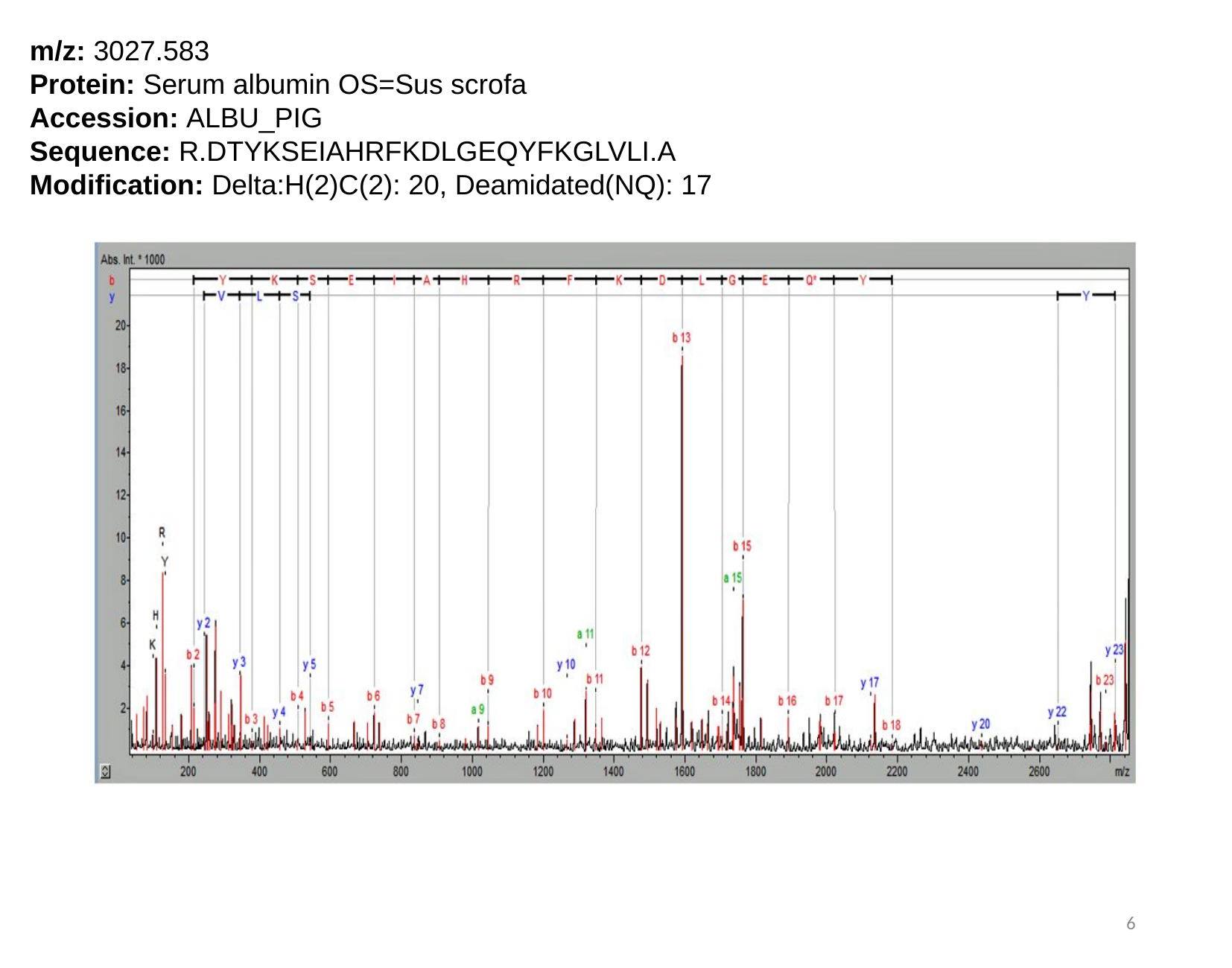

m/z: 3027.583
Protein: Serum albumin OS=Sus scrofa
Accession: ALBU_PIG
Sequence: R.DTYKSEIAHRFKDLGEQYFKGLVLI.A
Modification: Delta:H(2)C(2): 20, Deamidated(NQ): 17
6

## Slide 7
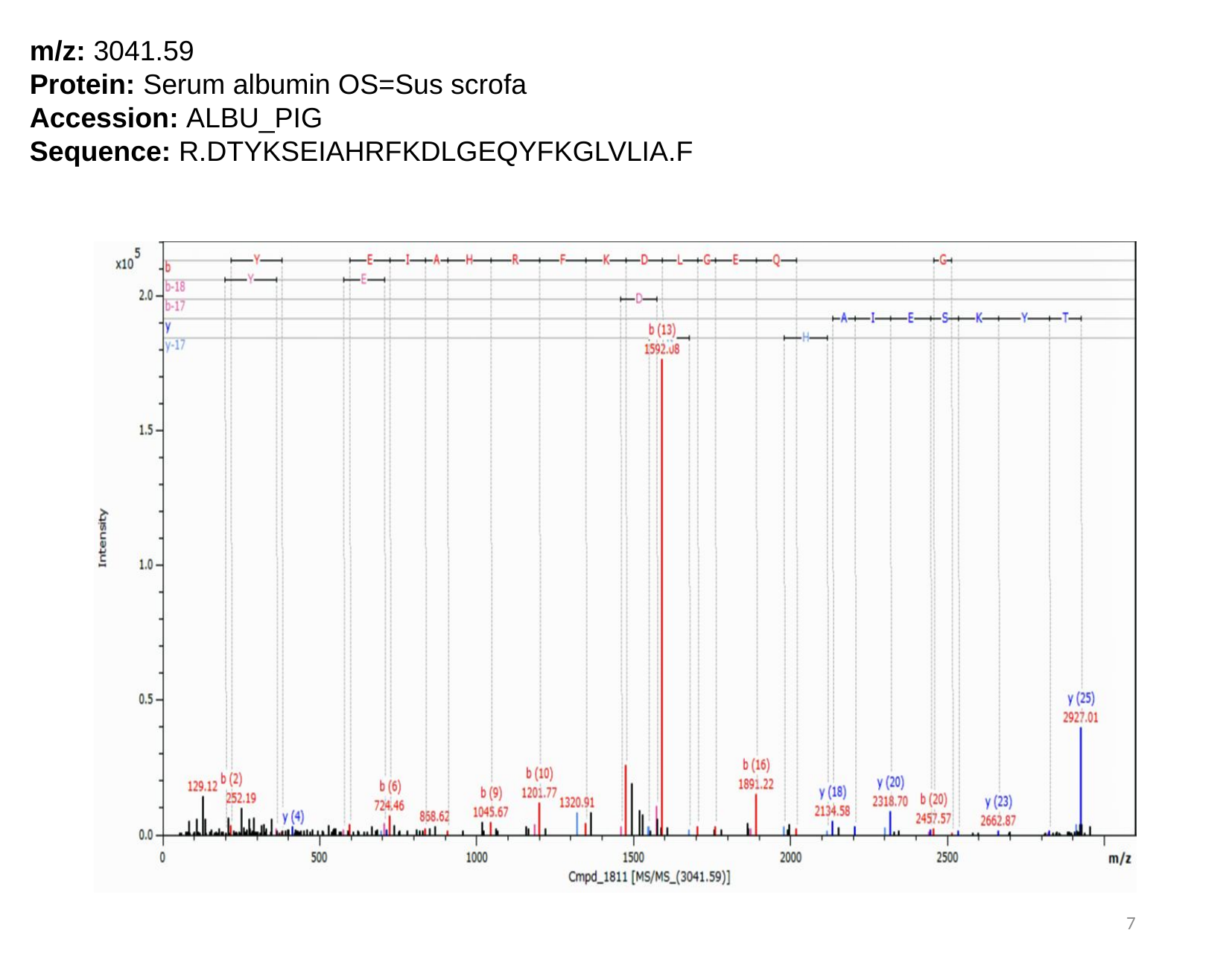

m/z: 3041.59
Protein: Serum albumin OS=Sus scrofa
Accession: ALBU_PIG
Sequence: R.DTYKSEIAHRFKDLGEQYFKGLVLIA.F
7

## Slide 8
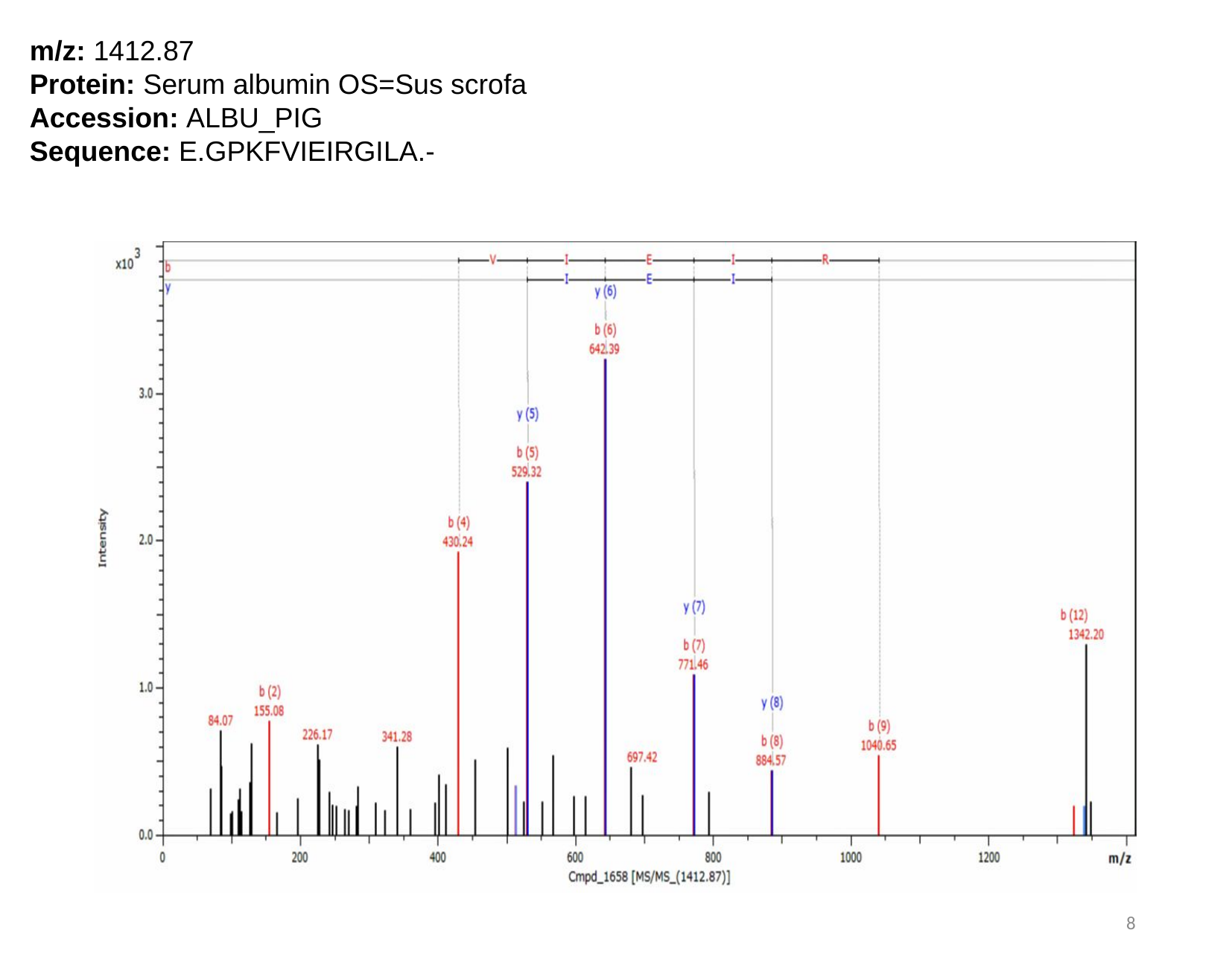

m/z: 1412.87
Protein: Serum albumin OS=Sus scrofa
Accession: ALBU_PIG
Sequence: E.GPKFVIEIRGILA.-
8

## Slide 9
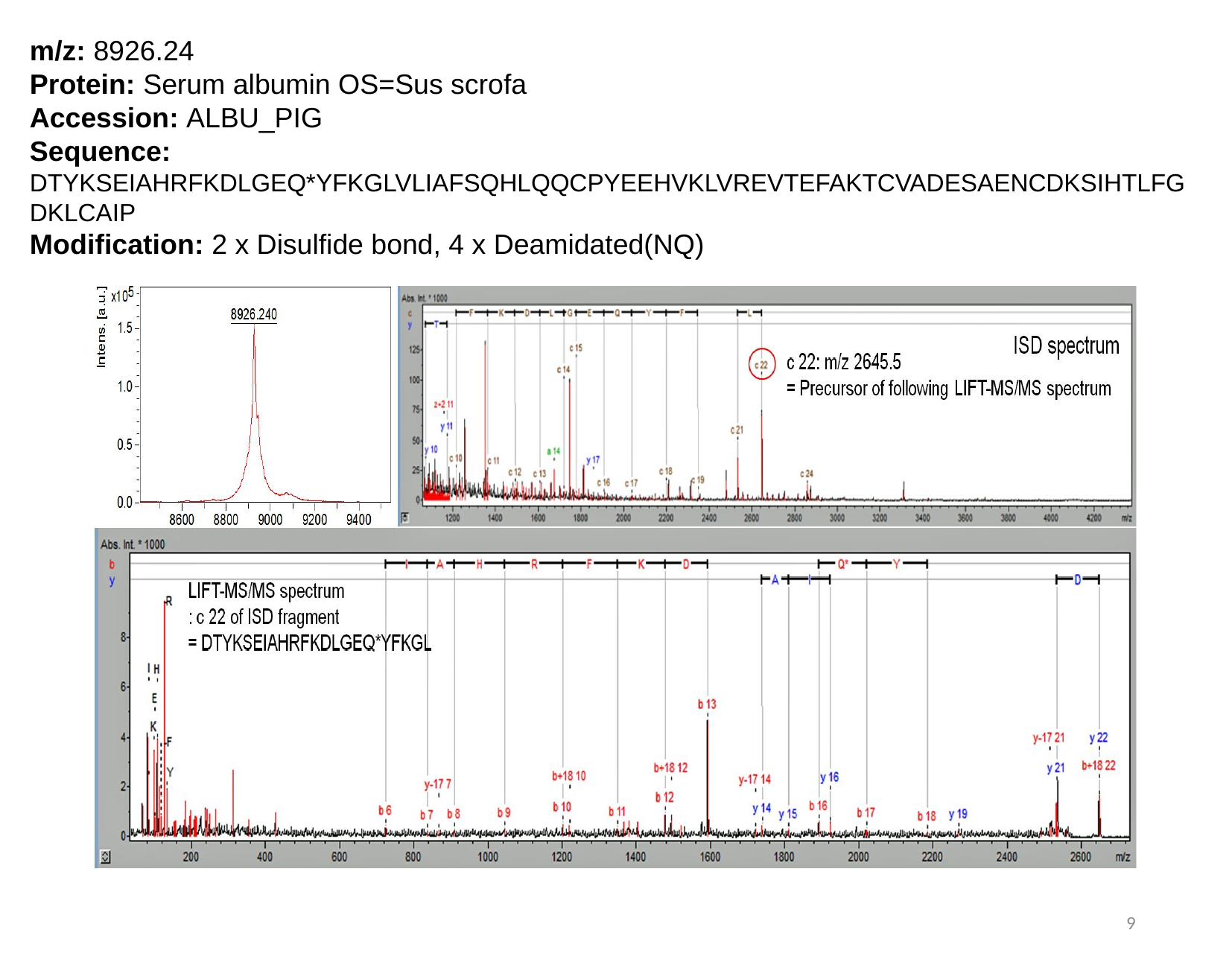

m/z: 8926.24
Protein: Serum albumin OS=Sus scrofa
Accession: ALBU_PIG
Sequence: DTYKSEIAHRFKDLGEQ*YFKGLVLIAFSQHLQQCPYEEHVKLVREVTEFAKTCVADESAENCDKSIHTLFGDKLCAIP
Modification: 2 x Disulfide bond, 4 x Deamidated(NQ)
9

## Slide 10
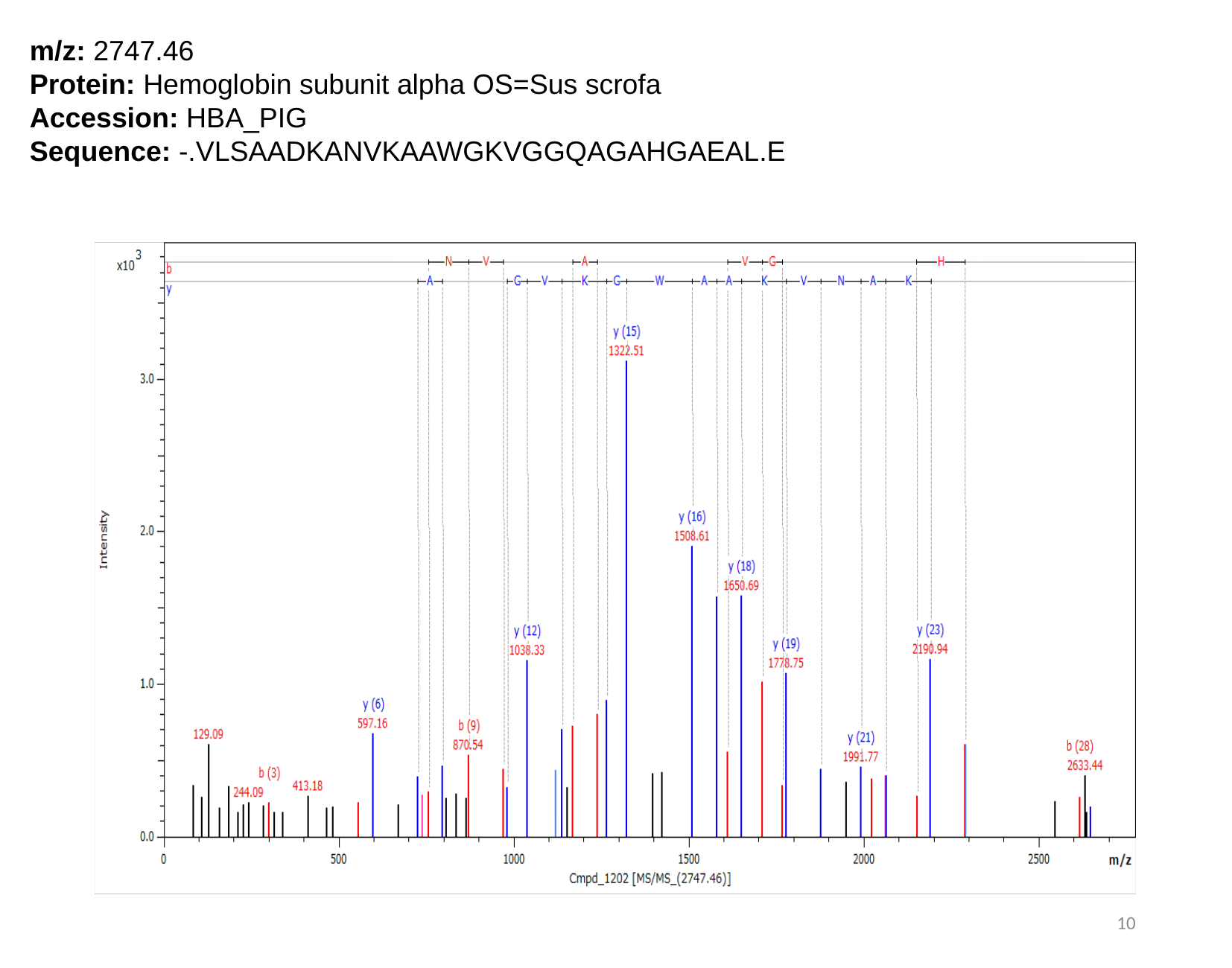

m/z: 2747.46
Protein: Hemoglobin subunit alpha OS=Sus scrofa
Accession: HBA_PIG
Sequence: -.VLSAADKANVKAAWGKVGGQAGAHGAEAL.E
10

## Slide 11
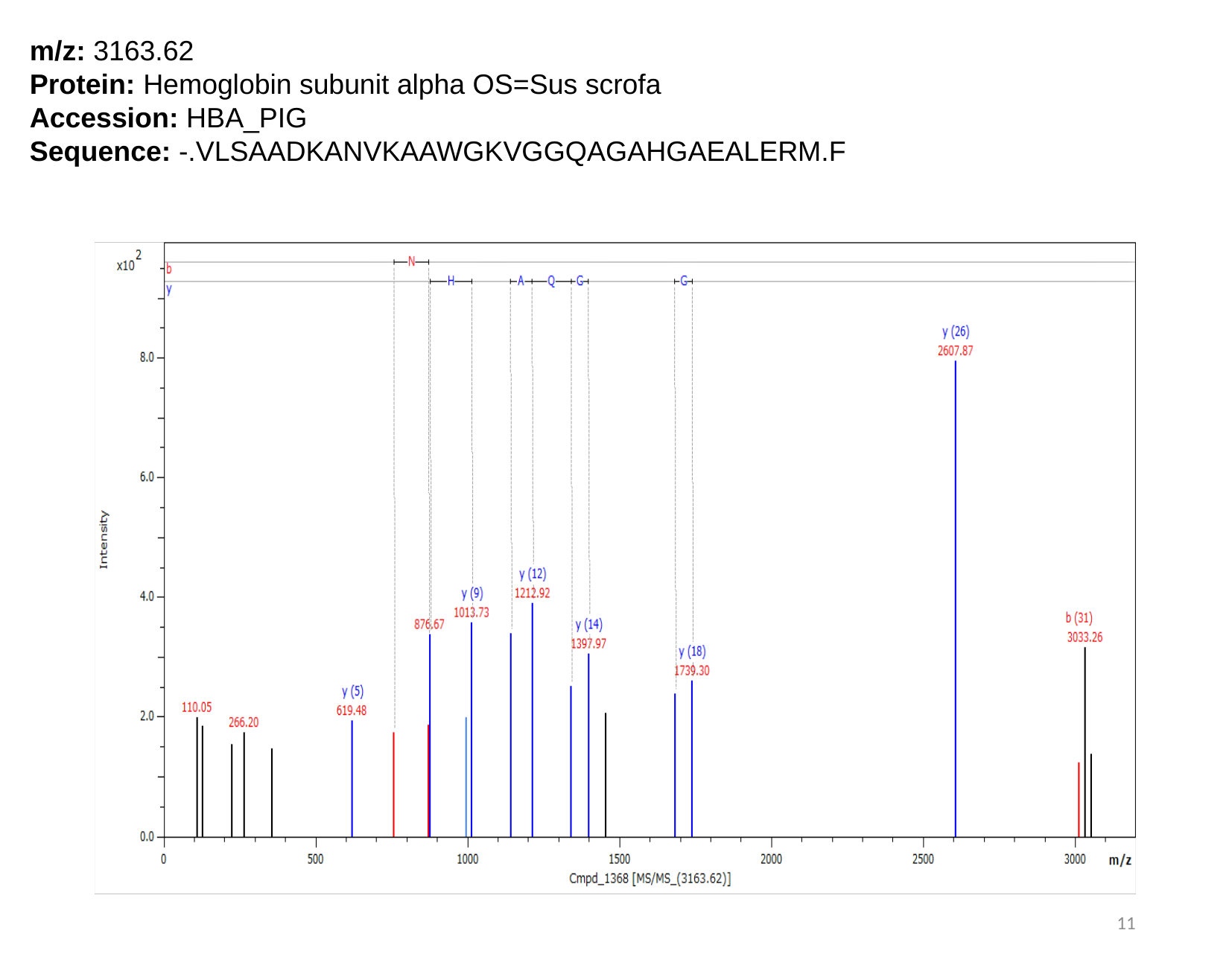

m/z: 3163.62
Protein: Hemoglobin subunit alpha OS=Sus scrofa
Accession: HBA_PIG
Sequence: -.VLSAADKANVKAAWGKVGGQAGAHGAEALERM.F
11

## Slide 12
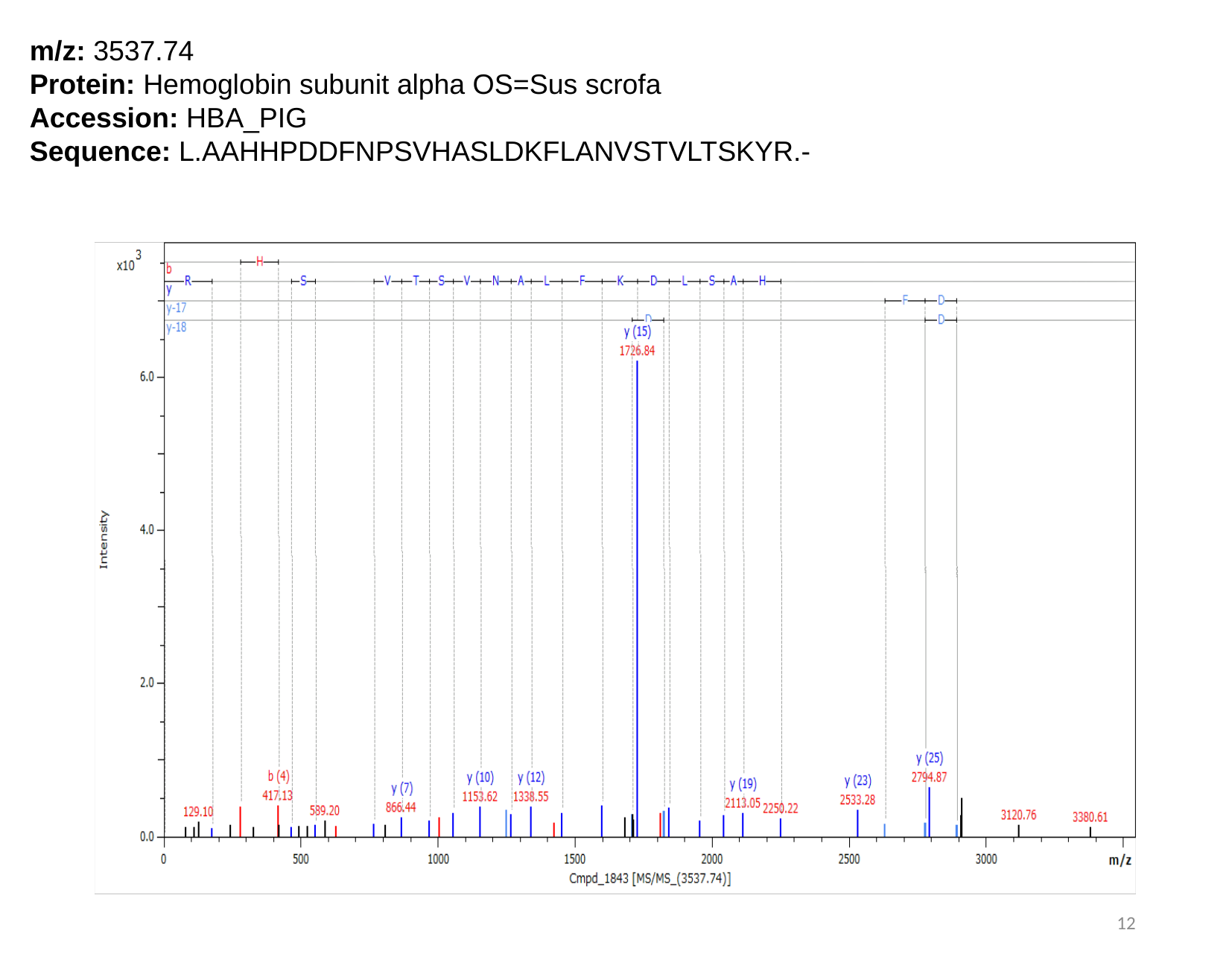

m/z: 3537.74
Protein: Hemoglobin subunit alpha OS=Sus scrofa
Accession: HBA_PIG
Sequence: L.AAHHPDDFNPSVHASLDKFLANVSTVLTSKYR.-
12

## Slide 13
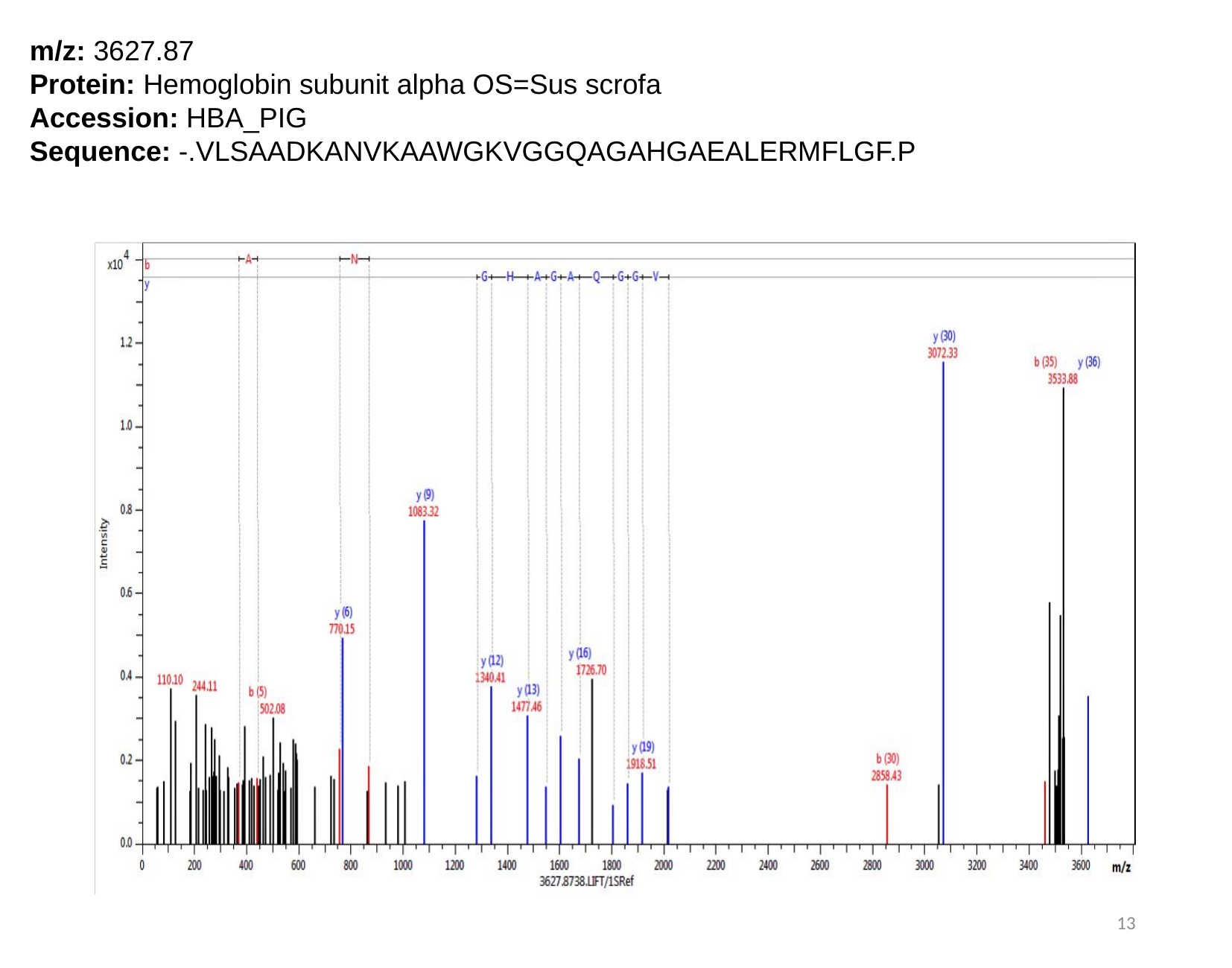

m/z: 3627.87
Protein: Hemoglobin subunit alpha OS=Sus scrofa
Accession: HBA_PIG
Sequence: -.VLSAADKANVKAAWGKVGGQAGAHGAEALERMFLGF.P
13

## Slide 14
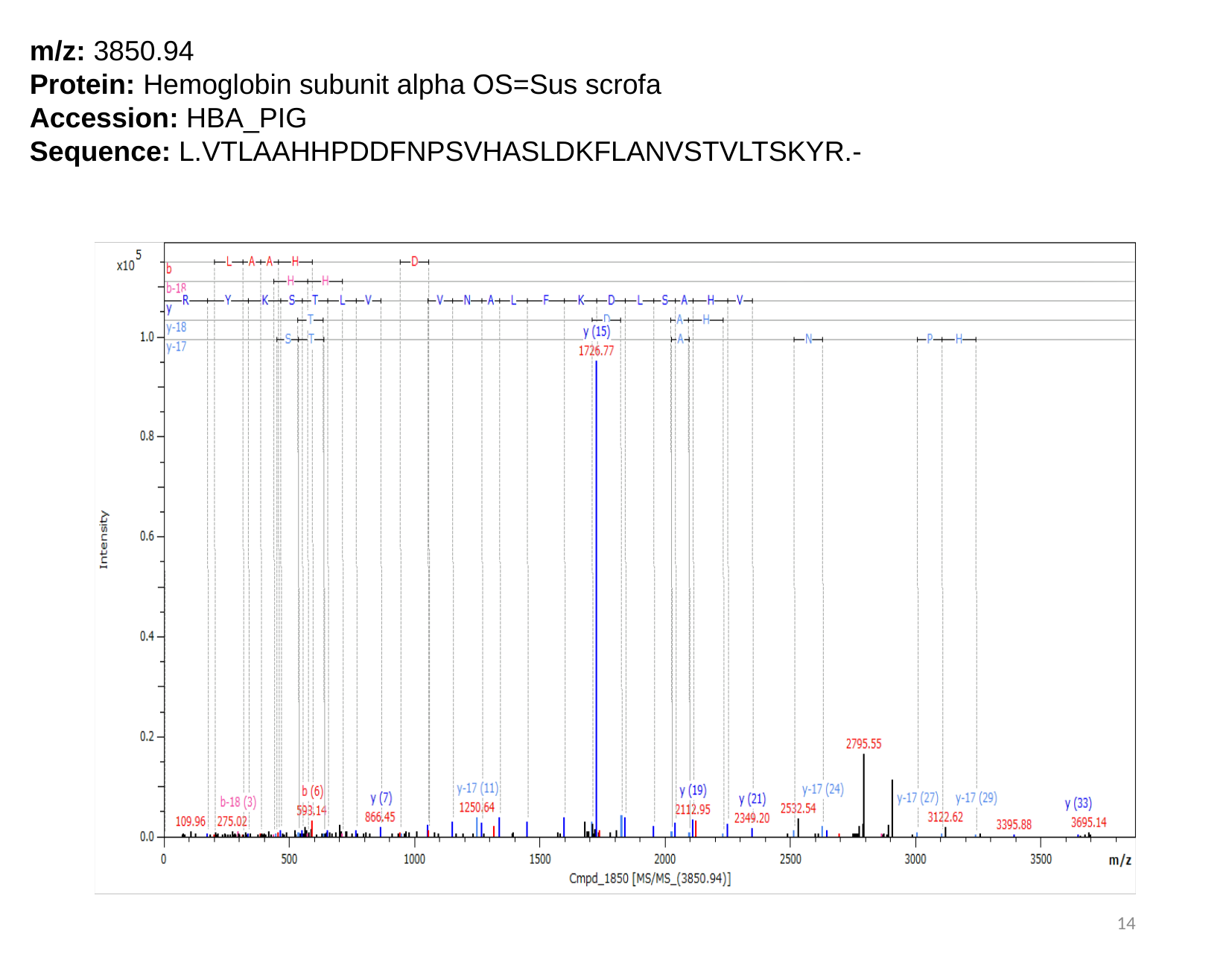

m/z: 3850.94
Protein: Hemoglobin subunit alpha OS=Sus scrofa
Accession: HBA_PIG
Sequence: L.VTLAAHHPDDFNPSVHASLDKFLANVSTVLTSKYR.-
14

## Slide 15
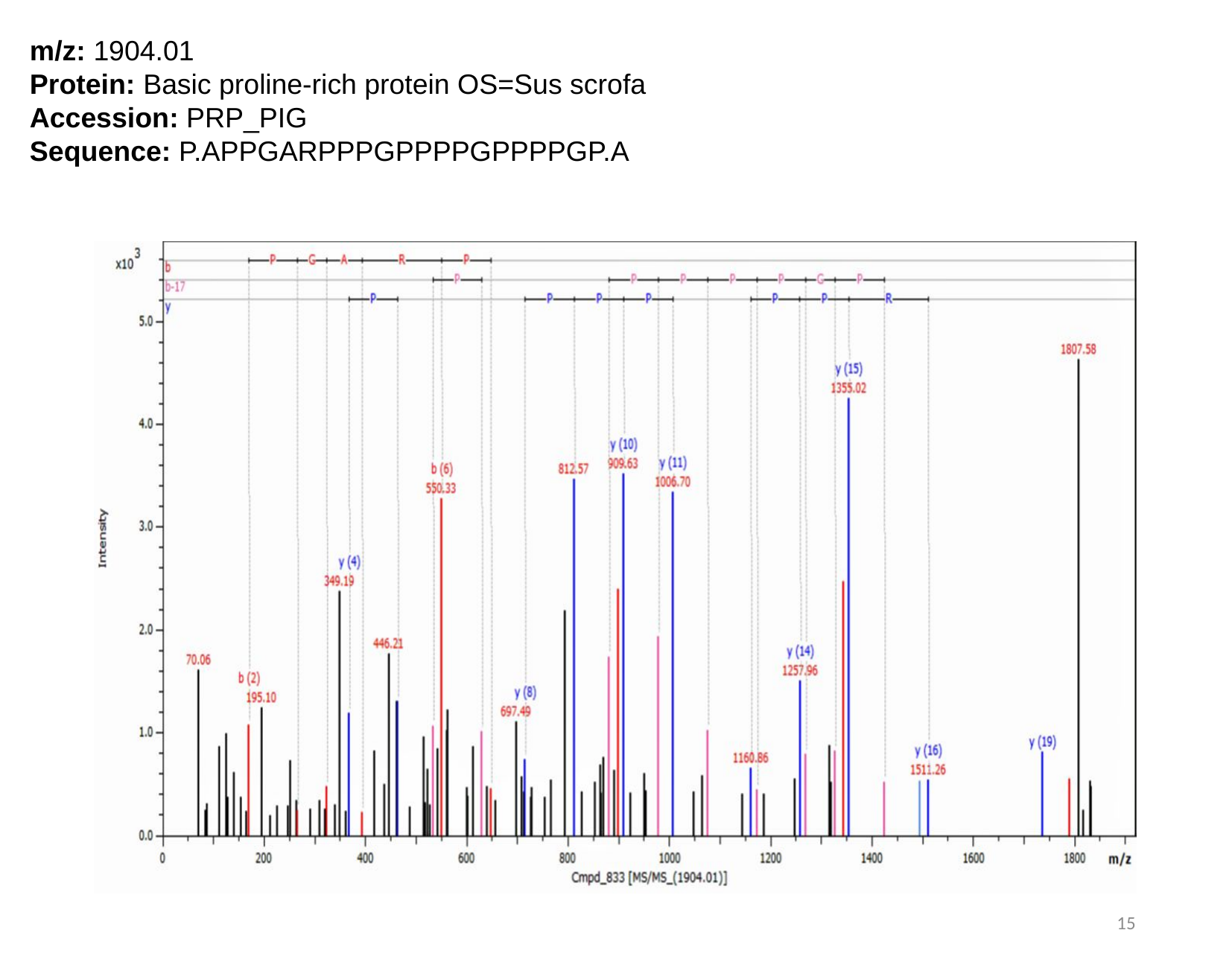

m/z: 1904.01
Protein: Basic proline-rich protein OS=Sus scrofa　Accession: PRP_PIG
Sequence: P.APPGARPPPGPPPPGPPPPGP.A
15

## Slide 16
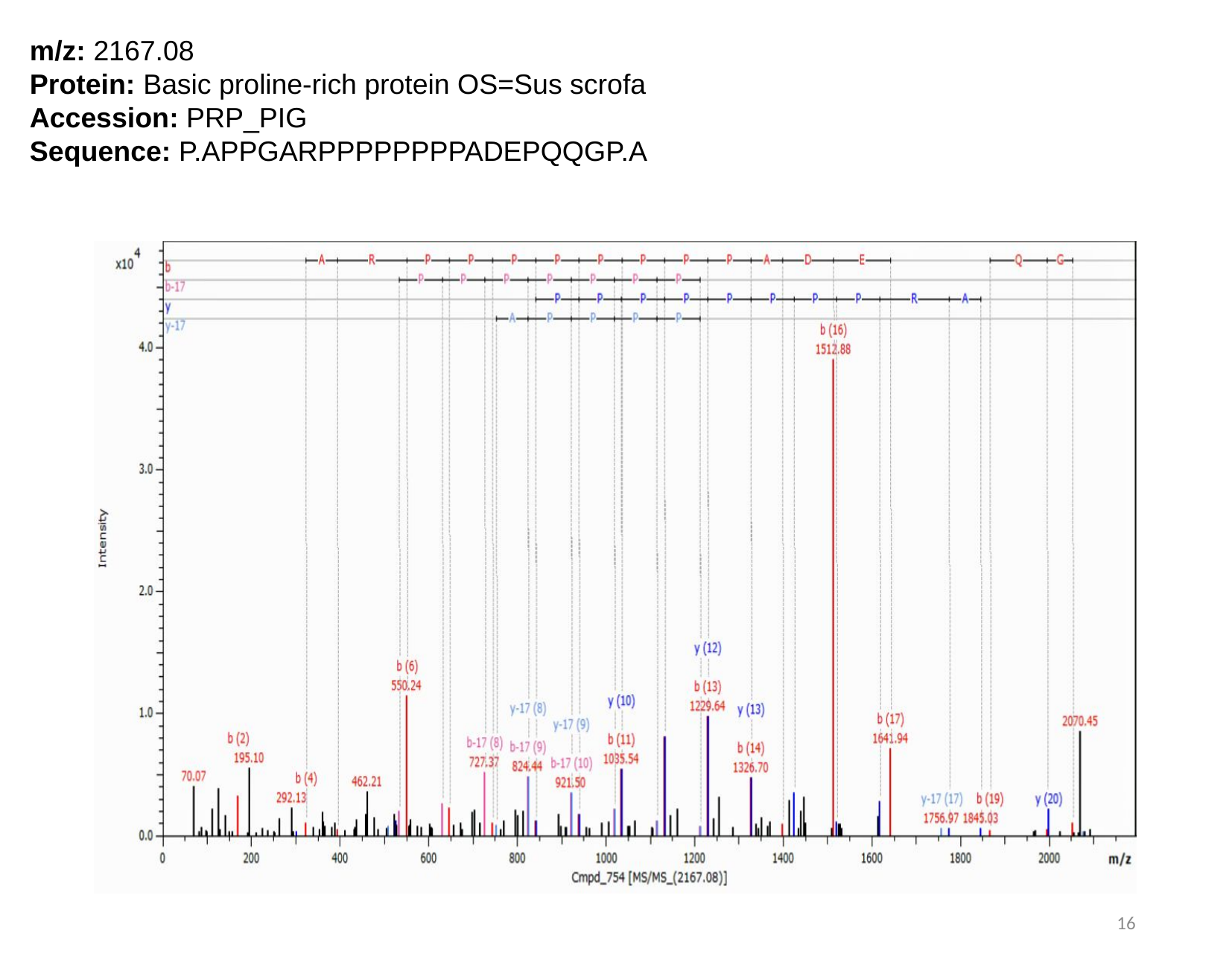

m/z: 2167.08
Protein: Basic proline-rich protein OS=Sus scrofa　Accession: PRP_PIG
Sequence: P.APPGARPPPPPPPPADEPQQGP.A
16

## Slide 17
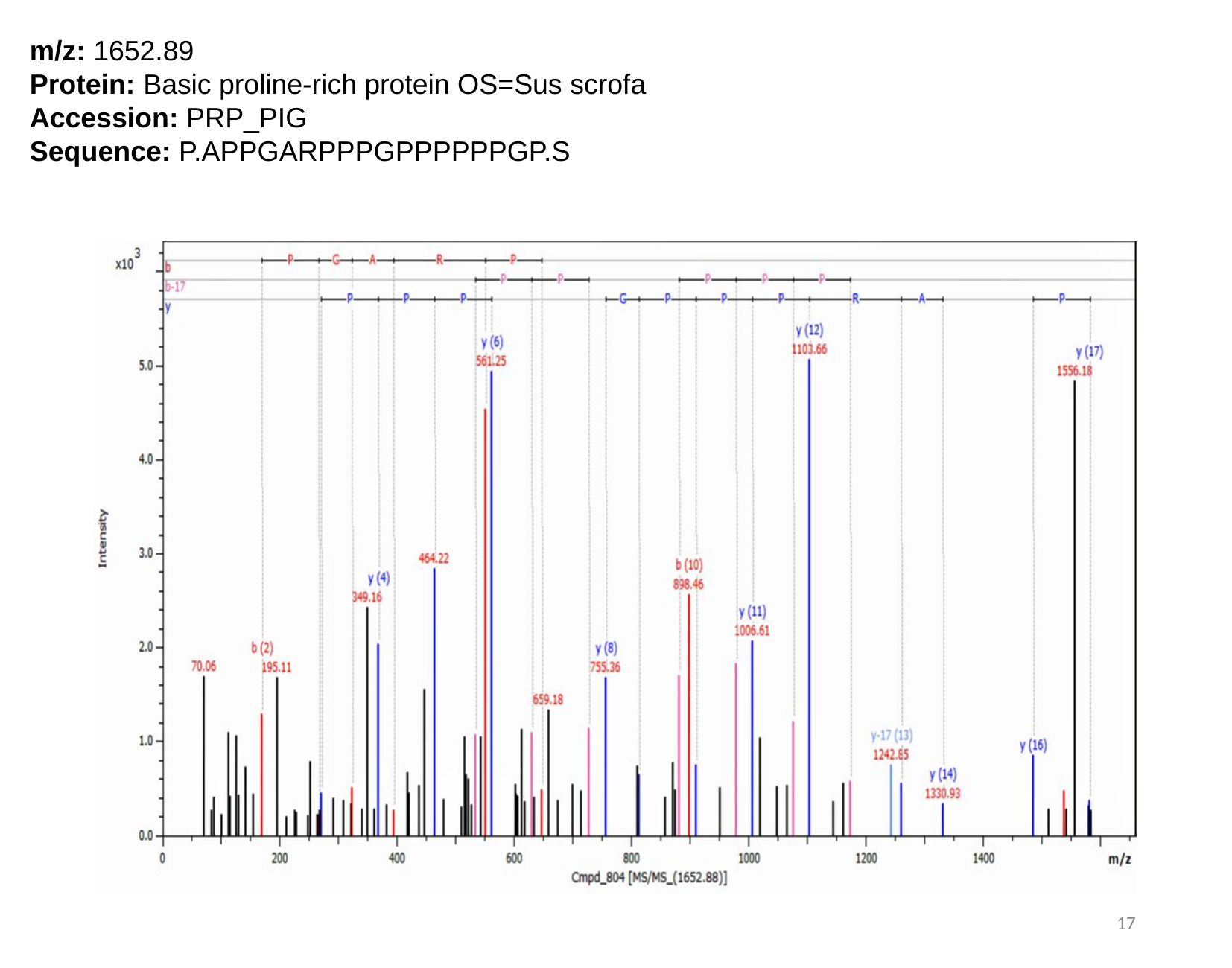

m/z: 1652.89
Protein: Basic proline-rich protein OS=Sus scrofa　Accession: PRP_PIG
Sequence: P.APPGARPPPGPPPPPPGP.S
17

## Slide 18
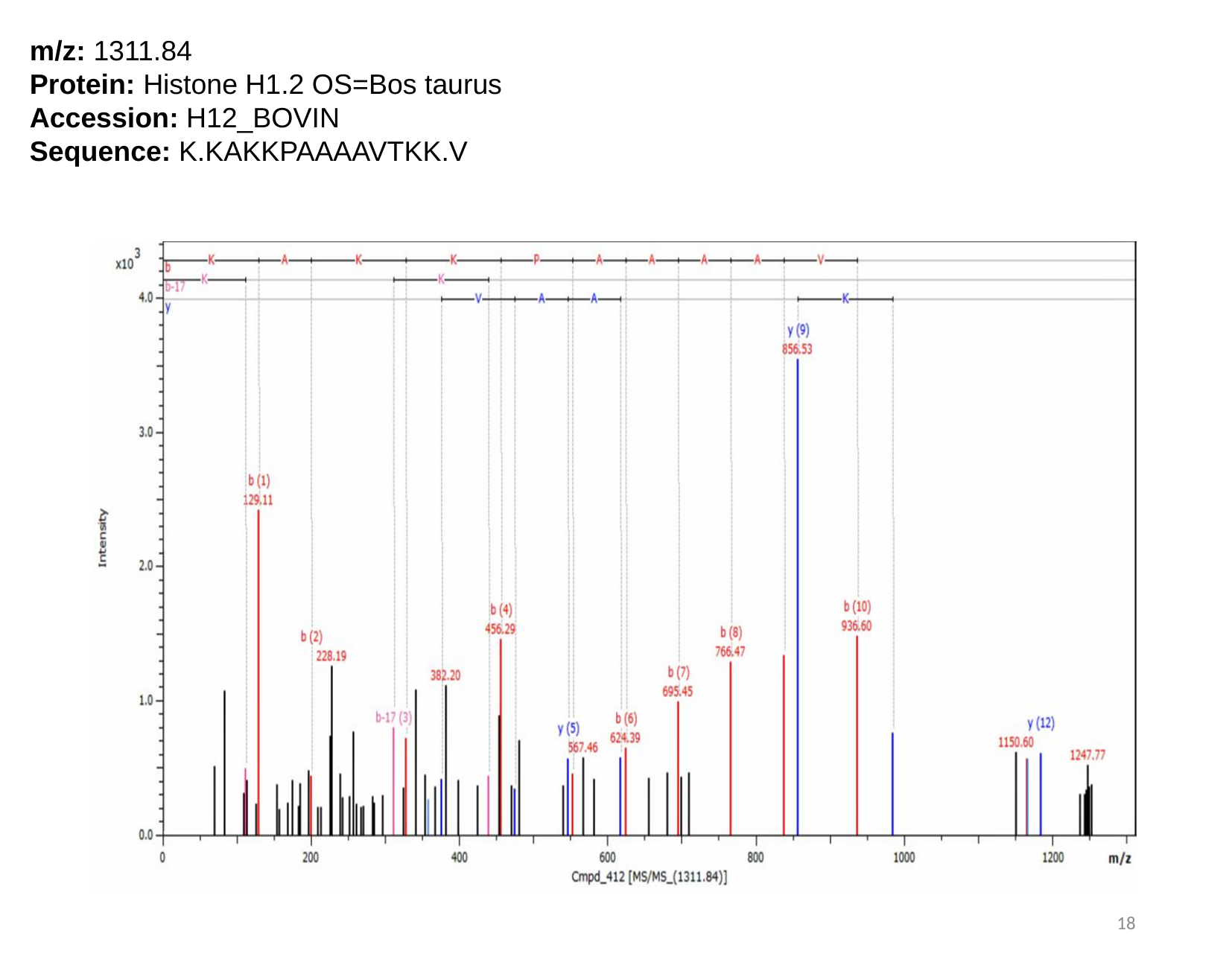

m/z: 1311.84
Protein: Histone H1.2 OS=Bos taurus
Accession: H12_BOVIN
Sequence: K.KAKKPAAAAVTKK.V
18

## Slide 19
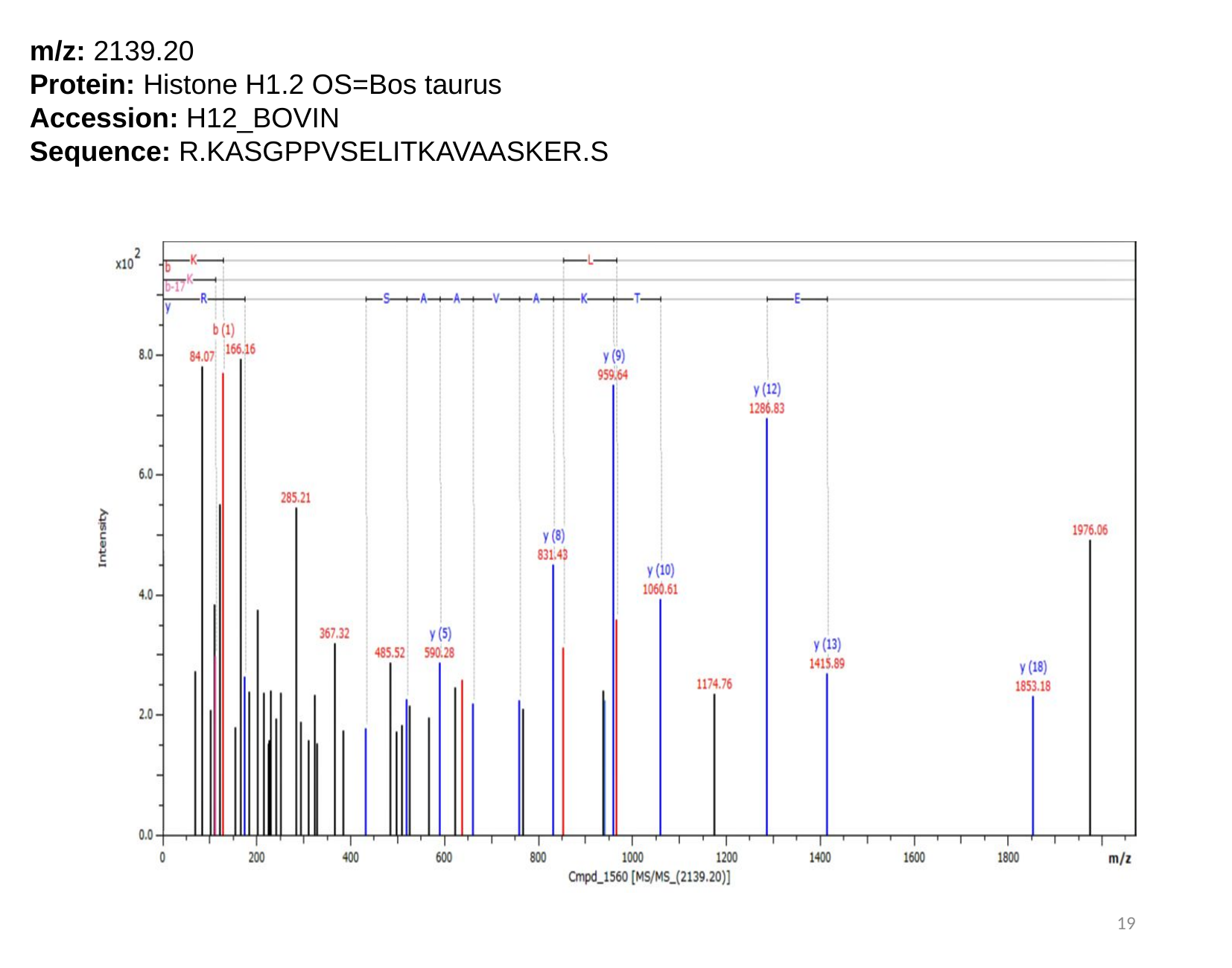

m/z: 2139.20
Protein: Histone H1.2 OS=Bos taurus
Accession: H12_BOVIN
Sequence: R.KASGPPVSELITKAVAASKER.S
19

## Slide 20
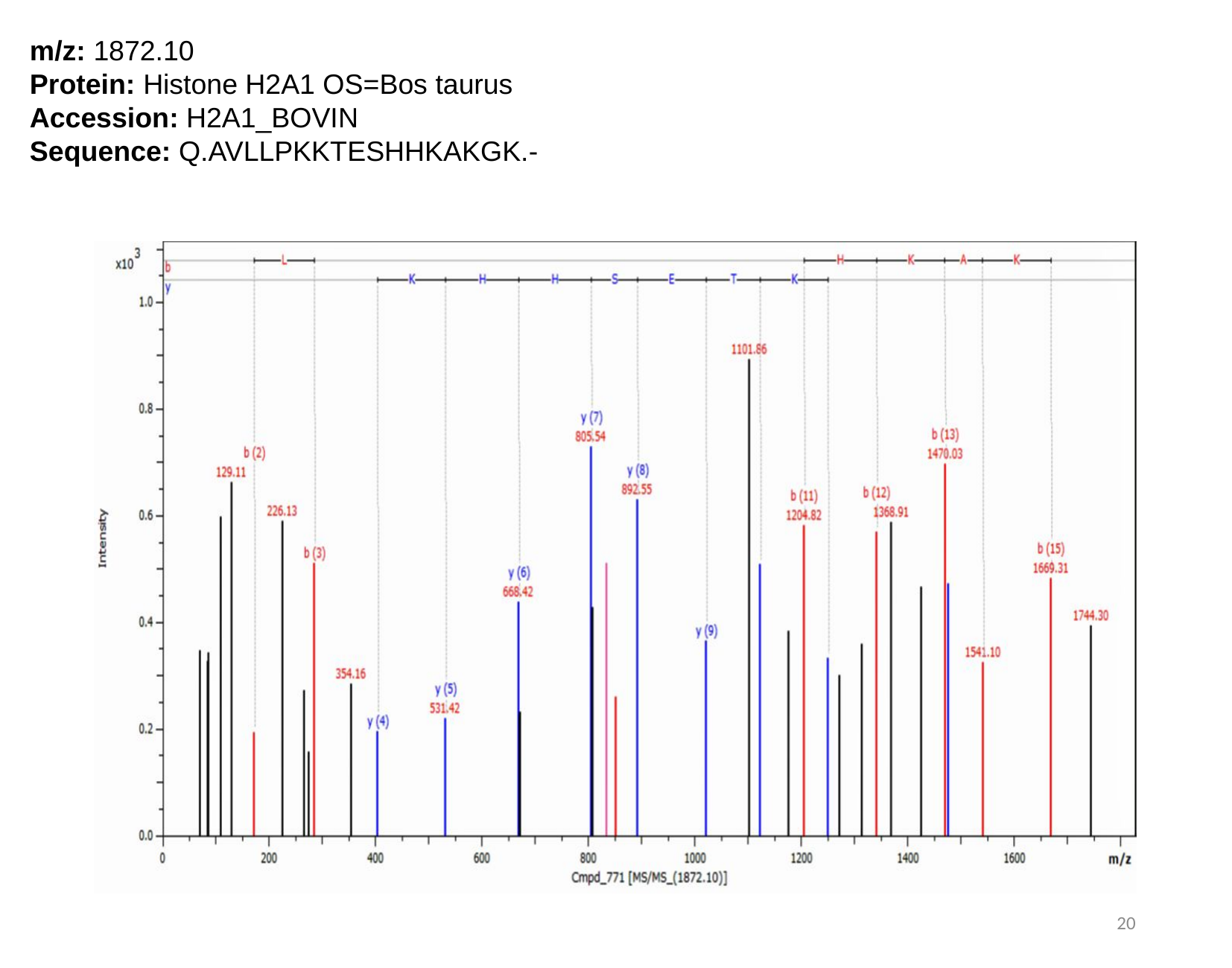

m/z: 1872.10
Protein: Histone H2A1 OS=Bos taurus
Accession: H2A1_BOVIN
Sequence: Q.AVLLPKKTESHHKAKGK.-
20

## Slide 21
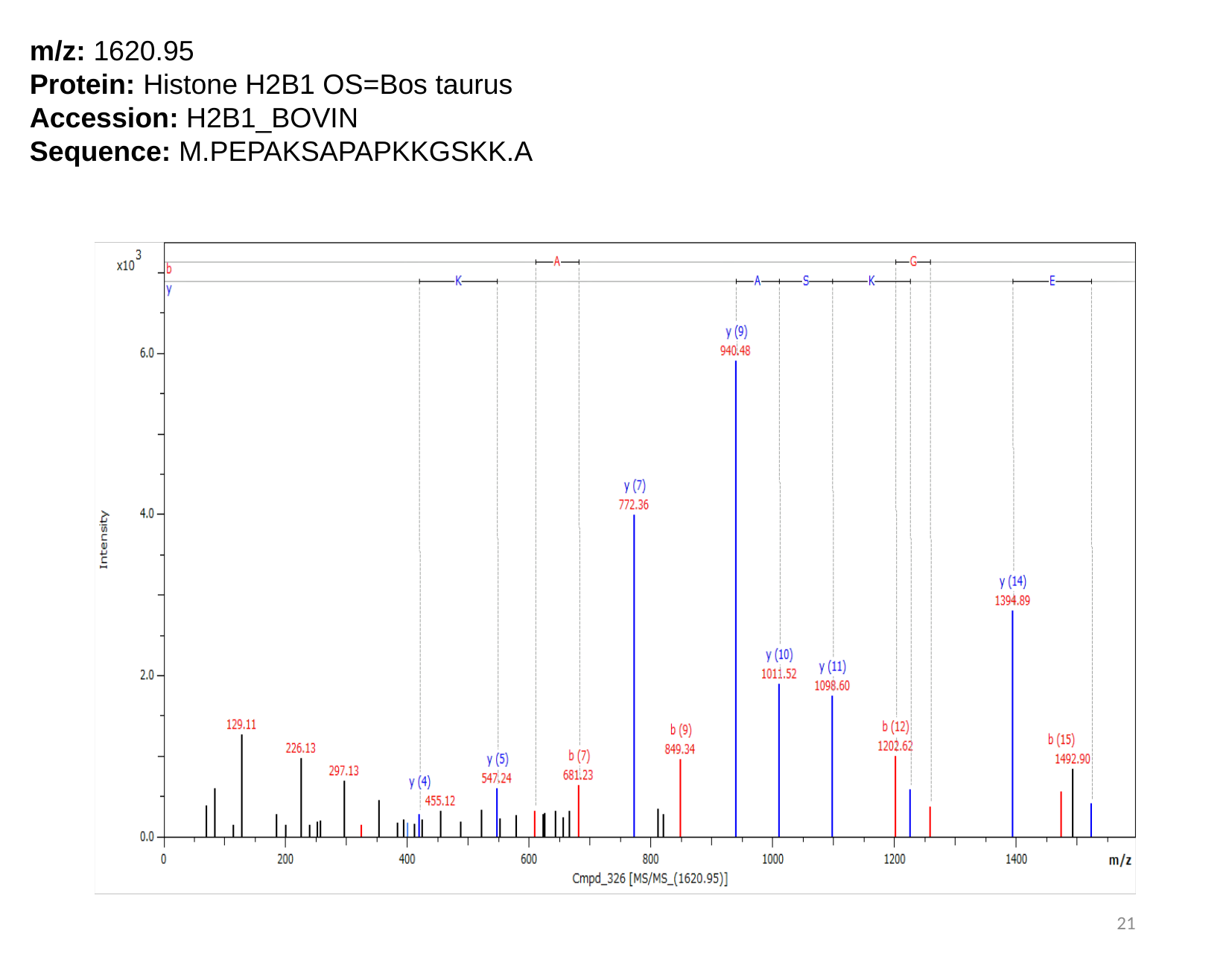

m/z: 1620.95
Protein: Histone H2B1 OS=Bos taurus
Accession: H2B1_BOVIN
Sequence: M.PEPAKSAPAPKKGSKK.A
21

## Slide 22
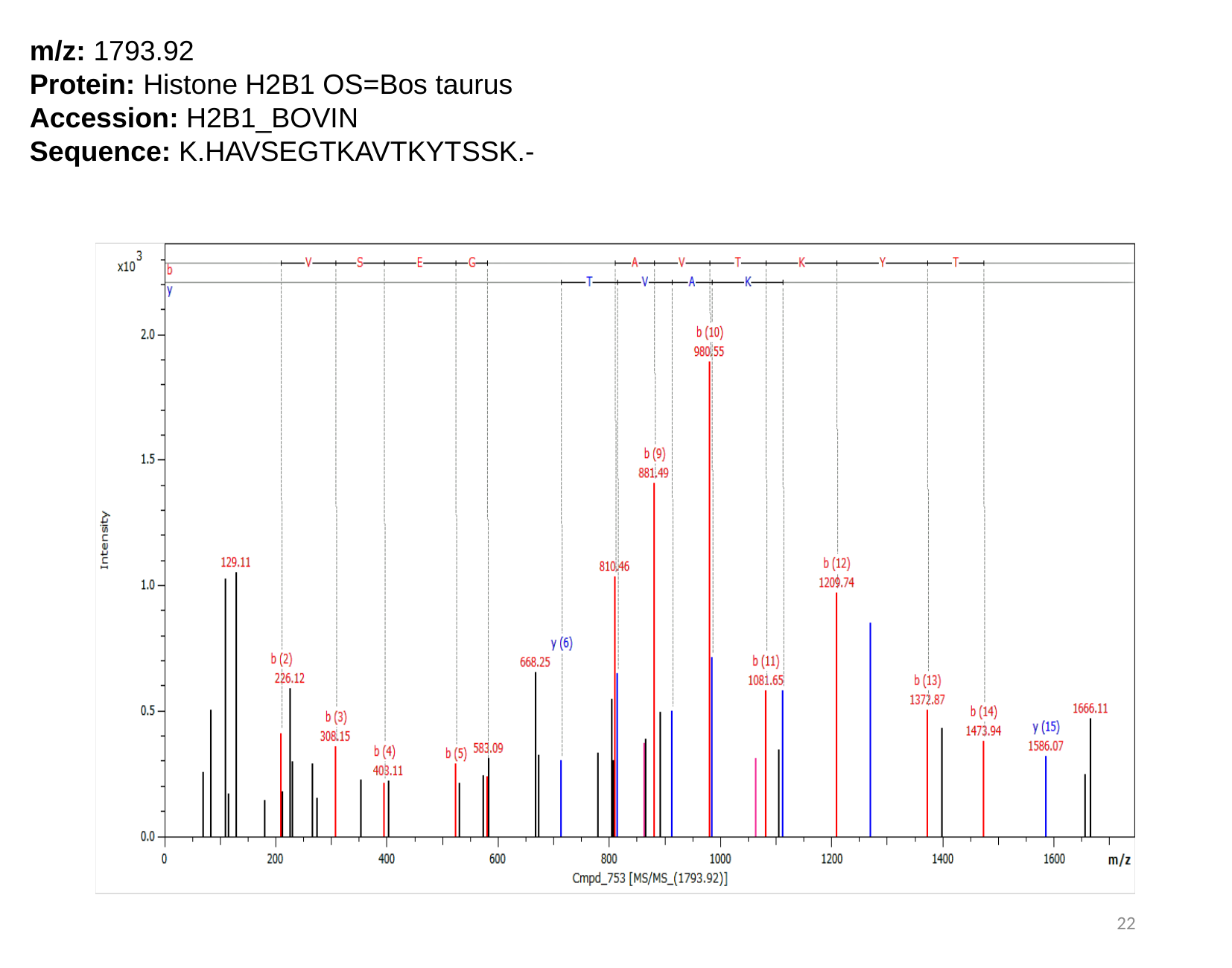

m/z: 1793.92
Protein: Histone H2B1 OS=Bos taurus
Accession: H2B1_BOVIN
Sequence: K.HAVSEGTKAVTKYTSSK.-
22

## Slide 23
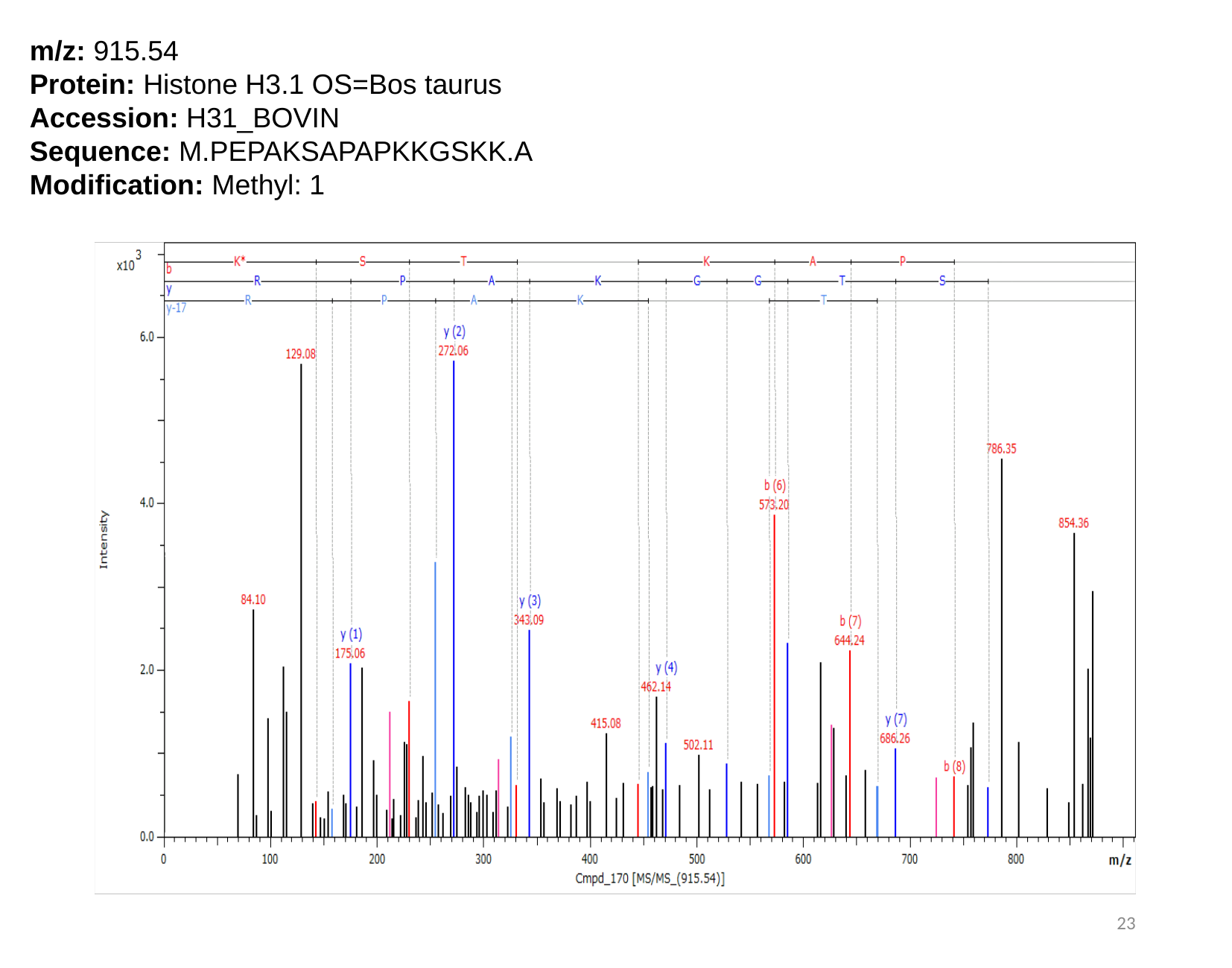

m/z: 915.54
Protein: Histone H3.1 OS=Bos taurus
Accession: H31_BOVIN
Sequence: M.PEPAKSAPAPKKGSKK.A
Modification: Methyl: 1
23

## Slide 24
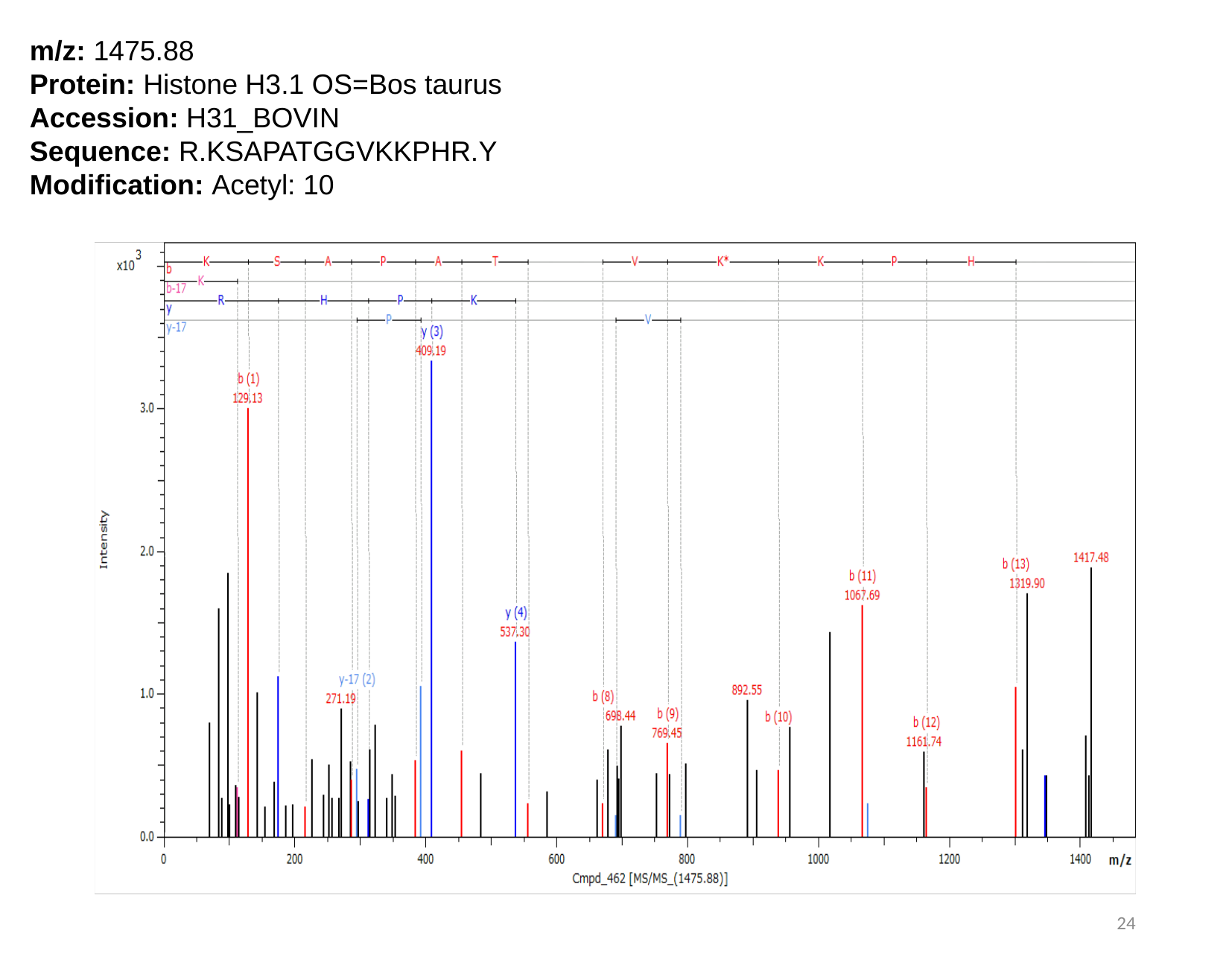

m/z: 1475.88
Protein: Histone H3.1 OS=Bos taurus
Accession: H31_BOVIN
Sequence: R.KSAPATGGVKKPHR.Y
Modification: Acetyl: 10
24

## Slide 25
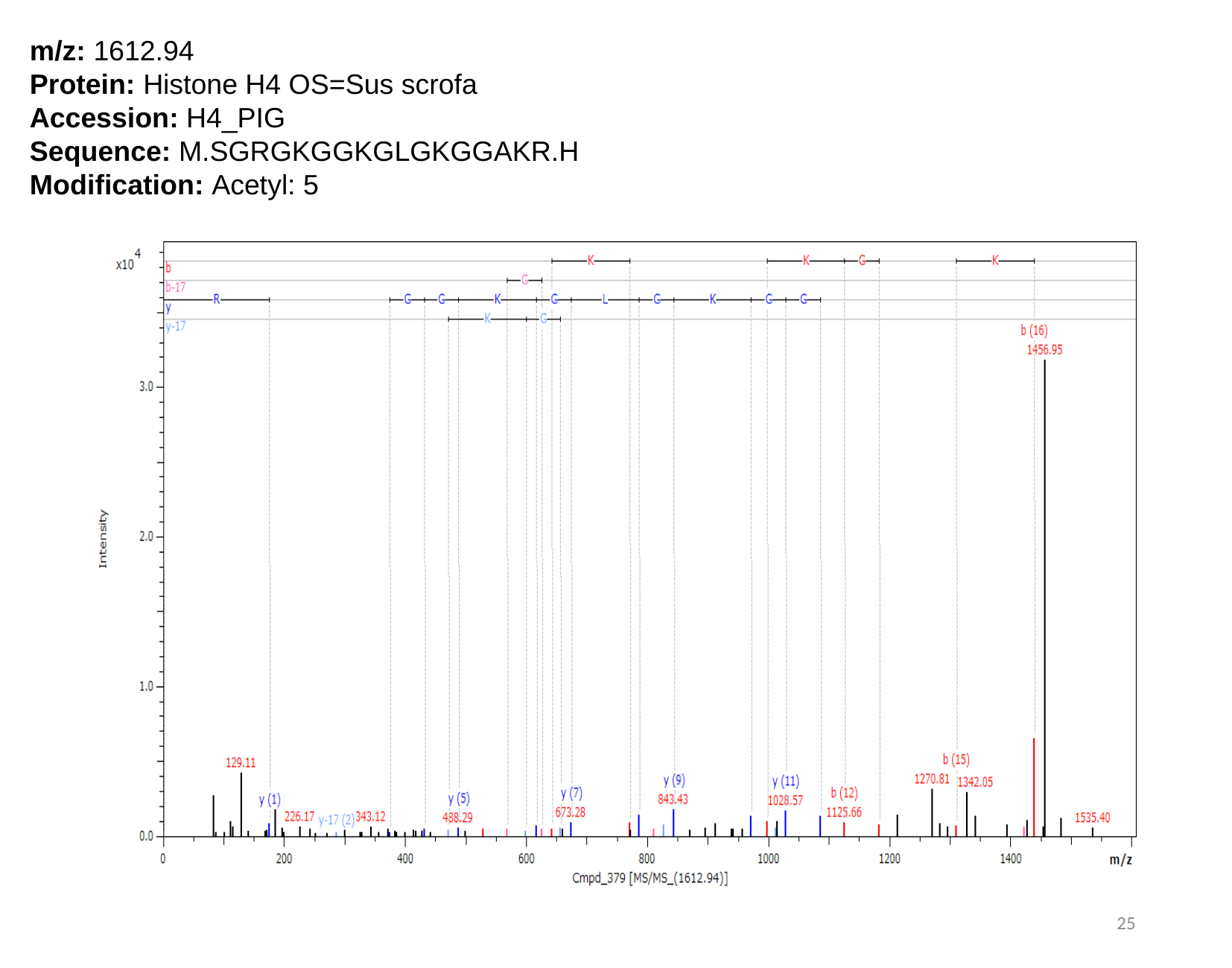

m/z: 1612.94
Protein: Histone H4 OS=Sus scrofa
Accession: H4_PIG
Sequence: M.SGRGKGGKGLGKGGAKR.H
Modification: Acetyl: 5
25

## Slide 26
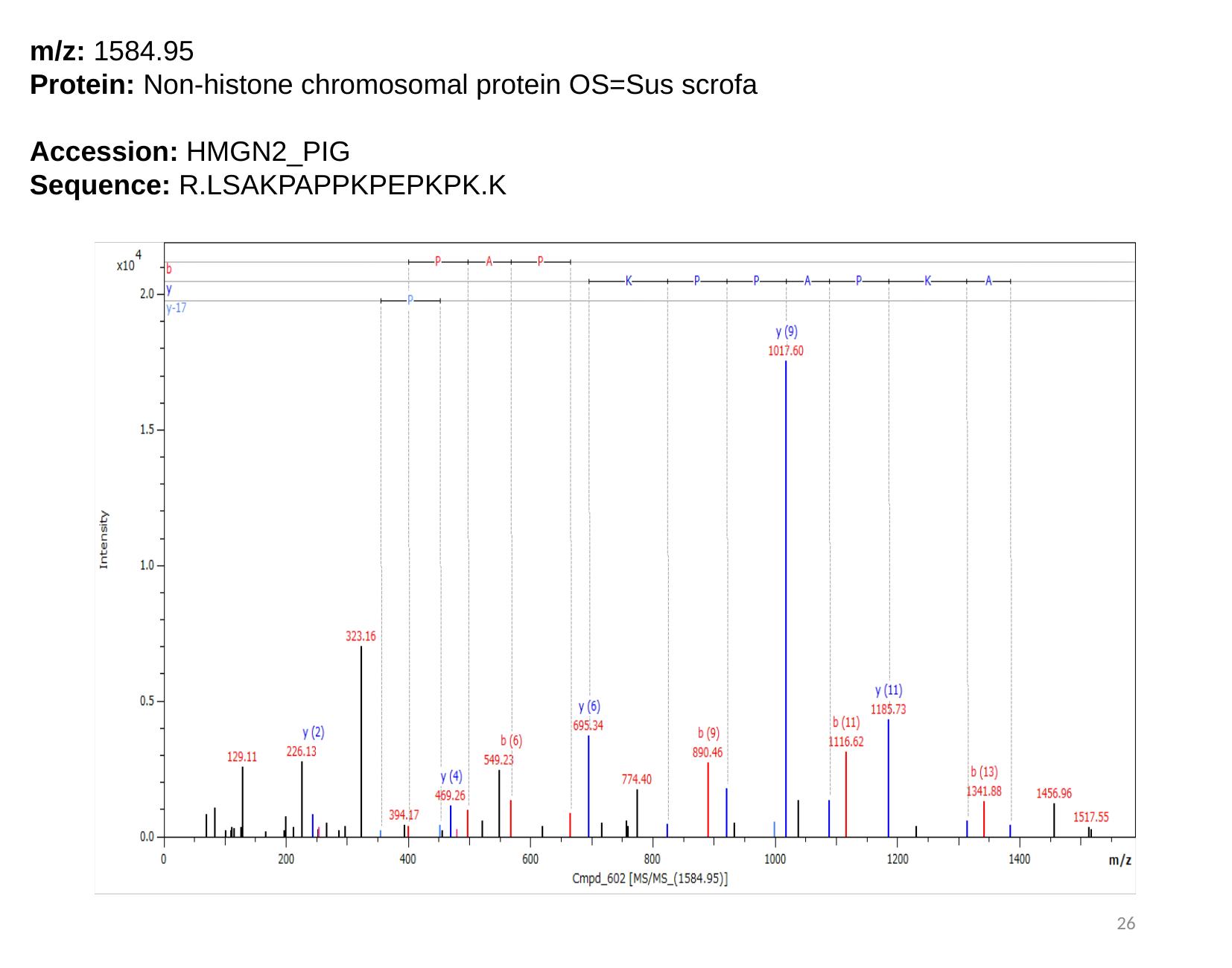

m/z: 1584.95
Protein: Non-histone chromosomal protein OS=Sus scrofa
Accession: HMGN2_PIG
Sequence: R.LSAKPAPPKPEPKPK.K
26

## Slide 27
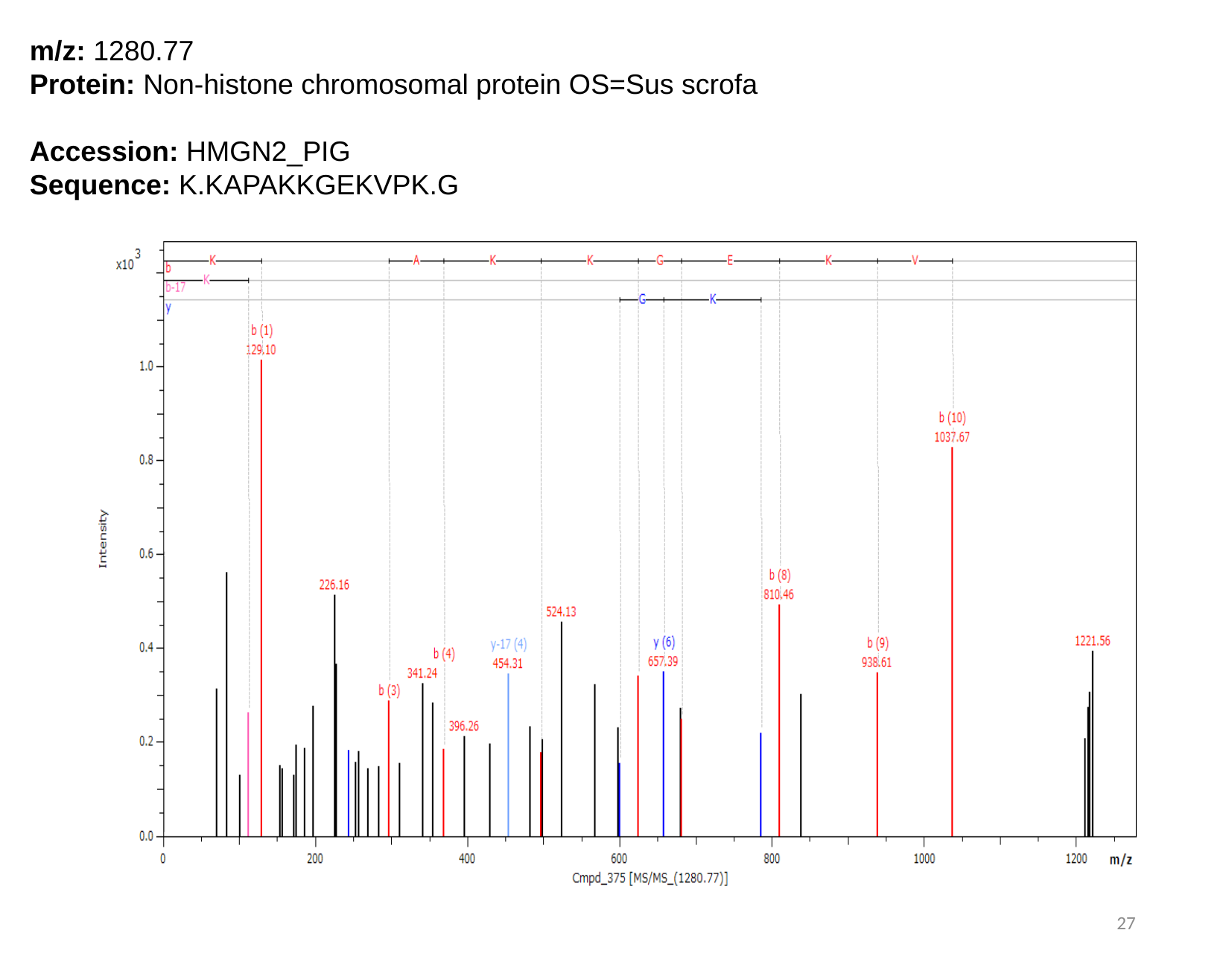

m/z: 1280.77
Protein: Non-histone chromosomal protein OS=Sus scrofa
Accession: HMGN2_PIG
Sequence: K.KAPAKKGEKVPK.G
27

## Slide 28
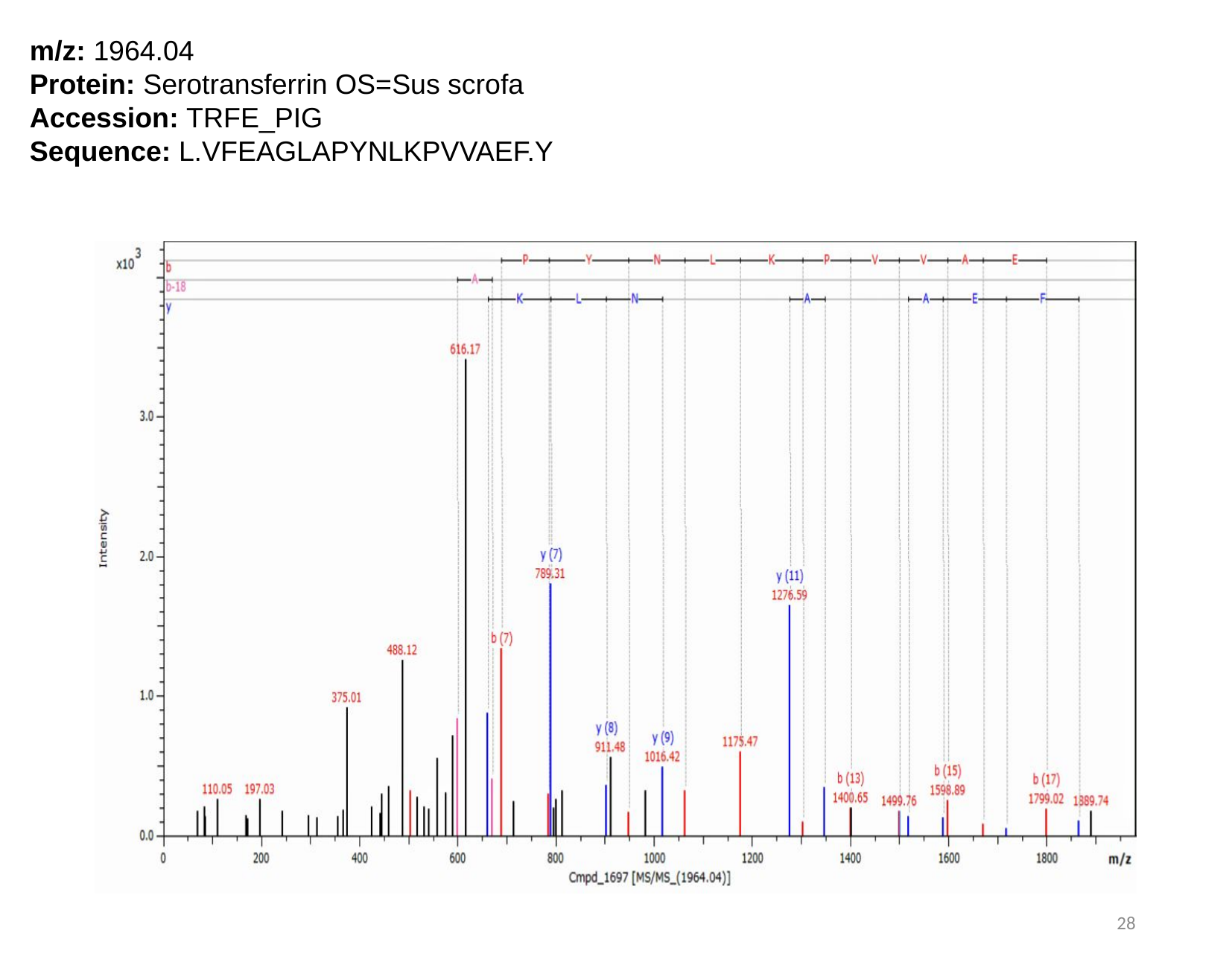

m/z: 1964.04
Protein: Serotransferrin OS=Sus scrofa
Accession: TRFE_PIG
Sequence: L.VFEAGLAPYNLKPVVAEF.Y
28

## Slide 29
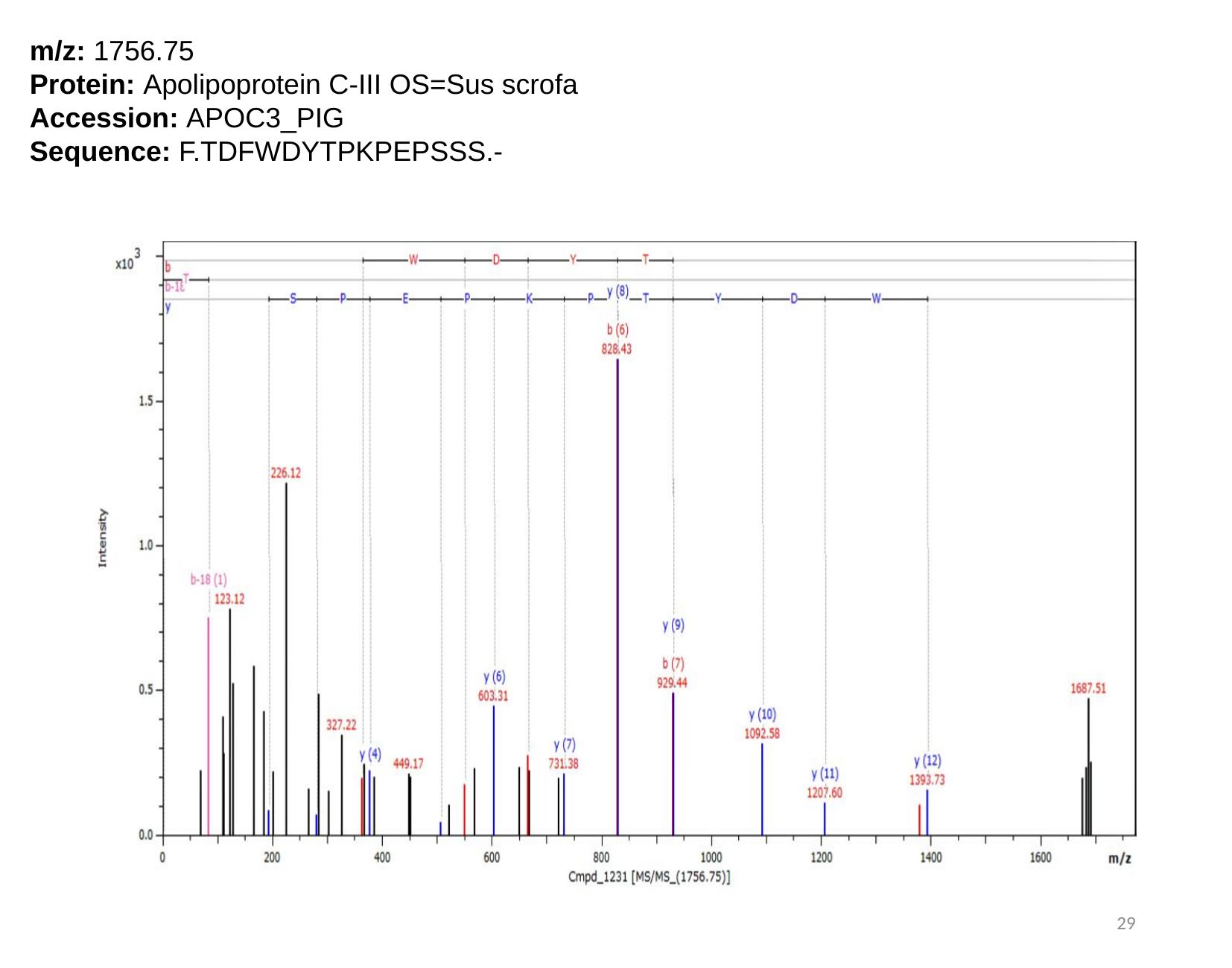

m/z: 1756.75
Protein: Apolipoprotein C-III OS=Sus scrofa
Accession: APOC3_PIG
Sequence: F.TDFWDYTPKPEPSSS.-
29

## Slide 30
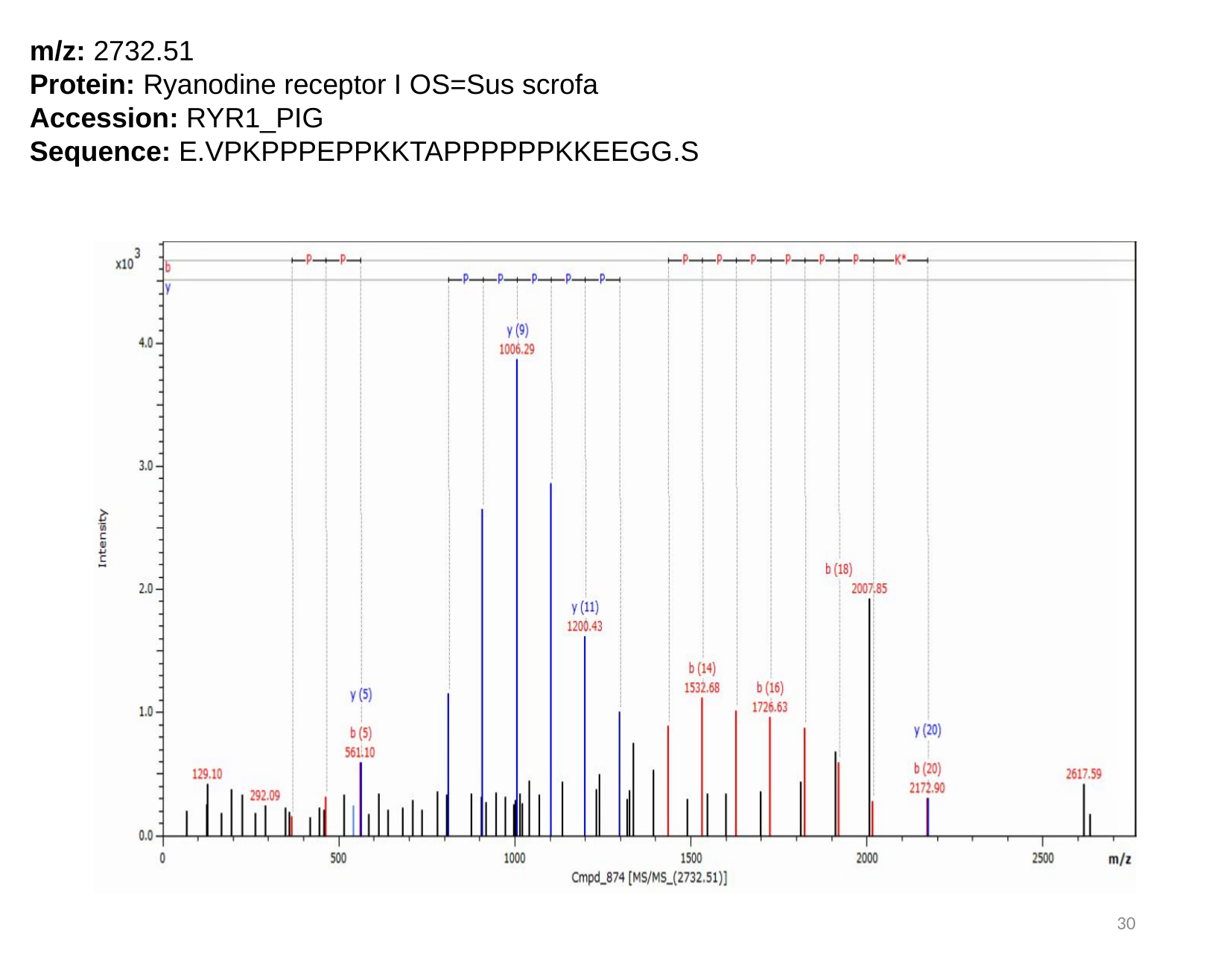

m/z: 2732.51
Protein: Ryanodine receptor I OS=Sus scrofa
Accession: RYR1_PIG
Sequence: E.VPKPPPEPPKKTAPPPPPPKKEEGG.S
30

## Slide 31
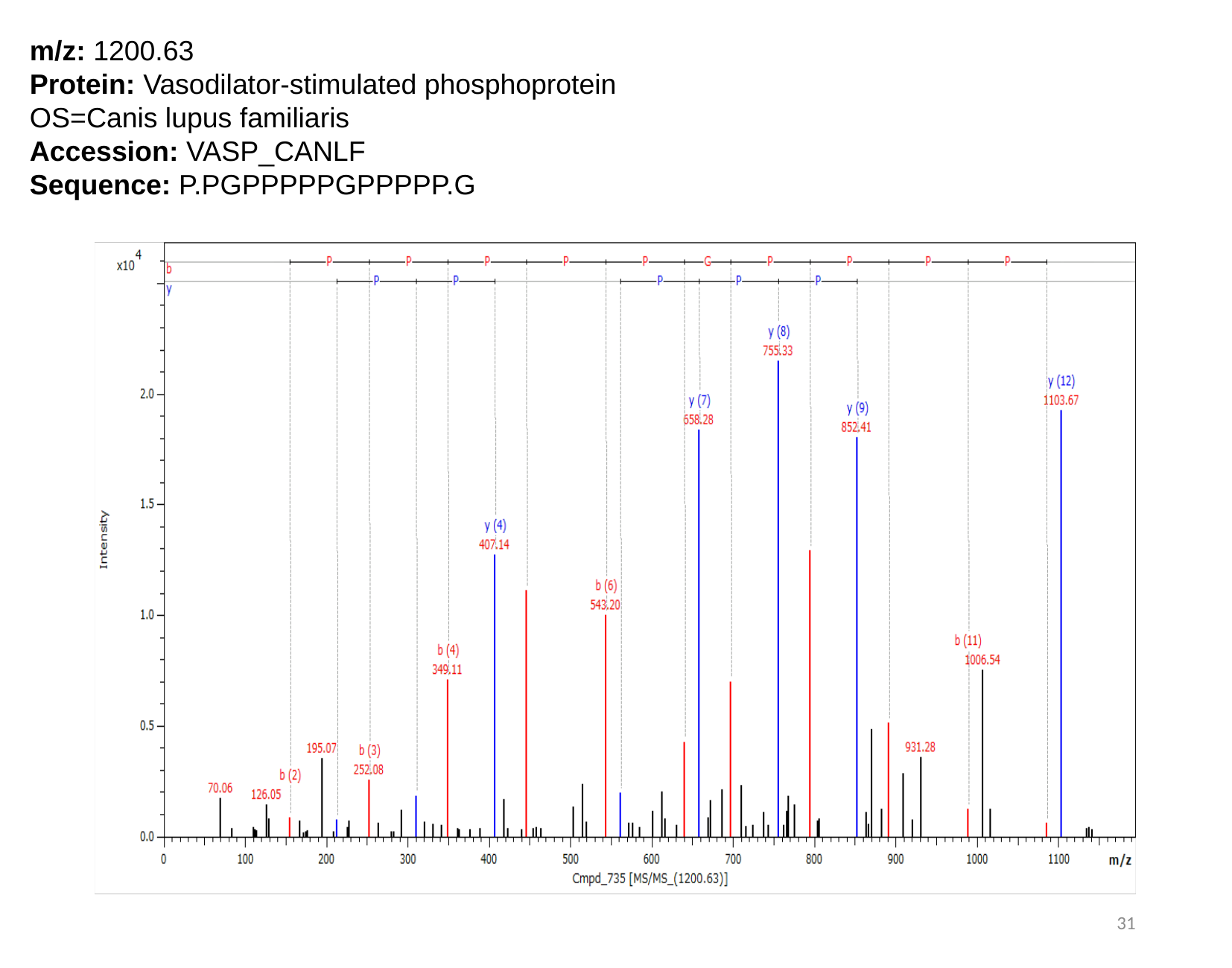

m/z: 1200.63
Protein: Vasodilator-stimulated phosphoprotein
OS=Canis lupus familiaris
Accession: VASP_CANLF
Sequence: P.PGPPPPPGPPPPP.G
31
