# Supplementary material for: Chronological Profiling of Plasma Native Peptides after Hepatectomy in Pigs: Toward the Discovery of Human Biomarkers for Liver Regeneration
Source: PLoS One. 2017 Jan 6;12(1):e0167647. doi: 10.1371/journal.pone.0167647 (PMC5218562; doi:10.1371/journal.pone.0167647)
Supplement: S1 Table — Four patients who underwent major hepatectomy without pathological liver fibrosis (F0 or F1) [17], and had an uneventful postoperative course without PHLF, were included. *1The primary tumor originated in stomach. *2The definition of PHLF is an increase in the international normalized ratio of prothrombin time and concomitant hyperbilirubinemia (according to the normal limits of the local laboratory) on or after postoperative day 5 [4]. PHLF, posthepatectomy liver failure; nonB, nonC HCC, hepatocellular carcinoma without hepatitis B or C viral infection. (DOCX) [file pone.0167647.s003.docx]

**S1 Table. Characteristics of patients analyzed in this study.**

|  | Case 1 | Case 2 | Case 3 | Case 4 |
| --- | --- | --- | --- | --- |
| Age | 63 | 74 | 79 | 78 |
| Sex | male | male | male | male |
| Disease | Metastatic liver cancer*^1^ | nonB, nonC HCC | nonB, nonC HCC | nonB, nonC HCC |
| Liver fibrosis | F0 | F0 | F1 | F0 |
| Procedure | Left hepatectomy | Right hepatectomy | Right hepatectomy | Right hepatectomy |
| Blood loss (ml) | 210 | 3000 | 3750 | 4041 |
| PHLF^*2^ | - | - | - | - |

Four patients who underwent major hepatectomy without pathological liver fibrosis (F0 or F1),[17] and had an uneventful postoperative course without PHLF, were included.

*^1^Primary tumor origin was stomach.

^*2^The definition of PHLF is an increase in the international normalized ratio of prothrombin time and concomitant hyperbilirubinemia (according to the normal limits of the local laboratory) on or after postoperative day 5.[4] PHLF, posthepatectomy liver failure; nonB, nonC HCC, hepatocellular carcinoma without hepatitis B or C viral infection.
