# Supplementary material for: Chronological Profiling of Plasma Native Peptides after Hepatectomy in Pigs: Toward the Discovery of Human Biomarkers for Liver Regeneration
Source: PLoS One. 2017 Jan 6;12(1):e0167647. doi: 10.1371/journal.pone.0167647 (PMC5218562; doi:10.1371/journal.pone.0167647)
Supplement: S6 Table — (A) More peptide fragments were detected in PV than in CV. This result might reflect PV containing more humoral factors than CV that are capable of stimulating DNA synthesis after PHx. (B) More peptide fragments were observed in serum than in plasma. The lower stability of peptide fragments in serum is considered to result from ex vivo multiprotease activities due to clotting. The SVM analyses discriminated PV and CV, and serum and plasma with a cross-validation accuracy of more than 88 and 93% at all timings, respectively. In the present study, the plasma samples were analyzed to detect endogenous peptides. Considering the difficulty in repeated sampling from PV, which otherwise might be more informative, the CV specimens were used for further studies. *Differences with AUC calculated by ROC curve analysis > 0.8 were considered to be significant. Peaks were described with m/z values. Five and 4 pigs were used for analyses of (A) and (B), respectively. PV, portal vein; CV, central vein; AUC, area under the curve; ROC, receiver operating characteristics; m/z, mass to charge ratio. (DOCX) [file pone.0167647.s008.docx]

**S6 Table.** The peak list that had a significant difference* in intensity regarding sampling points and methods.

(A)

|  | PV > CV | CV > PV |
| --- | --- | --- |
| Pre | 4615 |  |
| 0 min | 3538, 3852 |  |
| 1 h | 2898, 3000, 3028 |  |
| 3 h | 3000, 3028, 4615 | 2041, 11202 |
| 168 h | 2898, 2920, 3000, 3028 | 2531 |

(B)

|  | Serum > Plasma | Plasma > Serum |
| --- | --- | --- |
| Pre | 2041, 2742, 2857 | 2878, 11128 |
| 0 min | 2041, 2857, 3852 | 2878 |
| 24 h | 2041, 2857, 3627, 3852, 4615 | 2878 |
| 48 h | 2041, 2857 | 2878 |
| 120 h | 2041, 2857, 3042, 9225 | 2878, 2894 |

(A) More peptide fragments were detected in PV than in CV. This result might reflect PV containing more humoral factors than CV that are capable of stimulating DNA synthesis after PHx. (B) More peptide fragments were observed in serum than in plasma. The lower stability of peptide fragments in serum is considered to result from ex vivo multiprotease activities due to clotting. The SVM analyses discriminated PV and CV, and serum and plasma with a cross-validation accuracy of more than 88 and 93% at all timings, respectively. In the present study, the plasma samples were analyzed to detect endogenous peptides. Considering the difficulty in repeated sampling from PV, which otherwise might be more informative, the CV specimens were used for further studies. *Differences with AUC calculated by ROC curve analysis > 0.8 were considered to be significant. Peaks were described with *m/z* values. Five and 4 pigs were used for analyses of (A) and (B), respectively. PV, portal vein; CV, central vein; AUC, area under the curve; ROC, receiver operating characteristics; *m/z*, mass to charge ratio.
